# Integrating Microbiome Analysis, Metabolomics, Bioinformatics, and Histopathology to Elucidate the Protective Effects of Pomegranate Juice against Benzo-alpha-pyrene-Induced Colon Pathologies

**DOI:** 10.3390/ijms241310691

**Published:** 2023-06-27

**Authors:** Heba Attia, Shahira A. ElBanna, Rania A. Khattab, Mohamed A. Farag, Aymen S. Yassin, Ramy K. Aziz

**Affiliations:** 1 Department of Microbiology and Immunology, Faculty of Pharmacy, Cairo University, Cairo 11562, Egypt; heba.mohamed@pharma.cu.edu.eg (H.A.); shahira.ahmed@pharma.cu.edu.eg (S.A.E.); rania.khatab@pharma.cu.edu.eg (R.A.K.); 2 Center for Genome and Microbiome Research, Faculty of Pharmacy, Cairo University, Cairo 11562, Egypt; 3 Department of Pharmacognosy, Faculty of Pharmacy, Cairo University, Cairo 11562, Egypt; mohamed.farag@pharma.cu.edu.eg; 4 Microbiology and Immunology Research Program, Children’s Cancer Hospital Egypt 57357, Cairo 11617, Egypt

**Keywords:** toxicomicrobiomics, polyphenols, polycyclic aromatic hydrocarbons, bioinformatics, multivariate analysis, multi-omics, systems toxicology, systems biology, cancers

## Abstract

Polycyclic aromatic hydrocarbons, e.g., benzo[a]pyrene (BaP), are common dietary pollutants with potential carcinogenic activity, while polyphenols are potential chemopreventive antioxidants. Although several health benefits are attributed to polyphenol-rich pomegranate, little is known about its interaction with BaP. This study integrates histochemical, microbiomic, and metabolomic approaches to investigate the protective effects of pomegranate juice from BaP-induced pathologies. To this end, 48 Sprague–Dawley rats received, for four weeks, either pomegranate, BaP, both, or neither (*n* = 12 rats per group). Whereas histochemical examination of the colon indicated tissue damage marked by mucin depletion in BaP-fed animals, which was partially restored by administration of pomegranate juice, the fecal microbiome and metabolome retained their resilience, except for key changes related to pomegranate and BaP biotransformation. Meanwhile, dramatic microbiome restructuring and metabolome shift were observed as a consequence of the elapsed time (age factor). Additionally, the analysis allowed a thorough examination of fecal microbiome–metabolome associations, which delineated six microbiome clusters (marked by a differential abundance of *Lactobacillaceae* and *Prevotellaceae*, *Rumincococcaceae*, and *Erysipelotrichaceae*) and two major metabolome clusters (a sugar- and amino-acids-dominated metabotype vs. a cluster of fatty acids and hydrocarbons), with sugar alcohols maintaining a unique signature. In conclusion, using paired comparisons to minimize inter-individual animal variations allowed the dissection of temporal vs. treatment-derived variations. Microbiome–metabolome association clusters may be further exploited for metabotype prediction and gut-health biomarker discovery.

## 1. Introduction

The incidence of cancer is on the rise in developing countries [1,2] as a result of demographic shifts, climate change, pollution, and the adoption of cancer-associated lifestyle choices [3]. The global cancer burden rose to an estimated 19.3 million new cases (18.1 million cases excluding nonmelanoma skin cancer) and 10 million cancer deaths (9.9 million excluding nonmelanoma skin cancer) in 2020 [4], and the onset of cancer is believed to be more common among individuals <50 years [5].

Colorectal cancer (CRC) is the second leading cause of cancer death in the world, and its burden is expected to increase by 60% to more than 2.2 million new cases and 1.1 million cancer deaths by 2030 [6]. In Egypt, a high incidence of CRC has been reported among the younger population (<40 years) [7,8,9].

While hereditary factors are behind up to 25% of CRC cases [10], sporadic gene mutations account for nearly 70% of reported cases [11,12], and it is thought that exposure to environmental carcinogens is involved in the incidence of these genetic mutations [13,14,15]. Other reports indicate that diet contributes to more than 80% of known colorectal cancer cases [16,17].

Benzo[a]pyrene (BaP) is an environmental toxicant that belongs to the category of group I human carcinogens (“carcinogenic to humans”), designated by the International Agency for Research on Cancer [18]. It is also one of the target compounds defined by the Environmental Protection Agency’s strategy to monitor and control bioaccumulative and toxic environmental pollutants [19].

BaP, the prototype and the most extensively characterized compound of polycyclic aromatic hydrocarbons (PAHs) [20], is mainly generated by incomplete combustion or pyrolysis of organic matter. The main routes of exposure to PAHs are inhalation of polluted air, smoking, and diet [21,22,23].

Human exposure to BaP mainly occurs through the consumption of grilled, smoked, barbecued, deep-fried, or broiled foods because BaP can be produced by the pyrolysis of amino acids, fatty acids, and carbohydrates during the cooking process [24,25].

Because of its serious genotoxic, cytotoxic, mutagenic, and carcinogenic effects [26,27,28,29,30], as well as its abundance in the environment [23,31,32], BaP is one of the most studied toxicants linked to the development of colon tumors [33,34,35]. Several studies have reported an association between estimated dietary intake of BaP and risk of colorectal adenoma [16,36,37,38].

On the other side of the food spectrum, plants and their constituents offer attractive nutritional constituents to prevent cancer. While they have traditionally been used for preventing diseases, including cancer, the emerging concept of combination chemoprevention by multiple agents (functional foods or ‘nutraceuticals’) is becoming increasingly attractive, opening the possibility for dietary modification of colon cancer risk [39,40,41,42,43]. One such family of plant products is polyphenols, present in green tea, green coffee, and pomegranate, among many other plants. The health benefits of pomegranate (*Punica granatum* L.), as a main source of dietary polyphenols, have been mostly attributed to its rich hydrolyzable tannins, including gallotannins and ellagitannins [44,45]. Although pomegranate is a rich source of bioactive compounds, the synergistic action of those compounds in the natural juice is believed to be superior to the action of individual or purified ingredients [46,47].

Pomegranate ellagitannins are scarcely absorbable [48], and they reach the colon where the gut microbiota catabolizes them into more bioavailable metabolites (urolithins), which in turn can modulate the gut microbiota composition and activity with a subsequent beneficial impact on an individual’s health [49,50]. Thus, polyphenol-rich pomegranate has a prebiotic-like effect, which has been associated with improvements in host metabolism and overall health status [51].

Additionally, pomegranate exerts protective effects against multiple cancers, both in vitro and in vivo, in several animal models [52,53,54,55,56,57]. Several studies have reported a specific role for pomegranate against colon cancer [58], both in vitro [44,59,60,61] and in vivo [62,63].

While plenty of reports affirm a microbiota-related procarcinogenic role for BaP, as well as direct and microbiota-related anti-cancer effects for pomegranate [64], little experimental evidence is available on the role of pomegranate as a protective agent in the presence of BaP. In addition, even less is known about the causality of such a complex role of the two chemicals, i.e., whether the microbiome variations associated with BaP and pomegranate are causal, consequential, or coincidentally associated with tumor markers or colon health. 

Thus, this study was devised to investigate, using a systems approach combining histological and high-throughput microbiome and metabolome analyses, the protective effects of pomegranate juice on BaP-induced pathologies of the colon in a Sprague–Dawley rat model, as well as its potential effects on the gut microbiota compositional and metabolic balance. Using the animal model allowed interrogating causality and age/temporal variations, as well as characterizing the impact of each of the two chemicals and their combination on the rat gut microbiome and metabolome.

We found a partial tissue-protective effect by pomegranate from damage induced by BaP, as well as a major age-dependent compositional restructuring of the microbiome and metabolome. Additionally, we resolved distinct, yet less dramatic, microbiome/metabolome modulatory effects by the different chemicals used, and we proposed distinctive microbiome/metabolome clusters.

## 2. Results

This study aimed to investigate the potential protective effects of pomegranate juice from BaP-induced pathologies in the colons of Sprague–Dawley rats. It also scrutinized microbiome and metabolome changes in this animal model upon ingestion of pomegranate juice, BaP, or their combination.

At the start of the experiment, 48 Sprague–Dawley rats of comparative weights (Figure S1A) were randomized, according to their fecal β-glucuronidase activity (Figure S2A), into 12 cages, assigned to four treatment groups (i.e., each treatment group consisted of 12 rats housed in three different cages) and acclimatized to the diet and laboratory environment for 10 days. After the acclimatization period, different groups were fed different treatment regimens for four weeks: an untreated group (untreated or U) was fed a standard AIN76 diet; a pomegranate group (Pom or P) was fed the standard diet + 50 mg gallic acid equivalent (GAE)/kg/day pomegranate juice; a benzo[a]pyrene group (BaP or B) was fed the standard diet + 150 μg/Kg/day benzo[a]pyrene (dissolved in peanut oil); and a pomegranate + BaP group (PomBaP or PB) was fed the standard diet + 50 mg GAE/kg/day pomegranate + 150 μg/Kg/day BaP (also dissolved in peanut oil). Of note, rats in both the untreated and Pom groups also received peanut oil by oral gavage, because that oil was the solvent used in the BaP and PomBaP groups.

After four full weeks of daily ingestion of the standard diet with or without the tested substances, the overall difference in each animal’s weight was recorded, the glucuronidase activity in the stool was colorimetrically determined, and colons were dissected out of all rats, pooled by treatment group, and examined histologically. Additionally, comprehensive microbiome profiling and gas chromatography metabolomic analysis were performed on fecal samples collected from the rats before and after the 4-week treatment.

### 2.1. Negligible Changes in Animal Weights along the Experiment

**Figure 1:**
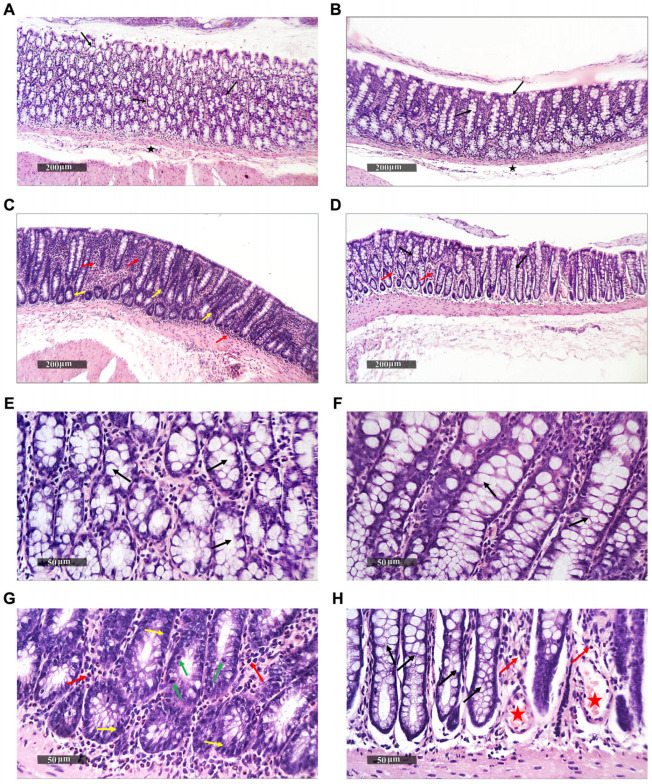
Histopathological alterations in rat colons upon different treatments (hematoxylin and eosin staining, 400×/scale bar 200 μm (**A**–**D**); 50 μm (**E**–**H**). (**A**,**E**): untreated rats; (**B**,**F**): pomegranate-fed rats; (**C**,**G**): BaP-fed rats; (**D**,**H**): pomegranate + BaP-fed rats. Black arrows: intact goblet cells; black star: intact submucosal layers; yellow arrows: hyperplastic and dysplastic changes; red arrows: inflammatory cell infiltrate; green arrows: mitosis; red stars: congested mucosal blood vessels.

**Figure 2:**
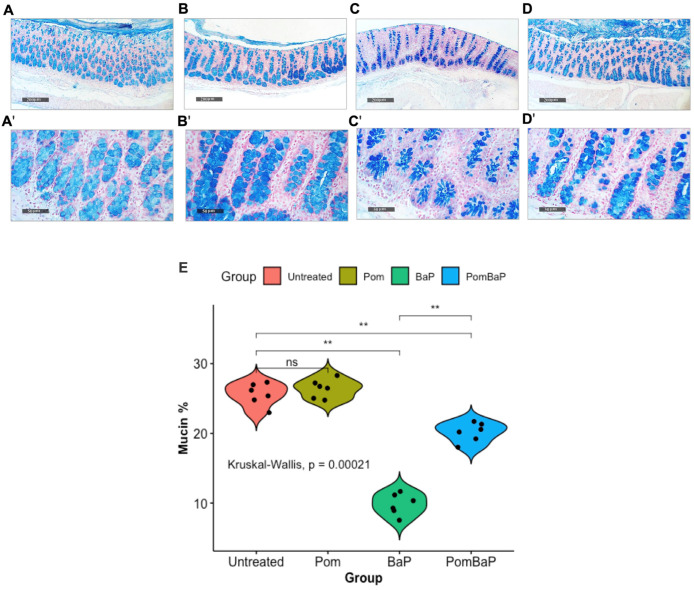
(**A**–**D**): Histopathological alterations in rat colons upon different treatments (stained by Alcian blue pH 2.5, 400×/scale bar 200 μm (**A**–**D**); 50 μm (**A′**–**D′**). (**A**,**A′**): untreated rats; (**B**,**B′**): pomegranate-fed rats; (**C**,**C′**): BaP-fed rats; (**D**,**D′**): pomegranate + BaP-fed rats. (**E**): Beanplots comparing the mucosal acidic mucin levels, expressed as the percentage of mucosal reactive mucin areas between different treatment groups (*n* = 6 per group). Differences between groups were analyzed for statistical significance by the Kruskal–Wallis test, followed by post-hoc pairwise Wilcoxon testing. ns = non-significant (*p* value > 0.05); ** = *p* value < 0.005.

The average weight difference after four weeks was 2.62 ± 27.8 g; however, this weight change was neither significantly different between treatment groups nor between the week 0 and week 4 time points (Figure S1B), reflecting a lack of observable gross physiological differences between rats, as well as developmental and dietary stability. However, colonic examination demonstrated marked histological differences between the rats (Figure 1 and Figure 2).

### 2.2. Histopathological Alterations in Rat Colons upon Treatment

To determine relevant phenotypic alterations in the intestinal lumen and tissues, we microscopically examined hematoxylin and eosin (H&E)-stained rat colons from the four groups (Figure 1), and we quantified goblet cell-derived reactive acidic mucins by Alcian blue (pH 2.5) staining (Figure 2).

The histopathological examination indicated that the colonic walls of both the untreated and pomegranate-fed rat groups had well-organized histological features, with intact intestinal crypts (Figure 1A,B,E,F). The crypts had abundant goblet cells, alternated with many enterocytes, with intact nuclear and subcellular details, intact propria, and intact submucosal layers, without abnormal infiltrates. On the other hand, the colons of BaP-fed rats had obvious histopathological changes. Focal areas of hyperplastic and dysplastic changes were common in the lining epithelium of intestinal crypts, with dark basophilic cytoplasms and oval basally situated nuclei (Figure 1C,G). Additionally, these colons were characterized by an increased nuclear cytoplasmic ratio (N/C) with many mitotic figures, minimal differentiated mature goblet cells, and marked interstitial inflammatory cell infiltrate (Figure 1G).

As for the rats fed with both substances (the PomBaP group), dysplastic changes in the lining epithelium of intestinal crypts were markedly reduced, with many intact enterocytes and apparent intact mature goblet cells. An inflammatory cell infiltrate was still observed in these rats, accompanied by mild congested mucosal blood vessels (Figure 1D,H).

The highest level of reactive mucosal acidic mucin was observed in the pomegranate-fed rats, followed by the untreated rats, whereas a significant reduction in reactive mucin was observed in the BaP-fed rats (Figure 2C,E). In the PomBaP-fed rats, mucin levels were significantly higher (median ~ 20%) than in the BaP-fed rats (post-hoc Wilcoxon test *p* value = 0.002), yet they were not fully restored to the mucin levels of the Pom or untreated groups (median ~ 25%, Figure 2E).

### 2.3. Uneven Increase in β-Glucuronidase Activity after Four Weeks

**Figure 3:**
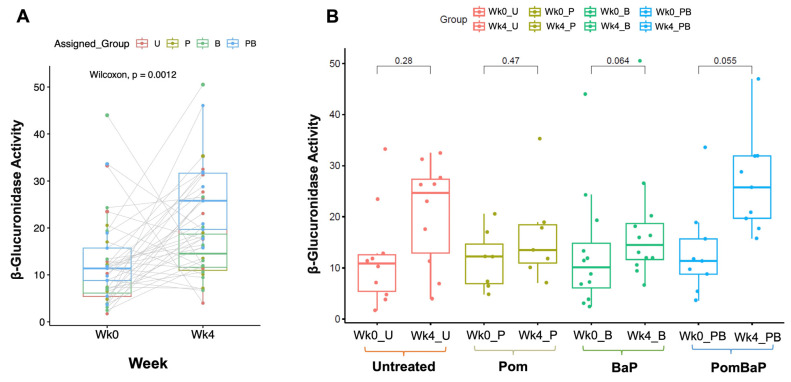
Paired analysis of fecal β-glucuronidase enzyme activity at the start (Wk0) and end (Wk4) of the experiment. *Y*-axis: β-glucuronidase activity expressed as mg of 4-Nitrophenyl β-D-glucuronide (PNPG)/mg feces per minute. (**A**) The paired analysis was conducted on all rats that survived until the end of the experiment, with each dot representing one rat. Gray lines link the value pairs for each rat. (**B**) The paired analysis was conducted within each assigned treatment group (only rats that survived until the end of the experiment are shown). Statistical significance for both comparisons was assessed by paired Wilcoxon test, and all *p* values are shown. **Legend: U** = untreated; **P** = pomegranate; **B** = benzo[a]pyrene; **PB** = pomegranate + benzo[a]pyrene.

Glucuronidases are key xenobiotic-modifying bacterial enzymes that are enriched in specific bacterial classes and have been reported to increase in the microbiota in association with some types of cancer. For this reason, we ensured that the rats were randomized for week 0 fecal glucuronidase activity, which was determined right before the feeding system was initiated (Figure S2A indicates no significant difference between cages). After the four-week experiment, the glucuronidase activity significantly increased in the entire rat population of the experiment (paired Wilcoxon test *p* value = 0.0012, Figure 3A), yet there was no significant difference in week 4 glucuronidase activity between different groups (Figure S2B). Peculiarly, the within-group increase in β-glucuronidase activity did not reach statistical significance (Figure 3B) but tended to be significant in the Pom + BaP-fed rats (paired Wilcoxon *p* value 0.055), as eight out of nine rats in this group had modest to large increases in β-glucuronidase activity (paired lines between light blue dots in Figure 3A).

### 2.4. Microbiome Profiling by 16S rRNA Amplicon Sequencing

To investigate any potential effects exerted by the pro-carcinogenic BaP, anti-carcinogenic pomegranate juice, and their combination on the rat gut microbiota, we sequenced the DNA extracted from 24 samples collected from 12 cages before and after the 4-week treatment period. Each sample represented carefully collected, pooled animal feces from a different cage, and thus each treatment group (untreated, Pom, BaP, and PomBaP) included three different cages (biological replicates—with all live animals therein), while the control included week 0 samples from all 12 cages.

The raw sequencing reads were quality filtered, pre-processed, and then analyzed by MOTHUR software against the SILVA 16S rRNA database (v.123). MOTHUR analysis filtered and classified 171,078 reads, with an average of 7128 reads per sample, that were collectively assigned to 367 non-redundant taxonomic units of different taxonomic levels (including 17 phyla, 32 classes, 47 orders, 74 families, and 195 genera). Subsequently, the assigned taxa were compared among samples and sample groups at different taxonomic levels. All data were then re-filtered, statistically analyzed, and visualized by MicrobiomeAnalyst software [65] as follows.

Analyzed reads were normalized across samples (as a fraction of total reads) and rarefied to the minimum library size (1369), and 11,150 low-abundance features + 20 low-variance features were removed after a low-variance filter was applied (detailed in the Materials and Methods Section).

At the phylum level, MOTHUR initially identified 17 phyla, 15 of which were retained by MicrobiomeAnalyst (as two of the initially classified phyla were filtered out for low abundance). Four phyla were the most predominant in all samples: Bacteroidetes (with mean relative abundance = 65.9% of all classified reads and 67.8% of filtered classified reads), followed by Firmicutes (with mean relative abundance = 26.4% of all classified reads and 17.4% of filtered classified reads), Tenericutes (with mean relative abundance = 5.8% of all classified reads and 14.3% of filtered classified reads), and Actinobacteria (with mean relative abundance = 1.6% of all classified reads and 0.52% of filtered ones). Eleven other phyla were found in low proportions (Figure S3A). At the genus level, 193 genera were observed, 16 of which were the predominant ones (Figure S3B), while 176 other genera were found in low proportions (Supplementary Data File S1).

### 2.5. Alterations in Fecal Microbiota Diversity across Sample Groups

The alpha diversity of the rat fecal microbiota was estimated, by different indices, at the genus level. Three out of the four calculated alpha diversity indices (observed OTUs, Shannon, and Simpson) were significantly different between control samples and each of the following treatment groups: untreated, Pom, and BaP (Figure S4A). The same three indices were significantly different between the week 0 and week 4 samples (Figure S4C). However, when the significantly different control samples were excluded from the comparison, because they all belong to week 0, no other significant difference was observed between the alpha diversity metrics of any of the week 4 treatment groups (Figure S4B).

**Figure 4:**
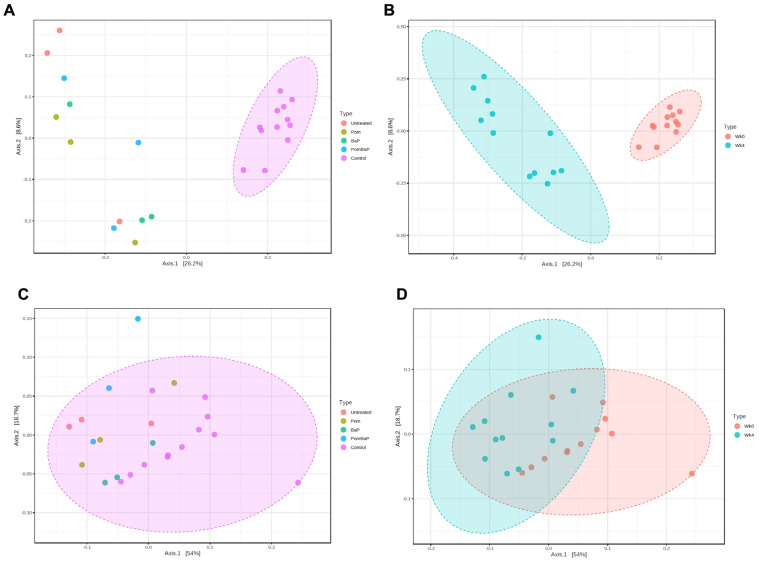
Beta diversity, visualized by PCA of Bray–Curtis distances between different features (**A**,**B**) or different genera (**C**,**D**). (**A**) Comparison between treatment groups: only control samples clustered together—permutational multivariate analysis of variance (PERMANOVA): F-value = 2.716, R-squared = 0.363, *p* value = 0.001. (**B**) Comparison between week 0 and week 4 samples: the samples were separated into two distinct clusters—PERMANOVA F-value = 7.047, R-squared = 0.242, *p* value = 0.001. (**C**) Comparison between treatment groups, at genus level: no separation between groups, but a general cluster was formed with PERMANOVA F-value = 2.746, R-squared = 0.366, *p* value = 0.004. (**D**) Comparison between week 0 and week 4 samples at genus level: overlapping clusters between the two age groups—PERMANOVA F-value = 7.783, R-squared = 0.261, *p* value = 0.001.

The gut microbiota beta diversity, assessed through principal component analysis (PCA) of Bray–Curtis distances of all sequence variants (feature-level analysis), indicated no segregation between different treatment groups, except for the control group (week 0) samples (Figure 4A). Thus, the time attribute could be the more discriminant factor. Indeed, the 24 microbial communities were separated into two distinct clusters based on age/temporal factor: week 0 vs. week 4 (Figure 4B). At the genus level, the same clustering pattern was retained, but to a lesser extent (Figure 4C,D).

Overall, both alpha and beta diversity analyses strongly highlighted the age or temporal factor as a major predictor of microbiome composition and diversity. Meanwhile, despite observed cage-to-cage variability (Figure S3), the cage factor did not seem to consistently affect microbiome profiles across treatment groups, and no specific clustering by cage could be seen among samples (Figure S5). The strong temporal factor does not discount that some changes are caused by the treatment type (i.e., group factor), which seemed less pronounced than, but distinct from, the age factor, as detailed below.

### 2.6. Alterations in Fecal Microbiome Profiles across Sample Groups

**Figure 5:**
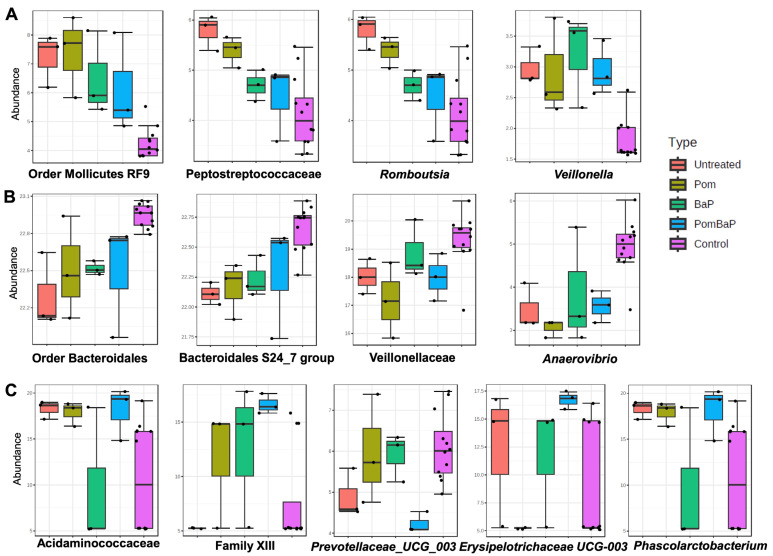
Taxa with significant differences in relative abundance among the five groups. Thirteen significant taxa in different samples (caused by both age and treatment factors) identified by LEfSe, with an applied *p* value cutoff of 0.05 and adjusted by the false discovery rate (FDR) method. (**A**) Taxa that are relatively less abundant in the control group; (**B**) taxa that are relatively more abundant in the control group; (**C**) taxa that are significantly different between different treatment groups. *Y*-axis: Relative abundance expressed as log-transformed counts.

Upon comparison of the microbiome profiles among all five groups (the four treatment groups at week 4, including three samples/cages per group, as well as the control samples, including all 12 cages of week 0), 19 taxa (Figure S6) of different taxonomic levels were deemed statistically significantly different between the groups, as calculated by linear discriminant analysis effect size (LEfSe). A shorter list of the taxa is provided in Figure 5.

Among these differentially abundant taxa, order Mollicutes RF9 (class Mollicutes); family *Peptostreptococcaceae* and genus *Romboutsia* (class Clostridia, order Clostridiales); and genus *Veillonella* were less abundant in the control group (Figure 5A), while order Bacteroidales and family Bacteroidales S24-7 group (phylum Bacteroidetes, class Bacteroida) and family *Veillonellaceae* and genus *Anaerovibrio* were relatively more abundant in the control group (Figure 5B).

Family *Acidaminococcaceae* and genus *Phascolarctobacterium*; *Family XIII*; and genus *Erysipelotrichaceae UCG-003* were relatively more abundant in the PomBaP group, while genus *Prevotellaceae UCG_003* was relatively less abundant in the same group (Figure 5C).

#### 2.6.1. Influence of Temporal, Age-Related Factors on Fecal Microbiome Composition

Because the above analysis included two major experimental factors, namely time (i.e., week 4 vs. week 0, reflecting 28 days of lifespan) and the treatment factor (three different feeding systems, in addition to the solvent-only or untreated group), we compared all samples, lumped by sampling week.

**Figure 6:**
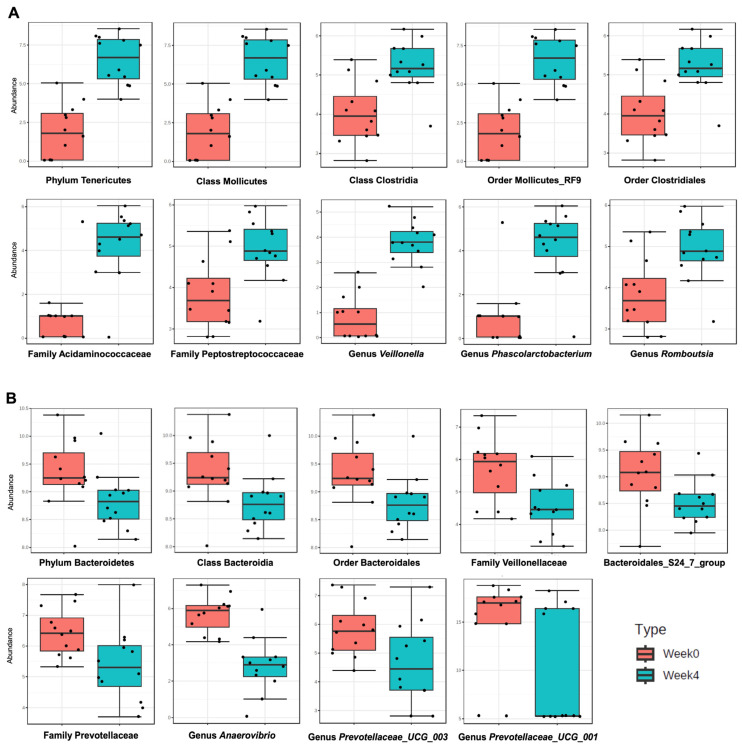
Significant taxa, in week 0 and week 4 samples, identified by -LEfSe-, with an applied *p* value cutoff of 0.05, and a multiple testing adjustment by the FDR method. (**A**) Taxa that are significantly more abundant at the end of week 4. (**B**) Taxa that are significantly less abundant at the end of week 4. *Y*-axis: Relative abundance expressed as log-transformed counts. Note: some boxplots are almost identical, and this is not by error. It reflects that almost all classified Tenericutes are from the order Mollicutes, which is under class Mollicutes. Likewise, all Clostridia detected seem to be from the order Clostridiales.

This comparison indicated an increase in the relative abundance of some taxa with time/age. The abundance of phylum Tenericutes; classes Mollicutes and Clostridia; orders Mollicutes RF9 and Clostridiales; families *Acidaminococcaceae* and *Peptostreptococcaceae*; and genera *Veillonella*, *Phascolarctobacterium*, and *Romboutsia* seemed to increase at the end of week 4 (Figure 6A). On the other hand, the abundance of phylum Bacteroidetes; class Bacteroidia; order Bacteroidales; families *Prevotellaceae*; Bacteroidales S24-7 group; and *Veillonellaceae*, genera *Anaerovibrio*, *Prevotellaceae UCG-001*, and *Prevotellaceae UCG-003* seemed to decrease in week 4 samples (Figure 6B).

#### 2.6.2. Alterations in Fecal Microbiome Profiles in Response to Daily Ingestion of Pom, BaP, and PomBaP

Having examined the overall variability between different groups and between the week 0 and week 4 samples, we set out to resolve differences that are only contributed by the treatment factor. This required normalization to week 0 (i.e., calculating a week 4-to-week 0 abundance ratio for different microbial taxa). Because it was not mathematically possible to calculate ratios for taxa that were not detected on week 0 (i.e., having zero relative abundance values), the week 4/week 0 ratio was only calculated for taxonomic groups with non-zero values in week 0 samples (92 taxonomic units of all levels).

**Figure 7:**
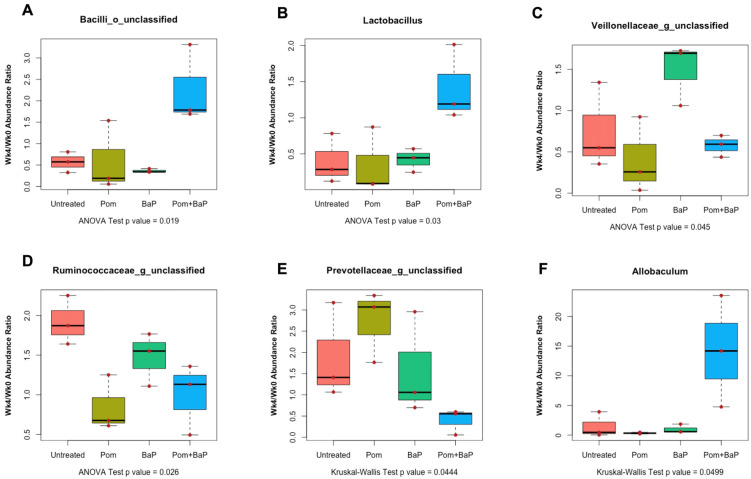
Significantly different taxa between different treatment groups of week 4 (*n* = 3 cages per group) normalized to their corresponding week 0 samples, analyzed by ANOVA (**A**–**D**) or Kruskal–Wallis test (**E**–**F**). Of note, the only taxon that had a *p* value < 0.05 with both tests was *Allobaculum* (Kruskal–Wallis *p* value = 0.0499; ANOVA *p* value = 0.022). A full set of all comparisons, including redundant abundance plots for different taxonomic levels of the same organisms, is available in Supplementary Figure S7.

When the samples from the four different treatment groups of week 4 were normalized to their corresponding samples of week 0 with non-zero relative abundance, we found that family *Lactobacillaceae* (mainly *Lactobacillus*), non-*Lactobacillaceae* members of class Bacilli (i.e., Bacilli of unclassified order, family, or genus), and genus *Allobaculum* were significantly more abundant in PomBaP-fed rats, while unclassified *Ruminococcacae* were more abundant in the untreated rats. Additionally, unclassified genera of *Veillonelleacae* were significantly (ANOVA *p* value < 0.05) more abundant in the BaP-fed rats (Figure 7). After repeating the same analysis with non-parametric statistical testing (Kruskal–Wallis test followed by Dunn’s or paired Wilcoxon post-hoc tests), only genus *Allobaculum* and family *Prevotellaceae* (unclassified genera) were deemed statistically significantly different between groups (*p* value < 0.05). *Allobaculum* abundance was significantly higher in week 4 samples from the PomBap group and significantly lower in the week 4 Pom group than all other groups, while unclassified *Prevoteallaceae* were more abundant in the Pom group of week 4 than in the PomBaP group.

### 2.7. Alterations in Fecal Metabolome across Sample Groups

To understand the metabolic consequence of the four-week feeding scheme and its associated microbiome alterations, we further analyzed 72 fecal samples (three technical replicates of 24 samples, representing the 12 cages before and after the experiment) by gas chromatography coupled with mass spectrometry detection (GC-MS) to dissect the changes in metabolomic profiles before and after treatment, and between different treatment groups. Metabolites detected as trimethylsilylated derivatives were mostly low-molecular-weight primary metabolites, such as organic acids, amino acids, fatty acids, esters, alcohols, nitrogenous compounds, phenolic acids, sterols, and sugars, in addition to aliphatic and aromatic hydrocarbons, some of which are likely derived from metabolism of pomegranate juice by gut microbiota.

**Figure 8:**
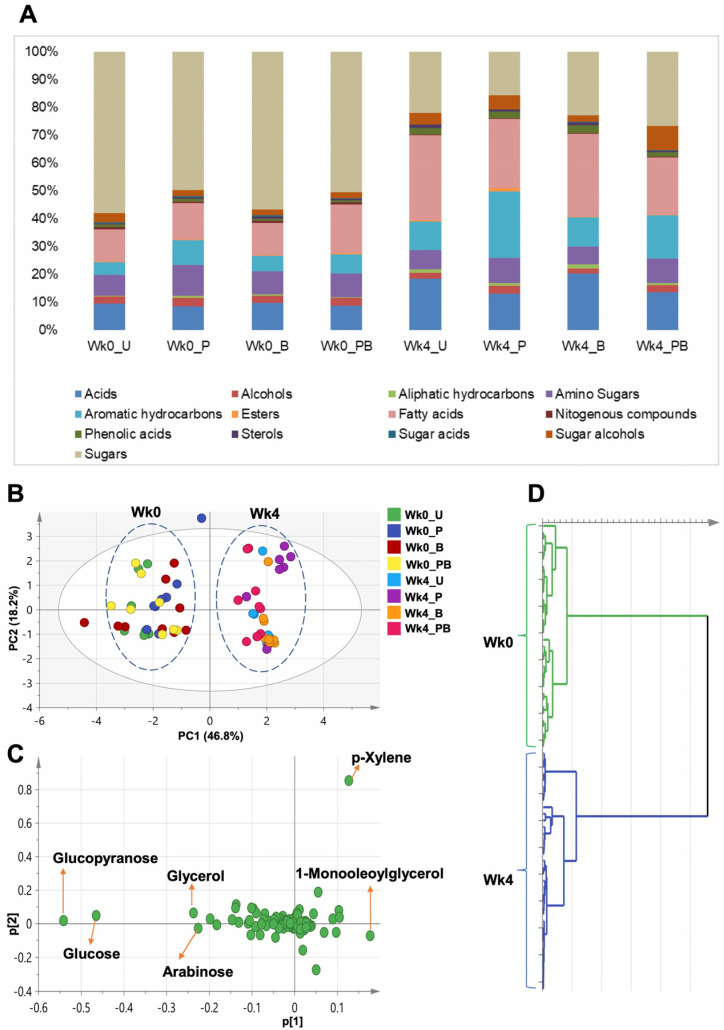
Metabolomic comparison of all samples from week 0 and week 4. (**A**) Levels of metabolite classes detected by GC-MS for samples from week 0 (Wk0_U, Wk0_P, Wk0_B, Wk0_PB) and from week 4 (Wk4_U, Wk4_P, Wk4_B, Wk4_PB). (**B**,**C**) PCA for eight sample groups (four groups representing week 0 and four groups representing week 4)**.** All metabolite abundance values are normalized to the internal standard. This model was explained by 9 components with total variance = 0.904 and prediction power = 0.548. (**B**) Score plot of PC1 vs. PC2, complete separation of week 0 from week 4 samples. (**C**) Loading plot for PC1 and PC2 showing the major metabolites (trimethylsilylated) contributing to sample clustering. (**D**) Hierarchical cluster analysis (HCA) of GC dataset shows two main clusters. **U** = untreated; **P** = pomegranate; **B** = benzo[a]pyrene; **PB** = pomegranate + benzo[a]pyrene.

One hundred and two metabolites (Table S1 and Supplementary Data File S2), out of ~530 mass spectra (MS) detected peaks, were annotated from all examined samples and classified into 13 classes (Figure 8A). The levels of the detected metabolites were normalized to that of spiked internal standard xylitol.

GC-MS analysis indicated that the rat fecal metabolome switched from a sugar-dominated to a fatty-acid-dominated profile (Figure 8A and Figure S8). On average, sugars represented the major class in samples from week 0, constituting 54% of the total detected metabolites, followed by acids (13%), whereas fatty acids represented the major class in samples from week 4, constituting 26% of the total metabolites, followed by sugars (22%). Other minor identified classes included sterols (0.44% in week 0 and 0.9% in week 4), esters (0.47% in week 0 and 0.64% in week 4), and aliphatic hydrocarbons (0.5% in week 0 and 1.15% in week 4 (Table S2)).

A general increase in fatty acids alongside their esters was observed in week 4 samples by 18.1% (1.18-fold), and this increase was mostly manifested by 1-monooleoylglycerol (6.9-fold), followed by 9-octadecenoic acid -2-propyl ester (5.2-fold) and oleic acid (2.4-fold). An increase in organic acids by 6.2% (1.06-fold) was largely contributed by 3-hydroxyphenylpropionic acid (2.8-fold), followed by 5-hydroxyindoleacetic acid (2.5-fold) and 3-hydroxyphenylacetic acid (2.1-fold). Aromatic hydrocarbons also increased by 45.3% (1.45-fold), with the largest increase represented by p-xylene (1.5-fold). Finally, sugar alcohols increased by 30.8% (1.3-fold), mostly manifested by an increase in maltitol (14.3-fold), pinitol (1.7-fold), and myoinositol (1.4-fold).

Reciprocally, a general decrease in sugar levels by 76.4% (0.23-fold) was observed in week 4 samples. This remarkable decrease was contributed by glucose (0.16-fold) and arabinose (0.17-fold). The next most substantial decrease was in amino acid levels (42.8% drop or 0.57-fold) and was reflected in a decrease in levels of isoleucine (0.18-fold), serine (0.22-fold), and leucine (0.6-fold). Finally, alcohols decreased by 47.4% (0.53-fold).

Of note, all detected alcohols (e.g., 1,3-propanediol, propylene glycol, and ethylene glycol) increased by the end of week 4, except 1,4-butanediol, which decreased by 76.5% (0.23-fold), driving the entire class down. Additionally, β-alanine, glycine, L-alanine, and L-threonine amino acids increased in week 4 samples by 1.5, 1.24, 1.2, and 1.08-fold, respectively.

As for the major changes in the levels of metabolite classes each of week 4 treatment groups compared to the untreated group two classes seemed to diminish in all groups: (i) acids diminished by 0.29, 0.26, and 0.31-fold in the Pom, BaP, and PomBaP groups, respectively; and (ii) aromatic hydrocarbons diminished by 0.42, 0.47, and 0.53-fold in the Pom, BaP, and PomBaP groups, respectively.

The following classes were enriched in all treatment groups, compared to the untreated group (Table S1): (i) alcohols increased by 1.7, 6.6, and 1.4-fold in the Pom, BaP, and PomBaP groups, respectively; (ii) amino acids increased by 2.2, 1.3, and 2.8-fold in the Pom, BaP, and PomBaP groups, respectively; (iii) phenolic acids increased by 2.5, 2.1, and 1.8-fold in the Pom, BaP, and PomBaP groups, respectively; (iv) sugars increased by 1.6, 1.4, and 1.1-fold in the Pom, BaP, and PomBaP groups, respectively; and (v) sugar alcohols increased by 3.6, 2.3, and 8.8-fold in the Pom, BaP, and PomBaP groups, respectively. Fatty acids were enriched in the Pom and BaP groups (by 1.15 and 1.16-fold, respectively) but diminished in the PomBaP one (by 0.5-fold).

### 2.8. Unsupervised and Supervised Multivariate Data Analysis of GC-MS Datasets

To further classify the treatment groups, we used PCA and orthogonal projection least-squares discriminant analysis (OPLS-DA) to determine the impact of different treatments on rat metabolome profiles in an untargeted manner.

#### 2.8.1. Clustering of the Entire Dataset into Two Separate Clusters by PCA and OPLS-DA 

We started by applying PCA to the entire dataset (all samples and all metabolites included) to investigate potential clustering patterns and determine possible metabolome heterogeneity among all examined samples. Nine components accounted for 90.4% of the total variance (R^2^), with the first two components (PC1/PC2) accounting for 65% of the variance (score plot in Figure 8C). All samples could be segregated into two distinct clusters, distributed along PC1. As observed with the microbiome data, these two major clusters were clearly set apart based upon the age/temporal factor (week 0 vs. week 4, Figure 8B–D).

To determine the key metabolites responsible for the sample segregation, we applied loading plots to the PCA models. Among detected metabolites, sugars (glucose, arabinose, and glycerol), fatty acid esters (1-monooleoylglycerol), and aromatic hydrocarbons (p-xylene) accounted for most of the observed variability along PC1 (Figure 8C). Metabolites with extreme positive p[1] values were more abundant in week 4 samples and contributed to their separation, whereas metabolites with extreme negative p[1] values were more abundant in week 0 samples and led to their separation to the left of the graph. 

Considering that sugars accounted the most for the observed separation between week 0 and week 4 samples (Figure 8C), we repeated the PCA analysis after the exclusion of all sugars. The same segregation pattern was largely retained in this analysis, in which PC1/PC2 explained 55% of the variance (Figure S9). However, while week 0 samples were clearly clustered, week 4 samples were intriguingly divided into a major cluster, which included most samples, and a minor one, which was enriched in samples from the Pom-treated group (Figure S9A). Such a pattern was confirmed by hierarchical clustering analysis, which separated the non-sugar metabolome profiles into three clades, including a week 4 Pom-enriched clade (Figure S9C).

After the exclusion of sugars, the corresponding loading plots uncovered another set of metabolites that contributed to the variability along PC1 and, hence, segregation. This set of metabolites included acids (hydroxybutyric acid, hydroxybenzoic acid), the amino acids valine, the alcohol 1,4-butanediol, and the previously detected fatty acid esters (1-monooleoylglycerol) and aromatic hydrocarbons (p-xylene, Figure S9B).

In agreement with the PCA results, a supervised OPLS-DA model, applied to the entire dataset and explaining 48% of the total variance, confirmed the age-based segregation of the samples (Figure S10A). Likewise, as sugars constituted the major variable separating week 0 from week 4 samples (Figure 8C), we re-applied the same model after the exclusion of sugars to uncover other possible contributors to the observed age/time-based segregation pattern (Figure S10C).

For OPLS-DA models, an S-loading plot was used to visualize the covariance (p) and the correlation (*pcor*) between the variability-generating metabolites. Like with PCA, sugars (glucose, arabinose, and glycerol), the fatty acid ester 1-monooleoylglycerol, and the aromatic hydrocarbon p-xylene accounted for most of the observed variability along PC1 (Figure S10B). Yet, unlike PCA, hydroxybutyric acid, the fatty acid myristic acid, and the sugar alcohol maltitol added to observed variability along PC1 (Figure S10B).

After the exclusion of sugars from the OPLS-DA model, like with PCA, 1-monooleoylglycerol, hydroxybutyric acid, p-xylene, 1,4-butanediol, and the amino acid valine accounted for most of the observed variability along PC1. Yet, unlike PCA, fatty acids and fatty acid esters (1-monopalmitin and myristic acid), 3-hydroxyphenylpropionic acid, and the amino acid L-leucine added to the observed variability along PC1 (Figure S10D).

Of note, because of the complexity of data and multiple compared variables, we conducted week 0 vs. week 4 analysis in subsets based on the assigned treatment groups (Figures S11 and S12). An OPLS-DA model and S-Plot that were applied to each of week 0–week 4 pairs singled out sugars as the dominant class of discriminatory metabolites (Figure S11). After the exclusion of sugars, several metabolites seemed to contribute to the distinction of week 0 samples to include succinic acid (in the untreated and Pom groups); linoleic acid (in the BaP and PomBaP groups); isoleucine (enriched in the Pom group); and uracil, palmitic acid, and 9-octadecenoic acid -2-propyl ester (enriched in the PomBaP group). Meanwhile, lactic acid was associated with week 4 samples of the PomBaP group (Figure S12).

#### 2.8.2. Lack of Segregation between Week 0 Sample Groups

PCA was also applied to the normalized metabolome profiles from week 0 samples to investigate any metabolomic heterogeneity among these samples from control rats before any treatment was applied. As expected, the entire pool was not segregated into any subclusters (Figure S13). A loading plot for this analysis confirmed the absence of major discriminatory metabolites among week 0 samples, except for some individual metabolites marking the natural biological variability among rats. These metabolites included lactic acid, as well as glucose/glucopyranose and p-xylene, which were associated with a couple of outliers (Figure S13B).

### 2.9. Alterations in Fecal Metabolomes in Response to Daily Ingestion of Pom, BaP, and PomBaP

**Figure 9:**
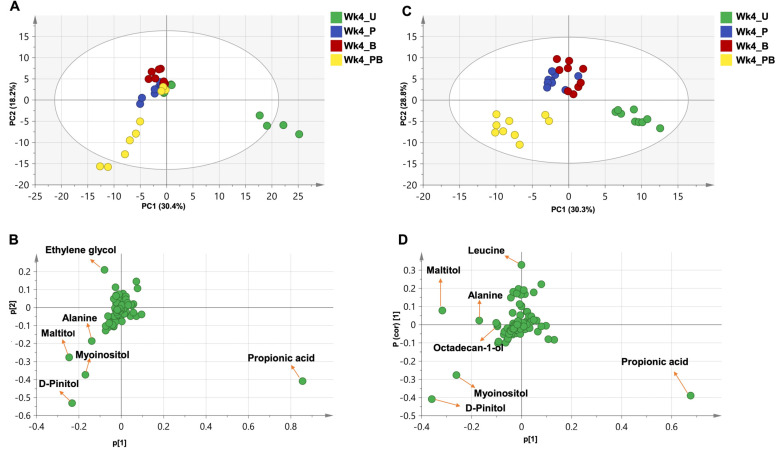
PCA and OPLS analysis for week 4 treatment groups normalized to week 0 time point. (**A**,**B**) PCA for week 4 treatment groups. This model was explained by five components with total variance = 0.775 and prediction power = 0.103. (**A**) Score plot of PC1 vs. PC2, showing a separation between two clusters (one for the untreated group and one for the rest of the groups). (**B**) Loading plot for PC1 and PC2 showing the major metabolites contributing to sample clustering. (**C**,**D**) OPLS for week 4 treatment groups. This model was explained by three components with total variance = 0.888 and prediction power = 0.745. (**C**) Score plot of PC1 vs. PC2 showing three clusters. (**D**) S-Plot for PC1 and PC2 showing the major metabolites (trimethylsilylated) contributing to sample clustering. **U** = untreated; **P** = pomegranate; **B** = benzo[a]pyrene; **PB** = pomegranate + benzo[a]pyrene.

Like in analyzing microbiome data, it was important to determine treatment-specific factors that are independent from age/temporal changes. Thus, metabolite level values from different treatment groups of week 4 were normalized to their corresponding values in week 0 samples. When PCA, as an unsupervised model, was applied to this double-normalized dataset of week 4 samples, 77.5% of the variance was explained by five components, with PC1/PC2 accounting for 48.6% of the total variance. The samples could be separated into two clusters distributed along PC1, a cluster representing the untreated group and another cluster representing the rest of the treatment groups. (Figure 9A). Sugar alcohols (maltitol, myoinositol, pinitol), the amino acid alanine, and propionic acid accounted for this separation (Figure 9B).

As a supervised model, OPLS-DA accounted for 59.1% of the total variance, and the examined dataset could also be separated into three clusters along PC1, which were more distinct than in the PCA, as expected from supervised modeling as such. The clusters included an untreated group cluster, a PomBaP group cluster, and a cluster combining the Pom and BaP groups (Figure 9C). The same metabolites (maltitol, myoinositol, pinitol, alanine, and propionic acid) accounted for this separation, in addition to leucine and octadecan-1-ol (Figure 9D).

**Figure 10:**
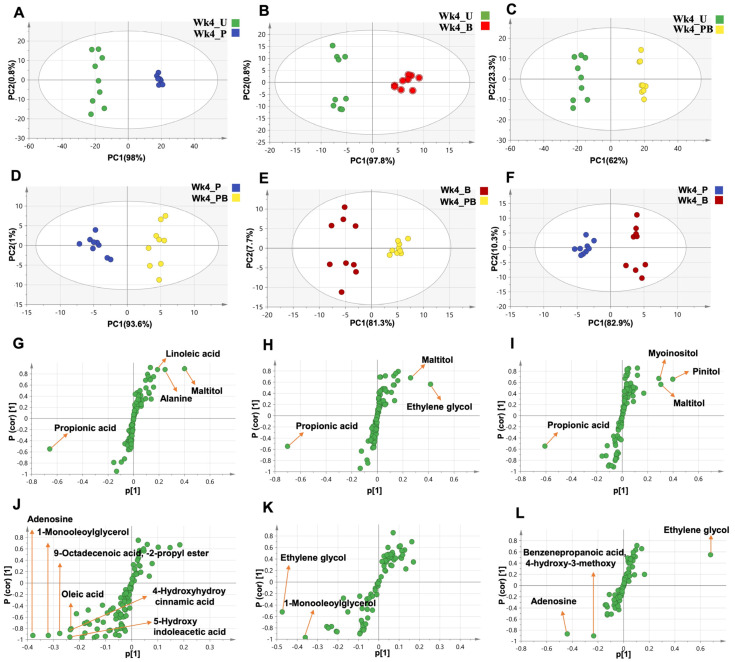
OPLS model for week 4 treatment groups normalized to their corresponding week 0 groups. (**A**) Untreated vs. Pom; (**B**) untreated vs. BaP; (**C**) untreated vs. PomBaP; (**D**) Pom vs. PomBaP; (**E**) BaP vs. PomBaP; (**F**) Pom vs. BaP. (**G**–**L**) S-Loading plots showing the major metabolites contributing for the variability between the untreated and Pom groups (**G**), untreated vs. BaP groups (**H**), untreated vs. PomBaP groups (**I**), Pom vs. PomBaP groups (**J**), BaP vs. PomBaP groups (**K**), and Pom vs. BaP groups (**L**). All normalized metabolite abundance values were also normalized to the internal standard. **U** = untreated; **P** = pomegranate; **B** = benzo[a]pyrene; **PB** = pomegranate + benzo[a]pyrene.

To unveil other metabolomic differences between week 4 treatment groups, we implemented a set of OPLS-DA models for pairwise comparison between these groups (Figure 10). The model comparing the untreated vs. Pom groups (R^2^ = 98.8, Figure 10A) indicated propionic acid, linoleic acid, maltitol, and alanine as the principal contributors to the observed variability (Figure 10G). The model comparing the untreated vs. BaP groups (R^2^ = 98.6%, Figure 10B) suggested propionic acid, maltitol, and ethylene glycol as the major variables (Figure 10H). As for the untreated vs. PomBaP model (R^2^ = 96.5%, Figure 10C), it suggested propionic acid, maltitol, myoinositol, and pinitol as the most variable metabolites along PC1 (Figure 10I).

Likewise, OPLS-DA models were applied to the datasets from the Pom and PomBaP groups (R^2^ = 94.6%, Figure 10D), the BaP and PomBaP groups (R^2^ = 89%, Figure 10E), and finally the Pom and BaP groups (R^2^ = 93.2%, Figure 10F). 

In these models, 1-monooleoylglycerol, 9-octadecenoic acid -2-propyl ester, 4-hydroxyhydrocinnamic acid, 5-hydroxyindoleacetic acid, oleic acid, and adenosine accounted for most of the variability between the Pom and PomBaP groups (Figure 10J); 1-monooleoylglycerol and ethylene glycol accounted for most of the variability between the BaP and PomBaP groups (Figure 10K); and adenosine, ethylene glycol, benzenepropanoic acid, and 4-hydroxy-3-methoxy were the most variable metabolites along S-Plot PC1 between the Pom and BaP groups (Figure 10L).

### 2.10. Microbiome–Metabolome Correlations

**Figure 11:**
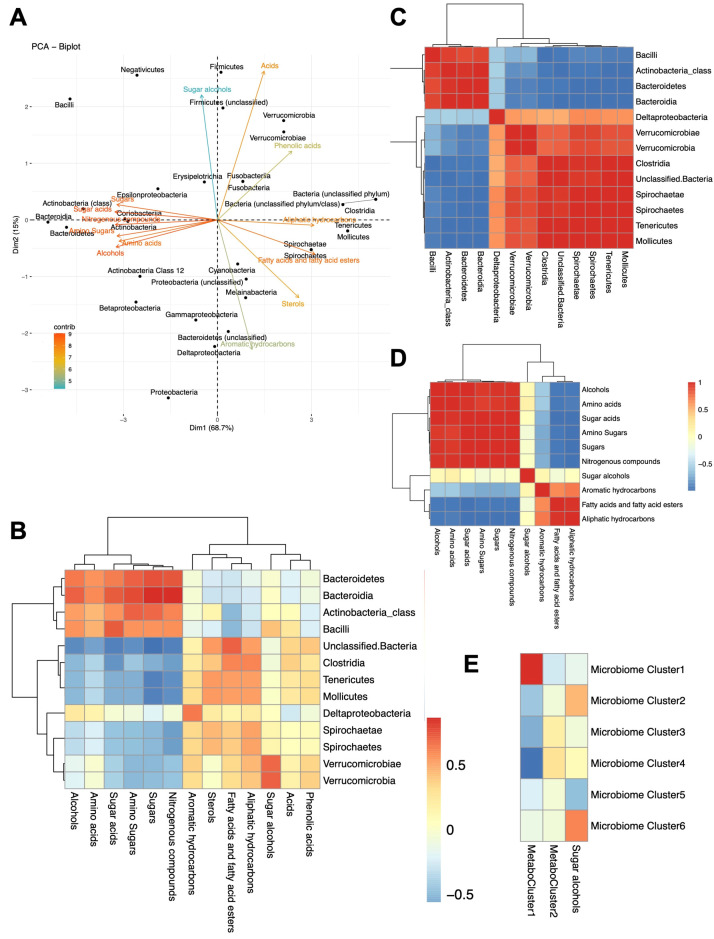
Microbiome–metabolome correlations. (**A**) PCA analysis of microbiome–metabolome correlations at the microbial phylum/class level vs. metabolite classes. Arrows show main metabolite classes responsible for microbial taxa segregation. (**B**) Heatmap depicting the Spearman rank correlation coefficients (*r_s_*) between microbiome phyla/classes and metabolite class pairs. (**C**) A correlation matrix of microbiome phyla classes according to their correlations with metabolites. (**D**) A correlation matrix of metabolite classes based on their correlation with microbial phyla/classes. (**E**) Correlations between microbiome clusters and metabolome clusters (metabotypes) deduced from Figure 12. For all correlations, only pairs with at least one coefficient (*r_s_*) > 0.5 or <–0.5 (for positive and negative correlations, respectively) are shown.

Having separately analyzed compositional and metabolic variations in the fecal microbiota, we investigated the potential metabolome–microbiome profile correlation(s) of all 24 samples, regardless of sampling time or treatment group (Figure 11 and Figure S14). In other terms, we determined which metabolites are more frequently associated with which microbial taxa in a statistically significant manner (with a Spearman correlation coefficient, *r_s_*, cutoff of ±0.5, *p* value < 0.05, and a taxon’s relative abundance threshold of 0.05% in at least one sample—Supplementary Data File S3). Overall, we found several amino acids to be more often correlated with Bacteroidetes (especially members of Bacteroidales S24-7 family, with *r_s_* = 0.783), while amino sugars were correlated with class Bacilli of the Firmicutes phylum (mainly represented by genus *Lactobacillus*, *r_s_* = 0.547) and genus *Anaerovibrio* of class Negativicutes (*r_s_* = 0.583). On the other hand, sugar acids were negatively correlated with phylum Firmicutes (mostly contributed by genus *Veillonella* and class Clostridia, with *r_s_* = −0.557 and −0.715, respectively (Figure 11A,B and Figure S14)). As for the major class ‘sugars’, they were positively correlated with Bacteroidetes (*r_s_* = 0.643) in general, especially class Bacteroidia (*r_s_* = 0.742) and family Bacteroidales S24-7 (*r_s_* = 0.814), in addition to some members of phylum Firmicutes, especially *Anaerovibrio* (*r_s_* = 0.730) and *Lactobacillus* (*r_s_* = 0.543); conversely, sugars were negatively correlated with *Family XIII* (*r_s_* = −0.773), and genera *Anaerovorax* and *Ruminococcaceae UCG-014* (*r_s_* = −0.808 and −0.807, respectively).

Dominant members of phylum Firmicutes were divided into two contrasting patterns in how they correlate with metabolite levels: Most members of class Bacilli, e.g., family *Lactobacillaceae* and genus *Lactobacillus* but not family *Streptococcaceae*, had a positive correlation with amino acids, sugars, alcohols, and sugar acids (*r_s_* = 0.428, 0.543, 0.525, and 0.622, respectively), but a negative correlation with fatty acids and aliphatic hydrocarbons (*r_s_* = −0.677 and −0.394, respectively); by contrast, class Clostridia, including families *Ruminococcaceae*, *Peptostreptococcaceae*, and *Family XIII*, had metabolite correlations that are more similar to class Mollicutes of the phylum Tenericutes, which tended to correlate with sterols, fatty acids and fatty acid esters, and aliphatic hydrocarbons (Figure 11).

Some metabolites were specifically correlated with a small number of taxa, e.g., octadecenoic acid, its ester, and eicosanoic acid were correlated (*r_s_* = 0.616, 0.612, 0.623, respectively) with the rare phylum Fusobacteria. Lactic acid, expectedly, was highly correlated with phylum Firmicutes, which includes the well-known lactic acid bacteria. Glycolic acid and glycine clustered and were positively correlated with Verrucomicrobia (Figure S14), while pinitol, which also partly clustered with glycolic acid and glycine, was positively correlated with Verrucomicrobia, Erysipelotrichia, and Firmicutes in general (*r_s_* = 0.44, 0.608, and 0.481, respectively). Fatty acids and fatty acid esters, especially the propyl esters of stearic acid and 9-Octadecenoic acid, were positively correlated with phylum Tenericutes and its class Mollicutes, as well as Proteobacteria and Fusobacteria, but were interestingly most correlated with unclassified bacteria (Figure 11 and Figure S14). A full list of correlation coefficients is provided in Supplementary Data File S3.

In addition to metabolites determined by GC-MS, the measured fecal β-glucuronidase activity was positively correlated with the relative abundance of class Clostridia, especially *Family XIII*, genera *Veillonella* and *Holdmanella*, and family *Ruminococcaceae* (with correlation coefficients *r_s_* = 0.56, 0.62, 0.65, 0.66, and 0.57, respectively) and negatively correlated with Bacteroidetes (*r_s_* = −0.56) and genus *Anaerovibrio* of the Negativicutes (*r_s_* = −0.53).

**Figure 12:**
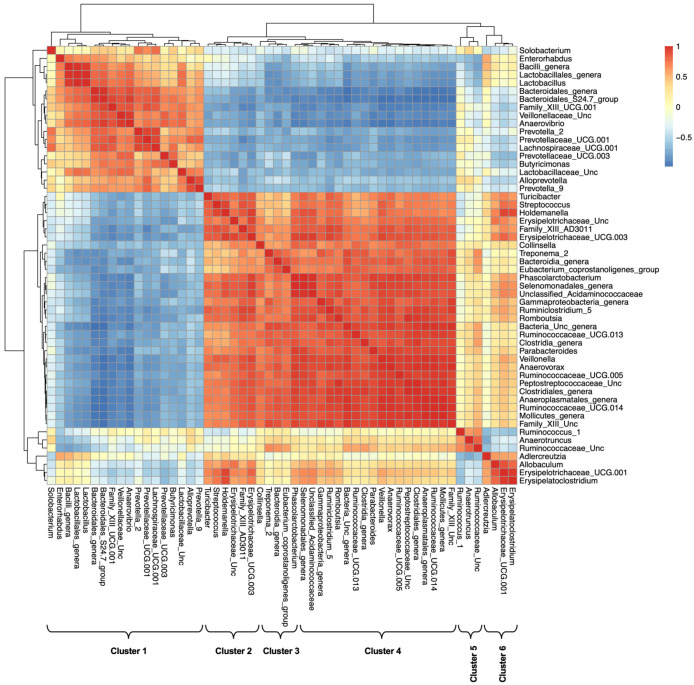
Correlation matrix of microbial genera based on their correlation with metabolites. Six clusters that represent similar patterns are indicated. Unc = unclassified. The heatmap is colored based on Spearman correlation coefficients *r_s_*.

Of interest, when we examined the correlation among bacterial taxa based on their co-abundance with fecal metabolites (Figure 11C and Figure S15), two major clusters of bacterial taxa were distinguishable (Figure 11C): one cluster includes Bacilli, Bacteroidetes, and Negativicutes, while the other includes Clostridia and most Firmicutes, Verrucomicrobia, and Tenericutes. At the family and genus levels, these two clusters could be further divided into six (Figure 12 and Table 1).

Likewise, metabolic classes were split into two major clusters: one included amino acids, sugars, amino sugars, and alcohols, while the other included fatty acids and their esters, as well as aromatic and aliphatic hydrocarbons. Sugar alcohols stood out uniquely, with correlation patterns that spanned the two clusters (Figure 11D,E). Correlation patterns of individual metabolites are provided in the Supplementary Materials (Figure S16).

## 3. Discussion

Humans and animals are continually and increasingly exposed to environmental toxicants, many of which are potential carcinogens. Among these toxicants are polycyclic aromatic hydrocarbons (PAHs), which are common air pollutants and are enriched in grilled, fried, and processed foods [21,22,23]. A prototypic representative of these toxicants is benzo[a]pyrene (BaP) [24,25], which is one of the two main chemicals studied in this work.

As a reaction to the alarming levels of pollutants, especially carcinogens, in food and the environment, several calls have been made to return ‘back to nature’ by seeking alternatives to procarcinogenic foods and toxicants or at least protection from their harmful effects. Among the most popular natural products are polyphenols, and their representative plant source in this study is pomegranate juice.

Plenty of polyphenol-rich foods have been examined for anticarcinogenic properties against BaP-induced colon pathologies in vivo. These food components include resveratrol [66], olive oil [67], silver *Moringa oleifera* leaves [68], and curcumin [69]. However, little experimental evidence is available in the literature on the role of pomegranate as a chemoprotective agent in the presence of BaP.

Great attention has lately been paid to the role of the human microbiota, the assemblage of microorganisms residing in different living and non-living environments, and among the key described roles of the microbiota is its role in modulating xenobiotics (toxicomicrobiomics), sometimes increasing their toxic effects and sometimes detoxifying them [70,71].

While experimentally investigating the causality of human–microbiome interactions is usually neither ethical nor feasible, animal models are powerful tools for establishing causality, even if not fully representative of the human host. They allow the use of proper controls and allow longitudinal studies to be performed in a relatively short time, without the obstacles and limitations associated with human studies.

Here, we used a well-controlled rat model to investigate the changes in the intestinal tissue, microbiome, and metabolome over time, in response to the ingestion of two chemicals with procarcinogenic and anticarcinogenic potential.

The major phenotypic changes that we observed in the experimental model are histopathological, while animal weights remained unchanged. In addition, we measured the glucuronidase activity in feces as a marker for xenobiotic-modifying potential, and we ensured that rats were randomized for this activity, which significantly increased after the four-week experiment, but that increase was similar across all treatment groups, and no significant increase was observed in a particular group.

### 3.1. Histopathological Alterations in Rat Colons

Our histopathological examination indicated significant observable inflammation and damage to the colonic tissues of rats after a daily ingestion of BaP for 28 days, which was not detected at all in Pom-fed rats and was partly restored by the addition of Pom to BaP (Figure 1 and Figure 2). The damage was manifested by signs of inflammation, mitosis, dysplasia, and a significant decrease in reactive mucin.

Mucins are produced and secreted by goblet cells and represent a source of nutrition to beneficial commensals while protecting the colon epithelium from pathogenic bacteria by forming a covering mucus layer [72]. Pomegranate’s positive impact on mucin is well documented. For example, supplementation of the basal diet administered to male Sprague–Dawley rats with 10% pomegranate powder significantly increased the mucin content in tissues that had aspirin-induced ulcers by 52.7%, compared to untreated animals [73]. Pomegranate extract significantly decreased the number of mucin-depleted foci (MDF) in the colons of rats fed a carcinogenic dark cooked meat with nitrite, oxidized (DCNO)-based diet [74].

When a pomegranate mesocarp decoction was administered, at a dose of 50 mg/kg/day for 6 weeks, to a rat model that spontaneously developed colon adenomas, it significantly reduced the number of MDF [75]. In a study on a chronic trinitrobenzene sulfonic acid (TNBS)-induced colitis Wistar rat model, dietary supplementation of pomegranate extract to the rats for four weeks attenuated the morphological signs of cell damage in the colonic mucosa and reversed mucin depletion in the Alcian-blue-positive goblet cells [76]. In another TNBS-induced colitis model in male Wistar rats, ingestion of ellagic acid (10–20 mg/kg)—the main constituent of pomegranate—by oral gavage reduced the inflammatory cell count and morphological signs of cell damage. It also caused the colon epithelium to remain intact and increased the amount of mucin stained by Alcian blue in colon mucosa [77].

### 3.2. Fecal Microbiome and Metabolome Alterations

#### 3.2.1. Temporal/Age Effects

The most striking finding from microbiome and metabolome analyses is that week 4 vs. week 0 differences are the major factor influencing both microbial taxonomic composition and metabolome profiles. Given that the samples were collected in a similar way, aliquoted, and immediately frozen, and that all DNA extraction, amplicon sequencing, and metabolome profiling by GC-MS were performed simultaneously and with all samples randomized at every analysis step, we believe that this strong temporal factor reflects real biological effects rather than technical variability.

This factor could be due to natural aging or could simply reflect stochastic, temporal variation of the microbiota [78]. The rats were already out of adolescence [79] and were thus neither in a transitional developmental stage (as confirmed by their weights that did not significantly increase during the four weeks of the experiment, Figure S1). They cannot be described to be aged either, because the experiment ended when the rats were 12–13 weeks old [79,80]; however, four weeks in an adult rat’s lifespan are approximately equivalent to 2.5 years in human life [80,81].

Importantly, although rats can live up to 3.5 years [80], we opted to use young adult rats (7–12 weeks from the time they were obtained until the end of the experiment) to rule out any increased cancer incidence due to the age factor [82].

Both age and temporal variations have been previously described to significantly affect microbiome composition in humans and other mammals [83,84]. Additionally, there is no specific reason to assume that these differences are related to dietary or laboratory environmental factors because the rats were acclimated to the laboratory environment and to a standard diet that was unchanged throughout the experimental period.

Among the key observed age/temporal differences is a general increase in alpha diversity (both richness and evenness, Figure S4), reflected in an increase in the Shannon diversity index as well, which takes both diversity richness and evenness into account (Figure S4). This diversity increase was curiously accompanied by two opposing observations: one is the decrease in relative abundance of three major taxa (Figure 6B): (i) Bacteroidetes, with the two major families of Bacteroidales S24-7 group and *Prevotellaceae*, (ii) *Veillonellaceae*, and (iii) *Anaerovibrio*; the other is the relative increase in unclassified bacteria (13.3% week 0 vs. 19.8% week 4, paired Wilcoxon test *p* value < 0.05). However, the drop in relative abundance of the three major groups mentioned above was counterbalanced by an increase in the relative abundance of a dozen other taxa, including Tenericutes, Clostridia, and *Acidaminococcaceae* (Figure 6A). Family *Veillonellaceae* was split in terms of its temporal variations: The abundance of genus *Veillonella* increased (Figure 6A), while that of genus *Anaerovibrio* decreased, influencing the entire family’s relative abundance (Figure 6B).

The impact of age differences, even if small, is not unprecedented. Zhang et al. [85] found significant aging-associated changes in gut microbiota profiles and serum metabolites: Phylum Firmicutes was positively correlated, while Bacteroidetes, Proteobacteria, and Actinobacteria were negatively correlated with age [85]. In another study, age was reported as the major factor, significantly altering the fecal microbiota composition of Zucker rats, outweighing genetic factors [86]. The Firmicutes-to-Bacteroidetes ratio and the relative abundance of families *Bacteroidaceae* and *Peptostreptococcaceae* decreased, while the relative abundance of families *Ruminococcaceae* and *Bifidobacteriaceae* increased with age. The same study reported the cage environment as a key factor affecting microbiome variation [86]. The differences between some reported age effects (e.g., *Peptostreptococcaceae*) and those that we report should not be alarming as some taxa may be naturally fluctuating, which is not necessarily related to biological aging.

Another relevant example of age effects is that antibiotic-induced microbiota depletion prior to repeat mild traumatic brain injury (RmTBI) reportedly led to chronic changes in microbiome composition and diversity, as well as metabolome profile, in both adolescent and adult Sprague–Dawley rats, with adolescents exhibiting greater changes [87].

Animal age also has a major impact on metabolomic profiles. Both multivariate data analyses (PCA and OPLS-DA) indicated a general decrease in sugar levels (mainly glucose, arabinose, and glycerol) in week 4 samples. Valine, L-leucine, and 1,4 butanediol levels also decreased in week 4 samples, and their delineation was made possible when all sugars, the dominant metabolites, were excluded from analysis models.

Reciprocally, p-xylene and the fatty acid 1-monooleoylglycerol levels increased in week 4 samples as detected by all models (PCA and OPLS-DA, sugars included and excluded). However, the exclusion of sugars allowed the detection of more changes, as short chain fatty acids (SCFAs) (i.e., hydroxybutyric acid, aromatic acids i.e., hydroxybenzoic acid, 3-hydroxyphenylpropionic acid) and fatty acids (i.e., myristic acid and 1-monopalmitin) increased in week 4 samples. Excluding a dominant factor from the analysis is an efficient technical maneuver to overcome its masking effect, especially in similar cases with big datasets and multiple time points/analysis groups.

Song et al. [88] found a significant difference in fecal fatty acid levels between CRC patients and healthy controls. However, the authors also noted a significant difference between the mean age of the two groups, which suggests that fatty acid metabolome might have been affected by age [88].

In general, we resorted to using PCA and OPLS-DA as dimensionality reduction analysis tools because of the complexity of the acquired data (72 chromatograms of 24 samples in triplicates, with 530 detected metabolites, including 102 that were fully identified and classified).

Another way to sort out the signal from noise, to resolve dominant factors (e.g., age here), and to detect smaller true-positive changes in these large datasets is subset analysis. When we conducted week 0 vs. week 4 analysis in subsets based on the assigned treatment groups, we detected an increase in 1-monooleoylglycerol with all assigned treatments (i.e., it is an age-dependent change and has no direct relation to the type of food/treatment ingested by the rats, Figure S12), while p-xylene only increased in the week 4 Pom and week 4 PomBaP groups. The increase in 3-hydroxyphenylpropionic acid level was observed in the week 4 Pom and week4 BaP groups, whereas the increase in myristic acid was observed in the week 4 untreated and week 4 PomBaP groups. In the literature, p-xylene was found among the detected phenolic metabolites in the feces of the pomegranate-peel-extract-treated group of beef calves [89].

As for the major changes in metabolite levels in the week 4 treatment groups, another technical maneuver was to apply a set of OPLS-DA models for pairwise comparison between the double-normalized datasets of the week 4 groups. Compared to the untreated group, all groups had higher levels of maltitol, the Pom group had higher levels of linoleic acid and alanine, while the BaP group had higher levels of ethylene glycol (Figure 10). Propionic acid appeared to diminish in all groups in comparison to the untreated group, an apparent decrease that possibly arose inadvertently as the propionic acid level was unusually lower in week 0 animals that were assigned to the untreated group than other animals, which led to a higher week 4-to-week 0 ratio (Supplementary Data File S1).

Comparing the BaP or Pom groups head-to-head with the PomBaP (double-fed) group revealed interesting differentially abundant metabolites: Oleic acid, adenosine, 4-hydroxyhydrocinnamic acid, 5-hydroxyindoleacetic acid, and 9-octadecenoic acid-2-propyl ester had higher levels in the Pom group than the PomBaP group, while ethylene glycol was enriched in the BaP group, in comparison with PomBap. Ethylene glycol was also enriched in the BaP group compared to the Pom group (Figure 10).

Both 4-hydroxyhydrocinnamic acid and 3-hydroxyphenylpropionic acid are low-molecular-weight phenolic acids that have been described to result from the gut microbiota metabolism of polyphenols [90,91]; thus, their association with the Pom group is reasonable and presents potential markers for pomegranate consumption in stool samples. Similarly, 5-hydroxyindoleacetic acid, which is a tryptophan metabolite, was found to significantly increase after dietary supplementation of magnolol polyphenols to a dextran sulfate sodium (DSS)-induced ulcerative colitis mouse model [92], and supplementation of the basal diet of male Ross-308 chicks with a mixture of pomegranate and onion extracts significantly increased oleic acid level [93].

#### 3.2.2. Treatment Effects

Despite the quite strong signal contributed by the temporal factor, the paired design of the study allowed measuring changes that are associated with the experimental treatment with pomegranate, BaP, or both. For proper minimization of the temporal effect, a week 4-to-week 0 abundance ratio was calculated for different microbial taxa in every week 4 sample. This normalization was only technically possible for samples that had non-zero abundance value in every week 0 cage because division by zero is not possible. However, this data filtration step had a minor effect on the results because taxa with zero abundance values in week 0 samples had limited abundance in week 4 samples, in most cases (Supplementary Data File S1).

As for the major differences between treatment groups of week 4, unclassified genera of *Prevotellaceae* were significantly more abundant in the Pom-fed rats, while *Allobaculum* (family *Erysipelotrichaceae*) was less abundant in the same group. Unclassified genera of *Veillonelleacae* were significantly more abundant in the BaP-fed rats, while *Lactobacillaceae* (mainly *Lactobacillus*), non-*Lactobacillaceae* members of class Bacilli (Bacilli of unclassified order, family, or genus), and genus *Allobaculum* were significantly more abundant in PomBaP-fed rats. 

Family *Erysipelotrichaceae* (including genus *Allobaculum*) has been associated with inflammatory gastrointestinal disorders and higher levels of proinflammatory tumor necrosis factor [94,95]. Compared to the healthy controls, an increased abundance of *Erysipelotrichaceae* was observed in colorectal cancer patients [96], and an increased abundance of genus *UCG-003* of family *Erysipelotrichaceae* was observed in patients with inflammatory bowel disease [97]. A significantly higher level of *Erysipelotrichaceae* was found in the tumor group of an animal model of 1,2-dimethylhydrazine-induced colon cancer [98]. Urolithin A (a gut-microbiota-mediated metabolite of pomegranate) could significantly decrease the enriched *Erysipelotrichaceae* in mice in which intestinal damage was induced with ionizing radiation [99].

An unusually high abundance of family *Veillonelleacae* was observed in patients with colon cancer [100,101] and Crohn’s disease [102]. Genus *Veillonella* was reportedly more abundant in patients with Hirschsprung’s-disease-associated enterocolitis [103], colon cancer [104], and ulcerative colitis and primary sclerosing cholangitis with concomitant ulcerative colitis [105] than in healthy controls (in all cases). The abundance of family *Prevotellaceae* (mainly *Prevotella*) significantly increased after four weeks of pomegranate extract ingestion by healthy volunteers [106]. Moreover, in an experimental autoimmune encephalomyelitis mouse model, family *Prevotellaceae* was enriched in pomegranate-extract-fed mice, compared to the control mice [107].

All the previous examples have major similarities with microbiome results reported here; however, to the best of our knowledge, no studies have reported microbiome/metabolome changes caused by a combination of BaP with pomegranate.

#### 3.2.3. Microbiome–Metabolome Correlations

In general, microbiome composition per se is not as biologically meaningful as metabolome variations because it is the metabolic activity of microorganisms that more profoundly affects the host biology—an observation that was highlighted over a century ago [108]—and because several bacteria from distant taxonomic groups may have similar metabolism, and vice versa [64]. However, attempts to integrate microbiome and metabolome variations across the samples provided strong correlations between subgroups of bacteria and subgroups of metabolites (Figure 11A,B and Figure S14).

Even more interestingly, these associations allowed better understanding of internal correlations within metabolite classes and microbial taxa. Two distinct clusters of bacterial taxa were identified, and these were further divided into six microbiome clusters (Table 1, Figure 11C and Figure S15), which were distinct in their co-abundance with metabolic classes (Figure 11E). Similarly, metabolites were split into ‘metabotypes’: a small cluster of fatty acids, as well as aliphatic and aromatic hydrocarbons vs. a larger cluster including sugars, amino acids, alcohols, and nitrogenous compounds. Interestingly, sugar alcohols were the most distinct in their microbial co-abundance patterns (Figure 11D and Figure S16).

In the literature, fecal amino acids, including glycine, were found to be positively correlated with families *Lactobacillaceae* (mainly *Lactobacillus*) and *Verrucomicrobiaceae* in Wistar rats [109]. Mao et al. [110] found that amino acids isobutyrate and isovalerate were positively correlated with *Anaerovibrio* in fecal samples collected from cows [110]. In a human study of cirrhotic patients, fatty acids (hexanoic acid and heptanoic acid) and the fatty alcohol hexadecanol were positively correlated with Tenericutes [111].

It is important not to interpret the microbiome–metabolome co-occurrence and co-abundance patterns, which we and others reported, as a direct result of microbial metabolic processes. The main reason for this precaution is that fecal metabolomics measures both host-derived and microbial metabolites. Additionally, it is impossible to sort out cause and effect in these cases, i.e., whether the abundance (or scarcity) of some metabolites allowed specific bacterial types to flourish or the abundance of certain microbes led to the production (or consumption) of specific metabolites. Taking microbial interactions into account even further complicates the interpretation of these associations. For example, some microbes may abundantly produce metabolites (e.g., fucose, rhamnose, or lactic acid) that are immediately consumed by other species, so the net outcome will seem as if those metabolites are diminished in the presence of the bacteria that is expected to produce them. Despite these analysis challenges, microbiome–metabolome positive and negative correlation data are extremely useful, and with the accumulation of such data from various studies, models for microbiome/metabolome biomarkers are expected to abound in the near future, leading to the proposal of rapid assays for predicting different health conditions affected by the microbiota.

### 3.3. Other Phenotypic Alterations

In addition to the major dependent variables measured in this multivariate study (i.e., histopathological changes, mucin levels, microbiome composition and diversity metrics, and metabolite levels), animal weight and β-glucuronidase activity were measured and used for randomization to minimize their confounding effect on experimental factors.

Overall, we observed no significant change in weight in any experimental group, or between the start and end of the experiment. Other studies reported similar results with either BaP or pomegranate. For example, no significant weight change was reported between the control and BaP-fed (dose = 150 µg/kg/day) rats after 30 days of ingestion [112]. The weights of Sprague–Dawley rats did not significantly differ between the control group and either of the low or high-dose pomegranate-peel-extract-fed rats after 12 weeks of extract ingestion [113].

β-glucuronidase is one of the most studied families of microbially derived enzymes involved in the breakdown and metabolism of glycoconjugates that reach the colon intact. It deconjugates hepatically glucuronidated metabolites, reversing phase II metabolism carried out on endo- and xenobiotics and reactivating their aglycone form (which can be excreted), undergoing further enterohepatic recirculation or further degradation and biotransformation by the gut microbiota (in case of polyphenols [114]), thus regulating the pharmacokinetics as well as the level of metabolites of both polyphenols (pomegranate) and xenobiotics (pro-carcinogenic BaP), and affecting their potential favorable and/or adverse effects on host health [115,116,117].

In general, β-glucuronidase activity was described in the four major gut-associated bacterial phyla: Bacteroidetes, Firmicutes, Proteobacteria, and Actinobacteria [118]; however, within each phylum, there is a wide pattern of presence/absence of these enzymes among families and genera. Haiser and Turnbaugh [119] highlighted major differences among Firmicutes and Bacteroidetes, based on genomic analysis [119]. In human feces, high β-glucuronidase activity was observed in isolates belonging to *Clostridium* spp. [120] and in *Roseburia* and *Faecalibacterium* (both belonging to class clostridia) [121]. Clostridia were among the positively correlated taxa with β-glucuronidase activity in this work, as detailed in the Results Section. 

A general, but non-group-specific, increase in fecal β-glucuronidase activity between week 0 and week 4 samples was observed in this study. Like with microbiome/metabolome age-related changes, it is hard to determine whether the changes are caused by the aging process or are stochastically affected by transient microbial restructuring. Reports in the literature are discrepant. For example, a comparison between adolescent and adult groups of C57BL/6 mice found no significant difference in enzyme activity in males but a significant reduction in activity in females [122]. Another study in a colon cancer BALB/c mouse model reported an increase in β-glucuronidase level with age in the control group [123]. In male Fischer rats, β-glucuronidase enzyme activity increased with age during the juvenile development stage [124] and in both the grain-based and meat-based diet groups [125], but it did not significantly change between three age groups: young (2–4 months), middle-aged (12–14 months), and old (22–24 months) [126].

### 3.4. Limitations of This Study

A study with such complexity and multiple interdependent variables can always be improved by a larger sample number and deeper or more comprehensive shotgun sequencing; however, for the purpose of defining key taxa and key metabolites while minimizing individual variations via the paired design (week 0 and week 4 sampling), this study achieved its goals. For 16S rRNA amplicon analysis, the depth of iSeq output is adequate for microbiome studies; however, the amplicon length (150-nucleotide read length, i.e., 300 nucleotides for a read pair) limits taxonomic resolution to the genus level, and no claims can be made about species distribution (even if the analysis pipelines may assign some species). Future studies should take advantage of the surging long-read sequencing technologies to have a higher resolution taxonomic assignment. In addition, we chose GC-MS to cover a wide range of microbial metabolites and compare across samples; however, detection of high-molecular-weight phenolics could not be achieved by GC-MS, for example. Future work will resort to both GC-MS and liquid chromatography mass spectrometry (LC-MS) for better profiling of biotransformed secondary metabolites of pomegranate.

An obvious limitation of any animal model is that laboratory animals do not exactly mimic the human system. In addition, the used Sprague–Dawley rats are neither isogenic to minimize the genetic variability nor wild to be diverse enough in their microbiota composition. However, starting with such experiments is pivotal to establishing working hypotheses that can be further validated in various genetic backgrounds, different animals, and eventually in humans.

In studies with this number of variables and groups, it is quite important to distinguish between statistical and biological significance. The number of samples per group and the presence of multiple treatment groups (untreated, Pom, BaP, PomBaP) are certainly technical hurdles against strong statistical significance; however, using this design is key for ruling out spurious, false-positive findings. For example, had we compared the different treatment groups at the end of the experiment without normalizing to week 0 values (to control for inter-individual and inter-cage variability), we would have ended up with a completely different set of results. 

Meanwhile, it is important to note that many true differences are subtle and can be biologically significant, even in observed numbers or levels that did not reach statistical significance by hypothesis-testing methods. A clear example is the appearance/disappearance of the *Prevotellaceae* family members in different analyses. *Prevotella* as a genus is one of the most abundant members in the gut microbiome of humans and several mammals. It is part of the abundant phylum Bacteroidetes, and its abundance is often inversely correlated with that of genus *Bacteroides* [127]; however, its reported biological effects are quite contradicting. Different members of family *Prevotellaceae* were identified throughout the analysis, but not all members or genera were found as statistically significantly different between the two time points or treatment groups. These variabilities can be resolved, in extended studies, by shotgun metagenomics or by long-read, full-length16S rRNA gene sequencing.

These types of limitations are common in multi-omics studies because data points are so numerous that statistical analyses often miss true-positive results. We consistently elected to err on the statistically conservative side to avoid false discovery, even at the expense of missing some true findings that remain to be resolved and confirmed in future studies.

### 3.5. Future Prospects

The findings of this study can be expanded in many directions. For example, testing whether pomegranate juice has a potential therapeutic effect can be achieved by repeating the experimental model with BaP administration for several weeks, followed by subsequent supplementation of pomegranate, after pathological damage has been confirmed (in a subset of animals) and BaP exposure has been discontinued.

In addition to testing the therapeutic potential of pomegranate juice, the same model can be expanded to test the intermediate and long-term effects of BaP, Pom, and PomBaP mixture in comparison to untreated animals, over several weeks or months.

Another way to expand the study is to use different fractions or components of pomegranate juice, or a combination thereof, to delineate the impact of each fraction and the potential synergistic activities of different bioactive components. Once pomegranate and its derivatives have been well tested, the model can test several other known or potential nutraceuticals.

Monitoring of the biotransformation of BaP and pomegranate in vivo via LC-MS or labeling is another direction for future studies.

From a translational perspective, the study is promising for the use of pomegranate juice, or its derivatives, as nutraceuticals. Two challenges need to be addressed in that case. First, the interactions of pomegranate with other drugs (reviewed in [128]) need to be taken into consideration. Second, the poor bioavailability of hydrolyzable tannins, which are key bioactive components of pomegranate juice, may be subject to formulation studies. Although the gut microbiota is pivotal in improving the bioavailability of hydrolyzable tannins, possible formulation alternatives include nanodelivery [129], micronization [130], and incorporation into bioactive film [131]. These alternatives may minimize microbiome-dependent intra- and inter-individual variation in chemoprotection outcome.

## 4. Materials and Methods

### 4.1. Study Design

To assess the interaction between the pro-carcinogenic BaP, anti-carcinogenic pomegranate juice, and the rat gut microbiota, we randomly allocated 48 male Sprague–Dawley rats to four treatment groups: (i) an untreated group, only fed a standard AIN76 diet; (ii) a pomegranate group, fed the standard diet + 50 mg gallic acid equivalent (GAE)/kg/day pomegranate juice; (iii) a BaP group, fed the standard diet + 150 μg/kg/day BaP, dissolved in peanut oil [132]; and (iv) a pomegranate + BaP group, fed the standard diet + 50 mg GAE/kg/day pomegranate + 150 μg/kg/day BaP (dissolved in peanut oil). Thus, the 48 rats were assigned to four treatment groups, each represented by three cages and each housed by four rats.

All doses were calculated according to the average weights of rats within a given cage. Rats in all treatment groups received peanut oil either as a solvent for BaP, in groups iii and iv, or by oral gavage, in groups i and ii.

### 4.2. Animals

Seven-week-old male Sprague–Dawley rats (*n* = 48) were obtained from the modern veterinary office, Cairo, Egypt, and were housed in plastic-bottom cages (four per cage) with hardwood chip bedding and acclimatized for 10 days before the experiment started. Rats were kept under standard conditions (temperature 25 ± 2 °C, relative humidity 60%) and a 12 h light/dark cycle. Food (AIN76 pellets) and water were given ad libitum.

The AIN76 diet was obtained from the Theodor Bilharz Research Institute, Cairo, Egypt. Pomegranate juice, peanut oil, and BaP were given to the rats daily by oral gavage for four weeks.

Animals were weighed weekly until the end of the experiment.

### 4.3. Chemicals

Benzo[a]pyrene (BaP, Seoul, South Korea, 98% pure), gallic acid, methanol (HPLC-grade), N-methyl-N-(trimethylsilyl)-trifluoroacetamide (MSTFA), and 4-Nitrophenyl β-D-glucuronide (PNPG) were obtained from Sigma-Aldrich (St. Louis, MO, USA). Folin–Ciocâlteu phenol reagent was obtained from Merck (Darmstadt, Germany), and peanut oil was obtained from Sekem company (Belbeis, Egypt). BaP was handled in accordance with NIH safety guidelines [133] and dissolved in peanut oil. Fresh solutions were prepared weekly and were stored at room temperature in the dark.

### 4.4. Preparation and Standardization of Pomegranate Juice

The entire edible part of the pomegranate fruit (*Punica granatum* L.), including the seeds (arils) and the attached sarcotestas, was thoroughly pressed in a Black & Decker Juice Extractor (Baltimore, MD, USA) to obtain concentrated pomegranate juice.

The juice was standardized by determination of total phenolics (TPC) according to the Folin–Ciocâlteu method [134]: A standard solution of gallic acid (10 mg/mL) was prepared by dissolving 1 g of gallic acid in 100 mL of methanol. A standard gallic acid curve was then constructed by preparing dilutions of 0.1, 0.2, 0.3, 0.4, 0.5, 0.6, 0.7, 0.8, 0.9, 1, 1.5 and 2 mg/mL in methanol from the standard solution (10 mg/mL) of gallic acid.

One hundred microliters of each dilution was mixed with 500 μL of distilled water and 100 μL of Folin–Ciocâlteu reagent. The mixture was then allowed to stand for 6 min. One milliliter of 7% sodium carbonate and 500 μL of distilled water were then added to the reaction mixture. After 90 min, the absorbance was spectrometrically measured at 760 nm [135]. The detailed characterization of individual phenolics in pomegranate juice by LC-MS has been previously described [136].

### 4.5. Sample Collection

At the start of the experiment, fecal samples were collected from each rat to evaluate basal glucuronidase enzyme activity. For sample collection, rats were placed in a clean, 70% alcohol-sterilized plastic box for no more than an hour. Stool was then aseptically collected by pre-sterilized forceps and placed in sterile 2 mL Eppendorf tubes. In some instances, sample collection required gently squeezing a rat’s rectal region to excise the fecal pellet directly into sterile 2 mL Eppendorf tubes. Fecal samples were kept at −80 °C until use.

During the experiment, fecal samples were collected weekly and pooled from each cage into sterile 15 mL falcon tubes and kept at −80 °C until use.

At the end of the experiment, rats were sacrificed by cervical dislocation, and their colons were retrieved as per the guidelines of Ruehl-Fehlert et al. [137] and preserved in 10% formalin.

### 4.6. Beta-Glucuronidase Assay

To determine the β-glucuronidase enzymatic activity in rat feces, we prepared fecalase from the feces according to a modified version [122] of a previously published method [138]. Briefly, 50–70 mg fecal pellet was suspended in a potassium phosphate buffer (0.01 M, pH 7.4) and homogenized for 30 min. The fecal suspension was then centrifuged at 2000 rpm for 10 min, and the resulting supernatant was centrifuged again at 15,000 rpm for 20 min. The supernatant (fecalase) was then used for the enzyme activity assay.

To quantify β-glucuronidase activity, we then mixed 50 µL fecalase with 100 µL potassium phosphate buffer (0.01 M, pH 7.4) and 100 µL 4-nitrophenyl- β-D-glucuronide (PNPG) (1 mM, in sodium phosphate buffer). The reaction mixture was then incubated at 37 °C for 15 min, 250 µL 0.5 N NaOH was added to stop the reaction, and the absorbance was measured at 405 nm (UV–Vis spectrophotometer) [138,139]. β-glucuronidase activity was expressed as mg of PNPG/mg feces per minute.

### 4.7. Histopathological and Histochemical Examination

All histopathological/histochemical procedures and examinations were conducted at the Department of Cytology and Histology, Faculty of Veterinary Medicine, Cairo University (Giza, Egypt). The examiner was blinded for the sample groups.

Colonic tissue samples were flushed and fixed in 10% neutral buffered formalin for 72 h. Samples were trimmed and processed in serial grades of ethanol and cleared in xylene. The samples were then infiltrated and embedded into paraplast tissue-embedding media. Five-micrometer-thick tissue sections were cut by rotatory microtome for demonstration of intestinal wall layers in different samples.

Tissue sections were stained by hematoxylin and eosin (H&E, Tokyo, Japan) as a general morphological examination staining method and by Alcian blue (pH 2.5) for quantitative analysis of goblet cells’ reactive acidic mucins, then they were examined microscopically in a blinded manner. All standard procedures for sample fixation and staining were conducted according to Culling [140].

For the histochemical analysis of reactive mucin, six random non-overlapping fields of colonic mucosa from each group were selected and scanned for the determination of area-based percentage of reactive mucins and goblet cells in Alcian-blue-stained (pH 2.5) tissue sections [141].

All light microscopic examinations and data were obtained by the Leica Application module for histological analysis attached to the full HD microscopic imaging system (Leica Microsystems GmbH, Wetzlar, Germany).

### 4.8. DNA Extraction and Quantification

Twenty-four fecal samples from different treatment groups (representing weeks 0 and 4) were subjected to DNA extraction. DNA was extracted by the QIAamp DNA Stool Minikit for microbial analysis (Qiagen, Hilden, Germany) and immediately stored at −20 °C.

The nucleic acid concentration and purity (260/280 and 260/230 ratios) of each sample were quantified in a nanodrop spectrophotometer. For DNA sequencing, the DNA was further quantified in a Qubit^®^ fluorometer (Life technologies, Carlsbad, CA, USA) by the Qubit dsDNA Hs assay kit (Life Technologies Corporation, Eugene, OR, USA).

### 4.9. 16S rRNA Amplicon Sequencing

The concentrated DNA, of high quality and concentration, from each of the fecal samples, was sequenced at The Egyptian Center for Genome and Microbiome Research, Cairo, Egypt, in an iSeq™100 platform (Illumina, San Diego, CA, USA). The 2 × 150 bp paired-end protocol was followed, and an Illumina amplicon library was generated according to the Illumina16S rRNA library preparation protocol. The variable regions V3–V4 of bacterial 16S rRNA gene were amplified with degenerate PCR primers 515F (5′-TCGTCGGCAGCGTCAGATGTGTATAAGAGACAGGTGYCAGCMGCCGCGGTA-3′) and 806R (5′-GTCTCGTGGGCTCGGAGATGTGTATAAGAGACAGGGACTACHVGGGTWTCTAAT-3). 

PCR cycling conditions were as follows: 95 °C for 3 min, 25 cycles of 95 °C for 30 s, 52 °C for 30 s, 72 °C for 30 s, and a final extension for 10 min at 72 °C. The PCR products were purified with AmpureXP beads (Beckman Coulter, Brea, CA, USA) and eluted in 10 mM Tris elution buffer (pH 8.5). Subsequently, dual indices and Nextera XT Index primers were attached by PCR (95 °C for 3 min, 8 cycles of 95 °C for 30 s, 55 °C for 30 s, 72 °C for 30 s, and a final extension for 10 min at 72 °C. Libraries were quantified in a Qubit^®^ fluorometer (Life technologies, Carlsbad, CA, USA), and their DNA integrity was further checked by agarose gel electrophoresis. The validated libraries were finally pooled in equimolar proportions in a single amplicon library, which was loaded in an Illumina iSeq™ 100 i1 Cartridge kit (Illumina, San Diego, CA, USA).

### 4.10. Microbiome Analysis

The raw sequence reads obtained from the iSeq instrument, after automated base calling by built-in BaseSpace software (Version 7.1.0) (Illumina, San Diego, CA, USA), were primarily analyzed by the Mothur software package v.1.36.1 [142] based on the MiSeq standard operating procedure (URL: https://mothur.org/wiki/miseq_sop, accessed on 25 April 2023) [143,144]. Short sequences, sequences that had unknown base pairs (sequences with N’s), and those with at least one ambiguous base or more than eight homopolymers were removed.

Mothur taxonomic assignments were made against version 123 of the SILVA bacterial database [145] based on the Wang approach [146]. Sequences were then clustered into operational taxonomic units (OTUs) based on 97% sequence similarity cutoff, and chimeric sequences were identified and removed using UCHIME [147]. Of note, SILVA v. 123 uses traditional taxon names, some of which were updated in 2021 [148]. The use of traditional names allows comparison with current and past studies; however, we provide a list of renamed phyla (Table S2).

OTU abundance and consensus taxonomy output files were analyzed by MicrobiomeAnalyst (version 2.0)—a web-based tool for the statistical and visual analysis of microbiota data [65]. Reads were initially denoised by filters of a minimum read filtering of 20% prevalence with a count of 4. Sample reads were normalized and rarefied to the minimum library size, then data scaling (total sum scaling) was performed before the final differential abundance analysis. A low-variance filter was set at 5% for standard deviation. Alpha diversity profiling was conducted by the observed OTUs, Chao1 richness estimators, and Simpson and Shannon’s indices, while statistical significance was tested by the Kruskal–Wallis method.

Beta diversity analysis was assessed by the permutational multivariate analysis of variance (PERMANOVA) statistical method, based on Bray–Curtis dissimilarity, and visualized by principal coordinate plots. Linear discriminant analysis effect size (LEfSe) was used to identify taxa with significant differences with default cutoffs. *p* values were adjusted by the false discovery rate (FDR) method [149].

### 4.11. Fecal Metabolite Profiling by Gas Chromatography Coupled with Mass Spectrometry Detection (GC-MS)

Seventy-two stool samples (triplicates of 24 samples) were processed for GC-MS metabolite analysis. For each sample, 750 µL of cold ethanol/20 mM phosphate extraction buffer (85:15 *v*/*v*) was added to 250 mg stool, and the mixture was vortexed for 2 min, homogenized for 2 min, and then centrifuged at 15,000 rpm at 4 °C for 15 min. The supernatant was then collected, and a 100 µL aliquot was mixed with 5 μL xylitol (1 mg/mL, as internal standard) and incubated with 200 μL of cold acetonitrile for 30 min at 4 °C, then centrifuged at 15,000 rpm for 10 min. The supernatant (100 μL) was collected and completely dried in a speed vacuum concentrator (Labconco Centrivap, Brermen, Germany).

For metabolite derivatization, 70 μL of N-methyl-N-(trimethylsilyl)-trifluoroacetamide (MSTFA), including 1% trimethylchlorosilane (TMCS) and 70 μL pyridine, was added to the dried aliquot and mixed, followed by incubation at 60 °C for 45 min.

Samples were then analyzed in a Shimadzu GC-17A gas chromatograph coupled with a Shimadzu QP5050A mass spectrometer. Silylated derivatives were separated on an Rtx-5MS (30 m length, 0.25 mm inner diameter, and 0.25 lm film) column. Samples were injected under 1:15 split-mode conditions: injector, 280 °C; column oven set at 80 °C for 2 min; and rate, 5 °C/min to 315 °C. Samples were kept at 315 °C for 12 min, with the helium carrier gas flow set at 1 mL/min. The transfer line and ion-source temperatures were set at 280 and 180 °C, respectively.

### 4.12. GC-MS Data Analysis

MS peak abundance of silylated metabolites were extracted with the MS Dial program, following the exact parameters settings for GC-MS [150]. The SIMCA-P version 14.1 software package (Umetrics, Umeå, Sweden) was used to export the aligned peak abundance data table, which was further exported for PCA and OPLS-DA [151]. All variables were mean-centered and scaled to Pareto variance.

### 4.13. Data Visualization and Statistical Analysis

Overall, normally distributed data with sample size greater than six were analyzed by parametric tests for significance (Student’s *t*-test and ANOVA, for double or multiple variables, respectively), while data from small samples (six or under) or non-normally distributed data were analyzed by Wilcoxon and Kruskal–Wallis tests for double or multiple variables, respectively. For two-variable comparisons, paired tests were used for week 0 vs. week 4 comparisons, while unpaired tests were used to compare treatment groups. For multiple variable comparisons, post-hoc pairwise tests were performed with Bonferroni or FDR *p* value adjustment for smaller or larger sample sizes, respectively. Dunn test was used as an alternative to Bonferroni adjustment for non-parametric data. In some instances, both ANOVA and Kruskal–Wallis tests were applied when the latter was not applicable because of ties.

For microbiome analysis, the LEfSe tool was used to identify differences in relative abundance between different experimental groups, using an alpha value of 0.05, followed by the Kruskal–Wallis test with a threshold of 2.5 for logarithmic linear discriminant analysis (LDA) scores. PERMANOVA was used for beta diversity analysis, as detailed above.

The statistics were performed in version 4.1.2 of R [152] and visualized in RStudio [[153], version 2022.02.0, Build 443] or MicrobiomeAnalyst (which uses R scripts). Correlations were identified by Spearman’s rank correlation coefficient. A list of R packages used is provided in Table S3.

### 4.14. Sequence Deposition

All sequences were deposited to the NCBI sequence-reads archive (SRA), under project # PRJNA952221 and biosample numbers SAMN34069103–SAMN34069126.

## 5. Conclusions

In conclusion, this study allowed a head-to-head comparison of the effects of pomegranate and that of BaP on colonic tissues, mucin content, and on the general balance of the colonic microbiota and metabolome of adult Sprague–Dawley rats. The four weeks of the experiment, representing a substantial time in the life span of an adult rat, allowed the demonstration of animal age as a major predictor of microbiome and metabolome variations. In addition, the paired experimental design allowed controlling for inter-individual variations and cage effects by self-comparing each animal or cage in week 4 vs. week 0, thus leading to the resolution of treatment-derived variations. Finally, using various correlation and clustering analyses underscored microbiome–metabolome associations and highlighted microbiome co-abundance clusters and potential metabotypes that represent prospects for biomarker discovery. This work establishes a model for BaP-induced colon pathologies to assess the potential microbiota-mediated chemoprotective role of pomegranate juice (here) and other nutraceuticals and functional foods (in future studies).

## Figures and Tables

**Table 1:** Microbiome clusters (visualized in Figure 12).

Cluster	Major Members
Cluster 1: *Lactobacillaceae* and *Prevoteallaceae*	*Enterorhabdus*, Bacteroidales S24-7 group, *Prevotellaceae*, *Lactobacillaceae*, *Family XIII UCG-001*, *Lachnospiraceae*, *Anaerovibrio*
Cluster 2: *Erysipelotrichaceae*	*Streptococcus*, *Family XIII AD3011 group*, *Erysipelotrichaceae UCG-003*, *Holdemanella*, *Turicibacter*, unclassified *Erysipelotrichaceae*
Cluster 3: *Collinsella*_CAG	*Collinsella*, Unclassified Bacteroidia, [Eubacterium] coprostanoligenes group, *Treponema 2*
Cluster 4: *Ruminococcaceae 1*	*Parabacteroides*, *Anaerovorax*, *Romboutsia*, unclassified *Peptostreptococcaceae*, *Ruminiclostridium 5*, *Ruminococcaceae UCG-005*, *Ruminococcaceae UCG-013*, *Ruminococcaceae UCG-014*, unclassified Clostridiales family, *Phascolarctobacterium*, *Veillonella*, unclassified Selenomonadales, unclassified Anaeroplasmatales, unclassified Mollicutes
Cluster 5: *Ruminococcaceae 2*	*Anaerotruncus*, *Ruminococcus 1*, unclassified *Ruminococcaceae*
Cluster 6:	*Allobaculum*, *Erysipelatoclostridium*

## Data Availability

Sequence datasets generated for this study are deposited at NCBI-SRA (PRJNA952221, as detailed under Materials and Methods). All raw data used in this manuscript or figures are provided as Supplementary Data Files.

## Supplementary Materials

The following supporting information can be downloaded at: https://www.mdpi.com/article/10.3390/ijms241310691/s1.

## References

[1] Short M.W., Layton M.C., Teer B.N., Domagalski J.E. (2015). Colorectal cancer screening and surveillance. Am. Fam. Physician.

[2] Rawla P., Sunkara T., Barsouk A. (2019). Epidemiology of colorectal cancer: Incidence, mortality, survival, and risk factors. Prz. Gastroenterol..

[3] Wang S., Chanock S., Tang D., Li Z., Edwards S., Jedrychowski W., Perera F.P. (2010). Effect of gene-environment Interactions on mental development in African American, Dominican, and Caucasian mothers and newborns. Ann. Hum. Genet..

[4] Sung H., Ferlay J., Siegel R.L., Laversanne M., Soerjomataram I., Jemal A., Bray F. (2021). Global cancer statistics 2020: GLOBOCAN estimates of incidence and mortality worldwide for 36 cancers in 185 countries. CA Cancer J. Clin..

[5] Ugai T., Sasamoto N., Lee H.Y., Ando M., Song M., Tamimi R.M., Kawachi I., Campbell P.T., Giovannucci E.L., Weiderpass E. (2022). Is early-onset cancer an emerging global epidemic? Current evidence and future implications. Nat. Rev. Clin. Oncol..

[6] Bray F., Jemal A., Grey N., Ferlay J., Forman D. (2012). Global cancer transitions according to the human development index (2008–2030): A population-based study. Lancet Oncol..

[7] Gado A., Ebeid B., Abdelmohsen A., Axon A. (2014). Colorectal cancer in Egypt is commoner in young people: Is this cause for alarm?. Alex. J. Med..

[8] Abou-Zeid A.A., Khafagy W., Marzouk D.M., Alaa A., Mostafa I., Ela M.A. (2002). Colorectal cancer in Egypt. Dis. Colon Rectum.

[9] Makhlouf N.A., Abdel-Gawad M., Mahros A.M., Lashen S.A., Zaghloul M., Eliwa A., Elshemy E.E., Ali-Eldin Z., Abdeltawab D., El-Raey F. (2021). Colorectal cancer in Arab world: A systematic review. World J. Gastrointest. Oncol..

[10] Kerber R.A., Neklason D.W., Samowitz W.S., Burt R.W. (2005). Frequency of familial colon cancer and hereditary nonpolyposis colorectal cancer (lynch syndrome) in a large population database. Fam. Cancer.

[11] Jasperson K.W., Tuohy T.M., Neklason D.W., Burt R.W. (2010). Hereditary and familial colon cancer. Gastroenterology.

[12] Hadjipetrou A., Anyfantakis D., Galanakis C.G., Kastanakis M., Kastanakis S. (2017). Colorectal cancer, screening and primary care: A mini literature review. World J. Gastroenterol..

[13] Glade M.J. (1999). Food, nutrition, and the prevention of cancer: A global perspective. American Institute for Cancer Research/World Cancer Research Fund, American Institute for Cancer Research, 1997. Nutrition.

[14] Bishehsari F., Mahdavinia M., Vacca M., Malekzadeh R., Mariani-Costantini R. (2014). Epidemiological transition of colorectal cancer in developing countries: Environmental factors, molecular pathways, and opportunities for prevention. World J. Gastroenterol..

[15] Ma Y., Harrad S. (2015). Spatiotemporal analysis and human exposure assessment on polycyclic aromatic hydrocarbons in indoor air, settled house dust, and diet: A review. Environ. Int..

[16] Sinha R., Cross A., Curtin J., Zimmerman T., McNutt S., Risch A., Holden J. (2005). Development of a food frequency questionnaire module and databases for compounds in cooked and processed meats. Mol. Nutr. Food Res..

[17] Key T.J., Bradbury K.E., Perez-Cornago A., Sinha R., Tsilidis K.K., Tsugane S. (2020). Diet, nutrition, and cancer risk: What do we know and what is the way forward?. BMJ.

[18] IARC Working Group on the Evaluation of Carcinogenic Risks to Humans (2010). Some non-heterocyclic polycyclic aromatic hydrocarbons and some related exposures. IARC Monographs on the Evaluation of Carcinogenic Risks to Humans.

[19] Renner R. (1999). EPA to strengthen persistent, bioaccumulative, and toxic pollutant controls-Mercury first to be targeted. Environ. Sci. Technol..

[20] Conney A.H., Chang R.L., Jerina D.M., Wei S.J. (1994). Studies on the metabolism of benzo[a]pyrene and dose-dependent differences in the mutagenic profile of its ultimate carcinogenic metabolite. Drug Metab. Rev..

[21] Phillips D.H. (1999). Polycyclic aromatic hydrocarbons in the diet. Mutat. Res..

[22] Phillips D.H. (2002). Smoking-related DNA and protein adducts in human tissues. Carcinogenesis.

[23] Kazerouni N., Sinha R., Hsu C.H., Greenberg A., Rothman N. (2001). Analysis of 200 food items for benzo[a]pyrene and estimation of its intake in an epidemiologic study. Food Chem. Toxicol..

[24] Ramesh A., Walker S.A., Hood D.B., Guillen M.D., Schneider K., Weyand E.H. (2004). Bioavailability and risk assessment of orally ingested polycyclic aromatic hydrocarbons. Int. J. Toxicol..

[25] Alomirah H., Al-Zenki S., Al-Hooti S., Zaghloul S., Sawaya W., Ahmed N., Kannan K. (2011). Concentrations and dietary exposure to polycyclic aromatic hydrocarbons (PAHs) from grilled and smoked foods. Food Control.

[26] Collins J.F., Brown J.P., Dawson S.V., Marty M.A. (1991). Risk assessment for benzo[a]pyrene. Regul. Toxicol. Pharmacol..

[27] Ellard S., Mohammed Y., Dogra S., Wolfel C., Doehmer J., Parry J.M. (1991). The use of genetically engineered V79 Chinese hamster cultures expressing rat liver CYP1A1, 1A2 and 2B1 cDNAs in micronucleus assays. Mutagenesis.

[28] Wester P.W., Muller J.J., Slob W., Mohn G.R., Dortant P.M., Kroese E.D. (2012). Carcinogenic activity of benzo[a]pyrene in a 2 year oral study in Wistar rats. Food Chem. Toxicol..

[29] Uno S., Makishima M. (2009). Benzo [a] pyrene toxicity and inflammatory disease. Curr. Rheumatol. Rev..

[30] Genies C., Maitre A., Lefebvre E., Jullien A., Chopard-Lallier M., Douki T. (2013). The extreme variety of genotoxic response to benzo[a]pyrene in three different human cell lines from three different organs. PLoS ONE.

[31] Miller K.P., Ramos K.S. (2001). Impact of cellular metabolism on the biological effects of benzo[a]pyrene and related hydrocarbons. Drug Metab. Rev..

[32] Kleiner H.E., Vulimiri S.V., Hatten W.B., Reed M.J., Nebert D.W., Jefcoate C.R., DiGiovanni J. (2004). Role of cytochrome p4501 family members in the metabolic activation of polycyclic aromatic hydrocarbons in mouse epidermis. Chem. Res. Toxicol..

[33] Harris D.L., Washington M.K., Hood D.B., Roberts L.J., Ramesh A. (2009). Dietary fat-influenced development of colon neoplasia in Apc*^Min^* mice exposed to benzo(a)pyrene. Toxicol. Pathol..

[34] Diggs D.L., Huderson A.C., Harris K.L., Myers J.N., Banks L.D., Rekhadevi P.V., Niaz M.S., Ramesh A. (2011). Polycyclic aromatic hydrocarbons and digestive tract cancers: A perspective. J. Environ. Sci. Health C Environ. Carcinog. Ecotoxicol. Rev..

[35] Sonoda J., Seki Y., Hakura A., Hosokawa S. (2015). Time course of the incidence/multiplicity and histopathological features of murine colonic dysplasia, adenoma and adenocarcinoma induced by benzo[a]pyrene and dextran sulfate sodium. J. Toxicol. Pathol..

[36] Fu Z., Shrubsole M.J., Smalley W.E., Wu H., Chen Z., Shyr Y., Ness R.M., Zheng W. (2011). Association of meat intake and meat-derived mutagen exposure with the risk of colorectal polyps by histologic type. Cancer Prev. Res..

[37] Gunter M.J., Probst-Hensch N.M., Cortessis V.K., Kulldorff M., Haile R.W., Sinha R. (2005). Meat intake, cooking-related mutagens and risk of colorectal adenoma in a sigmoidoscopy-based case-control study. Carcinogenesis.

[38] Sachse C., Smith G., Wilkie M.J., Barrett J.H., Waxman R., Sullivan F., Forman D., Bishop D.T., Wolf C.R., Colorectal Cancer Study G. (2002). A pharmacogenetic study to investigate the role of dietary carcinogens in the etiology of colorectal cancer. Carcinogenesis.

[39] Nasri H., Baradaran A., Shirzad H., Rafieian-Kopaei M. (2014). New concepts in nutraceuticals as alternative for pharmaceuticals. Int. J. Prev. Med..

[40] Fantini M., Benvenuto M., Masuelli L., Frajese G.V., Tresoldi I., Modesti A., Bei R. (2015). *In vitro* and *in vivo* antitumoral effects of combinations of polyphenols, or polyphenols and anticancer drugs: Perspectives on cancer treatment. Int. J. Mol. Sci..

[41] Thathsarani N., Nayanajeehwi G., Jayawardena U., Nilakarawasam N., Jayasinghe C.D. (2020). Cancer chemoprevention through functional food of plant origin. Asian J. Pharm. Pharmacol..

[42] Teibo J.O., Ayinde K.S., Olaoba O.T., Adelusi T.I., Teibo T.K.A., Bamikunle M.V., Jimoh Y.A., Alghamdi S., Abdulaziz O., Rauf A. (2021). Functional foods’ bioactive components and their chemoprevention mechanism in cervical, breast, and liver cancers: A systematic review. Funct. Food Health Dis..

[43] AlAli M., Alqubaisy M., Aljaafari M.N., AlAli A.O., Baqais L., Molouki A., Abushelaibi A., Lai K.S., Lim S.E. (2021). Nutraceuticals: Transformation of conventional foods into health promoters/disease preventers and safety considerations. Molecules.

[44] Seeram N.P., Adams L.S., Henning S.M., Niu Y., Zhang Y., Nair M.G., Heber D. (2005). In vitro antiproliferative, apoptotic and antioxidant activities of punicalagin, ellagic acid and a total pomegranate tannin extract are enhanced in combination with other polyphenols as found in pomegranate juice. J. Nutr. Biochem..

[45] Sallam I.E., Abdelwareth A., Attia H., Aziz R.K., Homsi M.N., von Bergen M., Farag M.A. (2021). Effect of gut microbiota biotransformation on dietary tannins and human health implications. Microorganisms.

[46] Lansky E.P., Jiang W., Mo H., Bravo L., Froom P., Yu W., Harris N.M., Neeman I., Campbell M.J. (2005). Possible synergistic prostate cancer suppression by anatomically discrete pomegranate fractions. Investig. New Drugs.

[47] Viladomiu M., Hontecillas R., Lu P., Bassaganya-Riera J. (2013). Preventive and prophylactic mechanisms of action of pomegranate bioactive constituents. Evid. Based Complement Alternat. Med..

[48] Espin J.C., Gonzalez-Barrio R., Cerda B., Lopez-Bote C., Rey A.I., Tomas-Barberan F.A. (2007). Iberian pig as a model to clarify obscure points in the bioavailability and metabolism of ellagitannins in humans. J. Agric. Food Chem..

[49] Tomas-Barberan F.A., Gonzalez-Sarrias A., Garcia-Villalba R., Nunez-Sanchez M.A., Selma M.V., Garcia-Conesa M.T., Espin J.C. (2017). Urolithins, the rescue of “old” metabolites to understand a “new” concept: Metabotypes as a nexus among phenolic metabolism, microbiota dysbiosis, and host health status. Mol. Nutr. Food Res..

[50] Espin J.C., Larrosa M., Garcia-Conesa M.T., Tomas-Barberan F. (2013). Biological significance of urolithins, the gut microbial ellagic acid-derived metabolites: The evidence so far. Evid. Based Complement. Alternat. Med..

[51] Tomas-Barberan F.A., Selma M.V., Espin J.C. (2016). Interactions of gut microbiota with dietary polyphenols and consequences to human health. Curr. Opin. Clin. Nutr. Metab. Care.

[52] Syed D.N., Afaq F., Mukhtar H. (2007). Pomegranate derived products for cancer chemoprevention. Semin. Cancer Biol..

[53] Kawaii S., Lansky E.P. (2004). Differentiation-promoting activity of pomegranate (*Punica granatum*) fruit extracts in HL-60 human promyelocytic leukemia cells. J. Med. Food.

[54] Mehta R., Lansky E.P. (2004). Breast cancer chemopreventive properties of pomegranate (*Punica granatum*) fruit extracts in a mouse mammary organ culture. Eur. J. Cancer Prev..

[55] Malik A., Mukhtar H. (2006). Prostate cancer prevention through pomegranate fruit. Cell Cycle.

[56] Modaeinama S., Abasi M., Abbasi M.M., Jahanban-Esfahlan R. (2015). Anti tumoral properties of *Punica granatum* (Pomegranate) peel extract on different human cancer cells. Asian Pac. J. Cancer Prev..

[57] Afaq F., Saleem M., Krueger C.G., Reed J.D., Mukhtar H. (2005). Anthocyanin- and hydrolyzable tannin-rich pomegranate fruit extract modulates MAPK and NF-kappaB pathways and inhibits skin tumorigenesis in CD-1 mice. Int. J. Cancer.

[58] Larrosa M., Tomas-Barberan F.A., Espin J.C. (2006). The dietary hydrolysable tannin punicalagin releases ellagic acid that induces apoptosis in human colon adenocarcinoma Caco-2 cells by using the mitochondrial pathway. J. Nutr. Biochem..

[59] Kasimsetty S.G., Bialonska D., Reddy M.K., Ma G., Khan S.I., Ferreira D. (2010). Colon cancer chemopreventive activities of pomegranate ellagitannins and urolithins. J. Agric. Food Chem..

[60] Adams L.S., Seeram N.P., Aggarwal B.B., Takada Y., Sand D., Heber D. (2006). Pomegranate juice, total pomegranate ellagitannins, and punicalagin suppress inflammatory cell signaling in colon cancer cells. J. Agric. Food Chem..

[61] Moreira H., Slezak A., Szyjka A., Oszmianski J., Gasiorowski K. (2017). Antioxidant and cancer chemopreventive activities of cistus and pomegranate polyphenols. Acta. Pol. Pharm..

[62] Boateng J., Verghese M., Shackelford L., Walker L.T., Khatiwada J., Ogutu S., Williams D.S., Jones J., Guyton M., Asiamah D. (2007). Selected fruits reduce azoxymethane (AOM)-induced aberrant crypt foci (ACF) in Fisher 344 male rats. Food Chem. Toxicol..

[63] Kohno H., Suzuki R., Yasui Y., Hosokawa M., Miyashita K., Tanaka T. (2004). Pomegranate seed oil rich in conjugated linolenic acid suppresses chemically induced colon carcinogenesis in rats. Cancer Sci..

[64] Farag M.A., Abdelwareth A., Sallam I.E., El Shorbagi M., Jehmlich N., Fritz-Wallace K., Serena Schäpe S., Rolle-Kampczyk U., Ehrlich A., Wessjohann L.A. (2020). Metabolomics reveals impact of seven functional foods on metabolic pathways in a gut microbiota model. J. Adv. Res..

[65] Dhariwal A., Chong J., Habib S., King I.L., Agellon L.B., Xia J. (2017). MicrobiomeAnalyst: A web-based tool for comprehensive statistical, visual and meta-analysis of microbiome data. Nucleic Acids Res..

[66] Huderson A.C., Myers J.N., Niaz M.S., Washington M.K., Ramesh A. (2013). Chemoprevention of benzo(a)pyrene-induced colon polyps in ApcMin mice by resveratrol. J. Nutr. Biochem..

[67] Banks L.D., Amoah P., Niaz M.S., Washington M.K., Adunyah S.E., Ramesh A. (2016). Olive oil prevents benzo(a)pyrene [B(a)P]-induced colon carcinogenesis through altered B(a)P metabolism and decreased oxidative damage in Apc*^Min^* mouse model. J. Nutr. Biochem..

[68] Aboulthana W.M., Shousha W.G., Essawy E.A., Saleh M.H., Salama A.H. (2021). Assessment of the anti-cancer efficiency of silver *Moringa oleifera* Leaves nano-extract against colon cancer induced chemically in rats. Asian Pac. J. Cancer Prev..

[69] Kim K.S., Kim N.Y., Son J.Y., Park J.H., Lee S.H., Kim H.R., Kim B., Kim Y.G., Jeong H.G., Lee B.M. (2019). Curcumin ameliorates benzo[a]pyrene-induced DNA damages in stomach tissues of Sprague-Dawley rats. Int. J. Mol. Sci..

[70] Aziz R.K., Hegazy S.M., Yasser R., Rizkallah M.R., ElRakaiby M.T. (2018). Drug pharmacomicrobiomics and toxicomicrobiomics: From scattered reports to systematic studies of drug-microbiome interactions. Expert Opin. Drug Metab. Toxicol..

[71] Abdelsalam N.A., Ramadan A.T., ElRakaiby M.T., Aziz R.K. (2020). Toxicomicrobiomics: The human microbiome *vs*. pharmaceutical, dietary, and environmental xenobiotics. Front. Pharmacol..

[72] Jass J.R., Roberton A.M. (1994). Colorectal mucin histochemistry in health and disease: A critical review. Pathol. Int..

[73] Mohamed M.S., Mabrok H.B. (2021). Protective effect of pomegranate peel powder against gastric ulcer in rats. Biointerface Res. Appl. Chem..

[74] Bastide N.M., Naud N., Nassy G., Vendeuvre J.L., Tache S., Gueraud F., Hobbs D.A., Kuhnle G.G., Corpet D.E., Pierre F.H. (2017). Red wine and pomegranate extracts suppress cured meat promotion of colonic mucin-depleted foci in carcinogen-induced Rats. Nutr. Cancer.

[75] Tortora K., Femia A.P., Romagnoli A., Sineo I., Khatib M., Mulinacci N., Giovannelli L., Caderni G. (2018). Pomegranate by-products in colorectal cancer chemoprevention: Effects in *Apc*-mutated Pirc rats and mechanistic studies in vitro and ex vivo. Mol. Nutr. Food Res..

[76] Rosillo M.A., Sanchez-Hidalgo M., Cardeno A., Aparicio-Soto M., Sanchez-Fidalgo S., Villegas I., de la Lastra C.A. (2012). Dietary supplementation of an ellagic acid-enriched pomegranate extract attenuates chronic colonic inflammation in rats. Pharmacol. Res..

[77] Rosillo M.A., Sanchez-Hidalgo M., Cardeno A., de la Lastra C.A. (2011). Protective effect of ellagic acid, a natural polyphenolic compound, in a murine model of crohn’s disease. Biochem. Pharmacol..

[78] Flemer B., Gaci N., Borrel G., Sanderson I.R., Chaudhary P.P., Tottey W., O’Toole P.W., Brugere J.F. (2017). Fecal microbiota variation across the lifespan of the healthy laboratory rat. Gut Microbes.

[79] Andreollo N.A., Santos E.F., Araujo M.R., Lopes L.R. (2012). Rat’s age versus human’s age: What is the relationship?. Arq. Bras. Cir. Dig..

[80] Sengupta P. (2013). The laboratory rat: Relating its age with human’s. Int. J. Prev. Med..

[81] Quinn R. (2005). Comparing rat’s to human’s age: How old is my rat in people years?. Nutrition.

[82] Chandra M., Riley M.G., Johnson D.E. (1992). Spontaneous neoplasms in aged Sprague-Dawley rats. Arch. Toxicol..

[83] Zapata H.J., Quagliarello V.J. (2015). The microbiota and microbiome in aging: Potential implications in health and age-related diseases. J. Am. Geriatr. Soc..

[84] Bartley J.M., Zhou X., Kuchel G.A., Weinstock G.M., Haynes L. (2017). Impact of age, caloric restriction, and influenza infection on mouse gut microbiome: An exploratory study of the role of age-related microbiome changes on influenza responses. Front. Immunol..

[85] Zhang X., Yang Y., Su J., Zheng X., Wang C., Chen S., Liu J., Lv Y., Fan S., Zhao A. (2021). Age-related compositional changes and correlations of gut microbiome, serum metabolome, and immune factor in rats. Geroscience.

[86] Lees H., Swann J., Poucher S.M., Nicholson J.K., Holmes E., Wilson I.D., Marchesi J.R. (2014). Age and microenvironment outweigh genetic influence on the Zucker rat microbiome. PLoS ONE.

[87] Sgro M., Iacono G., Yamakawa G.R., Kodila Z.N., Marsland B.J., Mychasiuk R. (2022). Age matters: Microbiome depletion prior to repeat mild traumatic brain injury differentially alters microbial composition and function in adolescent and adult rats. PLoS ONE.

[88] Song E.M., Byeon J.S., Lee S.M., Yoo H.J., Kim S.J., Lee S.H., Chang K., Hwang S.W., Yang D.H., Jeong J.Y. (2018). Fecal fatty acid profiling as a potential new screening biomarker in patients with colorectal cancer. Dig. Dis. Sci..

[89] Varma V.S., Shabtay A., Yishay M., Mizrahi I., Shterzer N., Freilich S., Medina S., Agmon R., Laor Y. (2018). Diet supplementation with pomegranate peel extract altered odorants emission from fresh and incubated calves’ feces. Front. Sustain. Food Syst..

[90] Marhuenda-Munoz M., Laveriano-Santos E.P., Tresserra-Rimbau A., Lamuela-Raventos R.M., Martinez-Huelamo M., Vallverdu-Queralt A. (2019). Microbial phenolic metabolites: Which molecules actually have an effect on human health?. Nutrients.

[91] Serra A., Macia A., Romero M.P., Angles N., Morello J.R., Motilva M.J. (2011). Distribution of procyanidins and their metabolites in rat plasma and tissues after an acute intake of hazelnut extract. Food Funct..

[92] Zhao L., Xiao H.T., Mu H.X., Huang T., Lin Z.S., Zhong L.L.D., Zeng G.Z., Fan B.M., Lin C.Y., Bian Z.X. (2017). Magnolol, a natural polyphenol, attenuates dextran sulfate sodium-induced colitis in mice. Molecules.

[93] Vasilopoulos S., Dokou S., Papadopoulos G.A., Savvidou S., Christaki S., Kyriakoudi A., Dotas V., Tsiouris V., Bonos E., Skoufos I. (2022). Dietary supplementation with pomegranate and onion aqueous and cyclodextrin encapsulated extracts affects broiler performance parameters, welfare and meat characteristics. Poultry.

[94] Dinh D.M., Volpe G.E., Duffalo C., Bhalchandra S., Tai A.K., Kane A.V., Wanke C.A., Ward H.D. (2015). Intestinal microbiota, microbial translocation, and systemic inflammation in chronic HIV infection. J. Infect. Dis..

[95] Schaubeck M., Clavel T., Calasan J., Lagkouvardos I., Haange S.B., Jehmlich N., Basic M., Dupont A., Hornef M., von Bergen M. (2016). Dysbiotic gut microbiota causes transmissible crohn’s disease-like ileitis independent of failure in antimicrobial defence. Gut.

[96] Chen W., Liu F., Ling Z., Tong X., Xiang C. (2012). Human intestinal lumen and mucosa-associated microbiota in patients with colorectal cancer. PLoS ONE.

[97] Cheng S., Hu J., Wu X., Pan J.A., Jiao N., Li Y., Huang Y., Lin X., Zou Y., Chen Y. (2021). Altered gut microbiome in FUT2 loss-of-function mutants in support of personalized medicine for inflammatory bowel diseases. J. Genet. Genom..

[98] Zhu Q., Jin Z., Wu W., Gao R., Guo B., Gao Z., Yang Y., Qin H. (2014). Analysis of the intestinal lumen microbiota in an animal model of colorectal cancer. PLoS ONE.

[99] Zhang Y., Dong Y., Lu P., Wang X., Li W., Dong H., Fan S., Li D. (2021). Gut metabolite Urolithin A mitigates ionizing radiation-induced intestinal damage. J. Cell Mol. Med..

[100] He T., Cheng X., Xing C. (2021). The gut microbial diversity of colon cancer patients and the clinical significance. Bioengineered.

[101] Park J.Y., Seo H., Kang C.S., Shin T.S., Kim J.W., Park J.M., Kim J.G., Kim Y.K. (2022). Dysbiotic change in gastric microbiome and its functional implication in gastric carcinogenesis. Sci. Rep..

[102] Gevers D., Kugathasan S., Denson L.A., Vazquez-Baeza Y., Van Treuren W., Ren B., Schwager E., Knights D., Song S.J., Yassour M. (2014). The treatment-naive microbiome in new-onset crohn’s disease. Cell Host Microbe.

[103] Li Y., Poroyko V., Yan Z., Pan L., Feng Y., Zhao P., Xie Z., Hong L. (2016). Characterization of intestinal microbiomes of hirschsprung’s disease patients with or without enterocolitis using Illumina-MiSeq high-throughput sequencing. PLoS ONE.

[104] Bamola V.D., Kapardar R., Lal B., Sharma A., Chaudhry R. (2022). A metagenomic assessment of gut microbiota in Indian colon cancer patients. J. Cancer Res. Ther..

[105] Cortez R.V., Moreira L.N., Padilha M., Bibas M.D., Toma R.K., Porta G., Taddei C.R. (2020). Gut microbiome of children and adolescents with primary sclerosing cholangitis in association with ulcerative colitis. Front. Immunol..

[106] Li Z., Henning S.M., Lee R.P., Lu Q.Y., Summanen P.H., Thames G., Corbett K., Downes J., Tseng C.H., Finegold S.M. (2015). Pomegranate extract induces ellagitannin metabolite formation and changes stool microbiota in healthy volunteers. Food Funct..

[107] Lu X.Y., Han B., Deng X., Deng S.Y., Zhang Y.Y., Shen P.X., Hui T., Chen R.H., Li X., Zhang Y. (2020). Pomegranate peel extract ameliorates the severity of experimental autoimmune encephalomyelitis via modulation of gut microbiota. Gut Microbes.

[108] Kendall A.I. (1909). Some observations on the study of the intestinal bacteria. J. Biol. Chem..

[109] Behr C., Slopianka M., Haake V., Strauss V., Sperber S., Kamp H., Walk T., Beekmann K., Rietjens I., van Ravenzwaay B. (2019). Analysis of metabolome changes in the bile acid pool in feces and plasma of antibiotic-treated rats. Toxicol. Appl. Pharmacol..

[110] Mao S., Zhang R., Wang D., Zhu W. (2012). The diversity of the fecal bacterial community and its relationship with the concentration of volatile fatty acids in the feces during subacute rumen acidosis in dairy cows. BMC Vet. Res..

[111] Wei X., Jiang S., Zhao X., Li H., Lin W., Li B., Lu J., Sun Y., Yuan J. (2016). Community-metabolome correlations of gut microbiota from child-turcotte-pugh of A and B patients. Front. Microbiol..

[112] Kang H.G., Jeong S.H., Cho M.H., Cho J.H. (2007). Changes of biomarkers with oral exposure to benzo(a)pyrene, phenanthrene and pyrene in rats. J. Vet. Sci..

[113] Zhao R., Long X., Yang J., Du L., Zhang X., Li J., Hou C. (2019). Pomegranate peel polyphenols reduce chronic low-grade inflammatory responses by modulating gut microbiota and decreasing colonic tissue damage in rats fed a high-fat diet. Food Funct..

[114] Blaut M., Schoefer L., Braune A. (2003). Transformation of flavonoids by intestinal microorganisms. Int. J. Vitam. Nutr. Res..

[115] Pellock S.J., Redinbo M.R. (2017). Glucuronides in the gut: Sugar-driven symbioses between microbe and host. J. Biol. Chem..

[116] Dashnyam P., Mudududdla R., Hsieh T.J., Lin T.C., Lin H.Y., Chen P.Y., Hsu C.Y., Lin C.H. (2018). β-glucuronidases of opportunistic bacteria are the major contributors to xenobiotic-induced toxicity in the gut. Sci. Rep..

[117] Gloux K., Berteau O., El Oumami H., Beguet F., Leclerc M., Dore J. (2011). A metagenomic β-glucuronidase uncovers a core adaptive function of the human intestinal microbiome. Proc. Natl Acad. Sci. USA.

[118] Masamune H. (1934). Biochemical Studies on Carbohydrates IV. On an enzyme which catalyses the hydrolysis of biosynthetic osides of glucuronic acid. J. Biochem..

[119] Haiser H.J., Turnbaugh P.J. (2013). Developing a metagenomic view of xenobiotic metabolism. Pharmacol. Res..

[120] Nakamura J., Kubota Y., Miyaoka M., Saitoh T., Mizuno F., Benno Y. (2002). Comparison of four microbial enzymes in Clostridia and Bacteroides isolated from human feces. Microbiol. Immunol..

[121] Dabek M., McCrae S.I., Stevens V.J., Duncan S.H., Louis P. (2008). Distribution of β-glucosidase and β-glucuronidase activity and of β-glucuronidase gene *gus* in human colonic bacteria. FEMS Microbiol. Ecol..

[122] Walsh J., Olavarria-Ramirez L., Lach G., Boehme M., Dinan T.G., Cryan J.F., Griffin B.T., Hyland N.P., Clarke G. (2020). Impact of host and environmental factors on β-glucuronidase enzymatic activity: Implications for gastrointestinal serotonin. Am. J. Physiol. Gastrointest. Liver Physiol..

[123] de Moreno de LeBlanc A., Perdigon G. (2005). Reduction of beta-glucuronidase and nitroreductase activity by yoghurt in a murine colon cancer model. Biocell.

[124] Chang J., Chadwick R.W., Allison J.C., Hayes Y.O., Talley D.L., Autry C.E. (1994). Microbial succession and intestinal enzyme activities in the developing rat. J. Appl. Bacteriol..

[125] Goldin B., Dwyer J., Gorbach S.L., Gordon W., Swenson L. (1978). Influence of diet and age on fecal bacterial enzymes. Am. J. Clin. Nutr..

[126] McMahon T.F., Beierschmitt W.P., Weiner M. (1987). Changes in phase I and phase II biotransformation with age in male Fischer 344 rat colon: Relationship to colon carcinogenesis. Cancer Lett..

[127] Arumugam M., Raes J., Pelletier E., Le Paslier D., Yamada T., Mende D.R., Fernandes G.R., Tap J., Bruls T., Batto J.M. (2011). Enterotypes of the human gut microbiome. Nature.

[128] Srinivas N.R. (2013). Is pomegranate juice a potential perpetrator of clinical drug-drug interactions? Review of the in vitro, preclinical and clinical evidence. Eur. J. Drug Metab. Pharmacokinet..

[129] Andishmand H., Azadmard-Damirchi S., Hamishekar H., Torbati M., Kharazmi M.S., Savage G.P., Tan C., Jafari S.M. (2023). Nano-delivery systems for encapsulation of phenolic compounds from pomegranate peel. Adv. Colloid Interface Sci..

[130] Zuccari G., Baldassari S., Ailuno G., Turrini F., Alfei S., Caviglioli G.J.A.S. (2020). Formulation strategies to improve oral bioavailability of ellagic acid. Appl. Sci..

[131] Avramia I., Amariei S. (2022). Formulation, characterization and optimization of beta-glucan and pomegranate juice based films for its potential in diabetes. Nutrients.

[132] Huderson A.C., Rekha Devi P.V., Niaz M.S., Adunyah S.E., Ramesh A. (2019). Alteration of benzo(a)pyrene biotransformation by resveratrol in Apc*^Min/+^* mouse model of colon carcinogenesis. Investig. New Drugs.

[133] National Institutes of Health (US) (1981). NIH Guidelines for the Laboratory Use of Chemical Carcinogens.

[134] Odabasoglu F., Aslan A., Cakir A., Suleyman H., Karagoz Y., Halici M., Bayir Y. (2004). Comparison of antioxidant activity and phenolic content of three lichen species. Phytother. Res..

[135] Blainski A., Lopes G.C., de Mello J.C. (2013). Application and analysis of the folin ciocalteu method for the determination of the total phenolic content from *Limonium brasiliense* L.. Molecules.

[136] Farag M.A., Ammar N.M., Kholeif T.E., Metwally N.S., El-Sheikh N.M., Wessjohann L.A., Abdel-Hamid A.Z. (2017). Rats’ urinary metabolomes reveal the potential roles of functional foods and exercise in obesity management. Food Funct..

[137] Ruehl-Fehlert C., Kittel B., Morawietz G., Deslex P., Keenan C., Mahrt C.R., Nolte T., Robinson M., Stuart B.P., Deschl U. (2003). Revised guides for organ sampling and trimming in rats and mice–Part 1. Exp. Toxicol. Pathol..

[138] Lee D.S., Kim Y.S., Ko C.N., Cho K.H., Bae H.S., Lee K.S., Kim J.J., Park E.K., Kim D.H. (2002). Fecal metabolic activities of herbal components to bioactive compounds. Arch. Pharm. Res..

[139] Yoo D.H., Kim I.S., Van Le T.K., Jung I.H., Yoo H.H., Kim D.H. (2014). Gut microbiota-mediated drug interactions between lovastatin and antibiotics. Drug Metab. Dispos..

[140] Culling C.F.A. (1974). Handbook of Histopathological and Histochemical Techniques: Including Museum Techniques.

[141] Bancroft J.D., Gamble M. (2007). Theory and Practice of Histological Techniques.

[142] Schloss P.D., Westcott S.L., Ryabin T., Hall J.R., Hartmann M., Hollister E.B., Lesniewski R.A., Oakley B.B., Parks D.H., Robinson C.J. (2009). Introducing mothur: Open-source, platform-independent, community-supported software for describing and comparing microbial communities. Appl. Environ. Microbiol..

[143] Kozich J.J., Westcott S.L., Baxter N.T., Highlander S.K., Schloss P.D. (2013). Development of a dual-index sequencing strategy and curation pipeline for analyzing amplicon sequence data on the MiSeq Illumina sequencing platform. Appl. Environ. Microbiol..

[144] Caporaso J.G., Lauber C.L., Walters W.A., Berg-Lyons D., Huntley J., Fierer N., Owens S.M., Betley J., Fraser L., Bauer M. (2012). Ultra-high-throughput microbial community analysis on the Illumina HiSeq and MiSeq platforms. ISME J..

[145] Quast C., Pruesse E., Yilmaz P., Gerken J., Schweer T., Yarza P., Peplies J., Glockner F.O. (2013). The SILVA ribosomal RNA gene database project: Improved data processing and web-based tools. Nucleic Acids Res..

[146] Wang Q., Garrity G.M., Tiedje J.M., Cole J.R. (2007). Naive Bayesian classifier for rapid assignment of rRNA sequences into the new bacterial taxonomy. Appl. Environ. Microbiol..

[147] Edgar R.C., Haas B.J., Clemente J.C., Quince C., Knight R. (2011). UCHIME improves sensitivity and speed of chimera detection. Bioinformatics.

[148] Oren A., Garrity G.M. (2021). Valid publication of the names of forty-two phyla of prokaryotes. Int. J. Syst. Evol. Microbiol..

[149] Segata N., Izard J., Waldron L., Gevers D., Miropolsky L., Garrett W.S., Huttenhower C. (2011). Metagenomic biomarker discovery and explanation. Genome Biol..

[150] Otify A.M., Ibrahim R.M., Abib B., Laub A., Wessjohann L.A., Jiang Y., Farag M.A. (2023). Unveiling metabolome heterogeneity and new chemicals in 7 tomato varieties via multiplex approach of UHPLC-MS/MS, GC-MS, and UV-Vis in relation to antioxidant effects as analyzed using molecular networking and chemometrics. Food Chem..

[151] Wiklund S., Johansson E., Sjostrom L., Mellerowicz E.J., Edlund U., Shockcor J.P., Gottfries J., Moritz T., Trygg J. (2008). Visualization of GC/TOF-MS-based metabolomics data for identification of biochemically interesting compounds using OPLS class models. Anal. Chem..

[152] R Core Team (2022). R: A Language and Environment for Statistical Computing.

[153] Racine J.S. (2012). RStudio: A Platform-Independent IDE for R and Sweave. J. Appl. Econom..

