# Supplementary material for: Integrating Microbiome Analysis, Metabolomics, Bioinformatics, and Histopathology to Elucidate the Protective Effects of Pomegranate Juice against Benzo-alpha-pyrene-Induced Colon Pathologies
Source: Int J Mol Sci. 2023 Jun 27;24(13):10691. doi: 10.3390/ijms241310691 (PMC10341650; doi:10.3390/ijms241310691)
Supplement: Supplementary file 1 [file ijms-24-10691-s001.zip › HA_PomBaP_Supplementary_material_Proofs.pdf]

# Integrating Microbiome Analysis, Metabolomics, Bioinformatics, and Histopathology to Elucidate the Protective Effects of Pomegranate Juice against Benzo-alpha-pyrene–Induced Colon Pathologies

**Heba Attia[1,2], Shahira A. ElBanna[1,2], Rania A. Khattab[1], Mohamed A. Farag[3], Aymen S. Yassin[1,2], Ramy K. Aziz[1,2,4]**

[1]Department of Microbiology and Immunology, Faculty of Pharmacy, Cairo University, 11562 Cairo, Egypt; [2] Center for Genome and Microbiome Research, Faculty of Pharmacy, Cairo University, 11562 Cairo, Egypt; [3] Department of Pharmacognosy, Faculty of Pharmacy, Cairo University, 11562 Cairo, Egypt; [4] Microbiology and Immunology Research Program, Children's Cancer Hospital Egypt 57357, 11617 Cairo, Egypt

## *Supplementary Material*

1. **Supplementary Data Files:**

   **Data file S1:** Microbiome abundance and relative abundance data (as analyzed by Mothur), with raw read counts, normalized read counts, normalized week 4/week 0 ratios (for non-zero values), and taxa that are unique to either week 0 or week 4 samples.

   **Data file S2:** Metabolomic analysis results, for all samples, normalized to xylitol, and double-normalized week 4/week 0 ratios.

   **Data file S3:** Microbiome-metabolome correlation matrices

2. **Supplementary Tables:**

   **Table S1:** Results of GC-MS analysis of primary metabolites from different groups

   **Table S2:** Updated names of some bacterial phyla according to the International Code of Nomenclature of Prokaryotes (ICNP)_taxonomy 2022

   **Table S3:** List of used R packages

## 3. Supplementary Figures:


| Figure | Page |
|---|---|
| **Figure S1.** Animal weight and weight variation. | 10 |
| **Figure S2.** Fecal glucuronidase activity at the start and end of the experiment. | 11 |
| **Figure S3.** Taxonomic profile of the fecal microbiome of experimental animals. | 12 |
| **Figure S4.** Microbiome alpha diversity metrics (observed OTUs, Chao1, Shannon, and Simpson diversity indices) | 13 |
| **Figure S5.** Microbiome beta diversity analysis | 14 |
| **Figure S6.** Taxa with significant differences in relative abundance among the five groups | 15 |
| **Figure S7.** Significantly different taxa between different treatment groups of week 4, normalized to their corresponding week 0 samples. | 16 |
| **Figure S8.** Distribution of the different detected metabolite classes at week 0 and week 4 | 17 |
| **Figure S9.** PCA for four sample groups from the start and end of the experiment: four groups representing week 0, and four representing week 4), with sugars removed. | 18 |
| **Figure S10.** OPLS analyses of the sample groups, including and excluding sugars. | 19 |
| **Figure S11.** OPLS-DA for normalized groups before (Wk0) and at the end (Wk4) of the experiment | 20 |
| **Figure S12.** OPLS-DA for normalized groups before (Wk0) and at the end of (Wk4) the experiment—with sugars excluded | 20 |
| **Figure S13.** PCA for four sample groups, representing Wk0. | 21 |
| **Figure S14.** Microbiome-metabolome correlations | 22 |
| **Figure S15.** Correlation matrix of microbial taxa based on their correlation with metabolites. | 25 |
| **Figure S16.** Correlation matrix of metabolites based on their correlation with microbial taxa. | 26 |

**Table S1:** Results of GC-MS analysis of primary metabolites from different treatment groups in week 0 and week 4. The data are expressed as relative percentile (mean ± standard deviation, n = 3).

| Class | Metabolite | Wk0 | | | | Wk4 | | | |
|---|---|---|---|---|---|---|---|---|---|
| | | Wk0_U | Wk0_P | Wk0_B | Wk0_PB | Wk4_U | Wk4_P | Wk4_B | Wk4_PB |
| Acids | Butyric acid | 0.033 ± 0.026 | 0.017 ± 0.027 | 0.033 ± 0.025 | 0.015 ± 0.019 | 0.039 ± 0.031 | 0.019 ± 0.029 | 0.047 ± 0.035 | 0.016 ± 0.025 |
| | Isovaleric acid | 0.017 ± 0.008 | 0.021 ± 0.007 | 0.023 ± 0.016 | 0.015 ± 0.011 | 0.028 ± 0.009 | 0.042 ± 0.019 | 0.033 ± 0.012 | 0.034 ± 0.012 |
| | Propionic acid | 0.001 ± 0.000 | 0.014 ± 0.037 | 0.074 ± 0.073 | 0.041 ± 0.056 | 0.057 ± 0.066 | 0.020 ± 0.037 | 0.010 ± 0.028 | 0.029 ± 0.043 |
| | Valeric acid | 0.037 ± 0.023 | 0.044 ± 0.015 | 0.047 ± 0.039 | 0.020 ± 0.013 | 0.066 ± 0.020 | 0.056 ± 0.028 | 0.070 ± 0.013 | 0.063 ± 0.038 |
| | Lactic acid | 0.374 ± 0.214 | 0.345 ± 0.356 | 0.218 ± 0.176 | 0.140 ± 0.030 | 0.393 ± 0.213 | 0.232 ± 0.085 | 0.270 ± 0.207 | 0.383 ± 0.332 |
| | Caproic acid | 0.028 ± 0.020 | 0.027 ± 0.013 | 0.019 ± 0.013 | 0.008 ± 0.004 | 0.024 ± 0.008 | 0.014 ± 0.005 | 0.019 ± 0.007 | 0.026 ± 0.015 |
| | Glycolic acid | 0.025 ± 0.006 | 0.030 ± 0.007 | 0.027 ± 0.004 | 0.022 ± 0.012 | 0.027 ± 0.013 | 0.031 ± 0.016 | 0.019 ± 0.004 | 0.026 ± 0.015 |
| | alpha-Hydroxybutyric acid | 0.010 ± 0.003 | 0.011 ± 0.002 | 0.009 ± 0.002 | 0.008 ± 0.004 | 0.009 ± 0.003 | 0.009 ± 0.005 | 0.006 ± 0.003 | 0.012 ± 0.010 |
| | beta-Lactic acid | 0.019 ± 0.015 | 0.013 ± 0.008 | 0.023 ± 0.009 | 0.029 ± 0.032 | 0.006 ± 0.003 | 0.005 ± 0.001 | 0.006 ± 0.003 | 0.004 ± 0.002 |
| | 2-Hydroxyvaleric acid | 0.025 ± 0.011 | 0.022 ± 0.004 | 0.029 ± 0.010 | 0.039 ± 0.010 | 0.036 ± 0.036 | 0.026 ± 0.014 | 0.036 ± 0.019 | 0.036 ± 0.028 |
| | 2-Aminobutyric acid | 0.043 ± 0.014 | 0.054 ± 0.022 | 0.047 ± 0.017 | 0.049 ± 0.012 | 0.013 ± 0.010 | 0.040 ± 0.086 | 0.007 ± 0.007 | 0.050 ± 0.071 |
| | gamma-Hydroxybutyric acid | 0.320 ± 0.197 | 0.255 ± 0.283 | 0.394 ± 0.236 | 0.203 ± 0.134 | 0.462 ± 0.260 | 0.335 ± 0.253 | 0.524 ± 0.268 | 0.350 ± 0.232 |
| | 2-Hydroxyisocaproic acid | 0.016 ± 0.006 | 0.015 ± 0.004 | 0.015 ± 0.004 | 0.019 ± 0.005 | 0.014 ± 0.012 | 0.015 ± 0.010 | 0.010 ± 0.005 | 0.019 ± 0.014 |
| | Succinic acid | 0.257 ± 0.061 | 0.142 ± 0.057 | 0.196 ± 0.053 | 0.323 ± 0.199 | 0.088 ± 0.051 | 0.076 ± 0.029 | 0.158 ± 0.062 | 0.104 ± 0.083 |
| | 4-Hydroxybutyric acid | 0.004 ± 0.004 | 0.002 ± 0.000 | 0.005 ± 0.004 | 0.006 ± 0.005 | 0.004 ± 0.003 | 0.005 ± 0.001 | 0.007 ± 0.005 | 0.007 ± 0.005 |
| | Decanoic acid | 0.013 ± 0.004 | 0.010 ± 0.007 | 0.010 ± 0.005 | 0.007 ± 0.003 | 0.015 ± 0.012 | 0.013 ± 0.010 | 0.010 ± 0.004 | 0.021 ± 0.019 |
| | Tartaric acid | 0.018 ± 0.008 | 0.026 ± 0.008 | 0.020 ± 0.005 | 0.009 ± 0.008 | 0.004 ± 0.001 | 0.004 ± 0.003 | 0.004 ± 0.002 | 0.004 ± 0.003 |
| | beta-Phenyllactic acid | 0.007 ± 0.004 | 0.005 ± 0.001 | 0.007 ± 0.001 | 0.007 ± 0.002 | 0.005 ± 0.003 | 0.005 ± 0.005 | 0.005 ± 0.002 | 0.010 ± 0.011 |

| Class | Metabolite | Wk0 | | | | Wk4 | | | |
|---|---|---|---|---|---|---|---|---|---|
| | | Wk0_U | Wk0_P | Wk0_B | Wk0_PB | Wk4_U | Wk4_P | Wk4_B | Wk4_PB |
| | 3-Hydroxyphenylacetic acid | 0.007 ± 0.001 | 0.008 ± 0.003 | 0.008 ± 0.003 | 0.008 ± 0.002 | 0.021 ± 0.002 | 0.013 ± 0.004 | 0.018 ± 0.002 | 0.012 ± 0.003 |
| | p-Hydroxyphenylacetic acid | 0.034 ± 0.004 | 0.030 ± 0.004 | 0.034 ± 0.008 | 0.032 ± 0.006 | 0.016 ± 0.007 | 0.010 ± 0.005 | 0.010 ± 0.004 | 0.016 ± 0.005 |
| | Ribofuranose (isomer 1) | 0.157 ± 0.032 | 0.125 ± 0.024 | 0.144 ± 0.049 | 0.149 ± 0.027 | 0.039 ± 0.015 | 0.028 ± 0.009 | 0.036 ± 0.010 | 0.047 ± 0.019 |
| | 3-Hydroxyphenyl-propionic acid | 0.067 ± 0.012 | 0.053 ± 0.014 | 0.071 ± 0.020 | 0.080 ± 0.016 | 0.143 ± 0.060 | 0.205 ± 0.053 | 0.300 ± 0.092 | 0.111 ± 0.046 |
| | Azelaic acid | 0.011 ± 0.004 | 0.011 ± 0.001 | 0.011 ± 0.005 | 0.009 ± 0.002 | 0.005 ± 0.001 | 0.004 ± 0.001 | 0.004 ± 0.001 | 0.006 ± 0.001 |
| | 5-Hydroxyindoleacetic acid | 0.024 ± 0.006 | 0.012 ± 0.004 | 0.020 ± 0.006 | 0.039 ± 0.031 | 0.040 ± 0.039 | 0.059 ± 0.020 | 0.092 ± 0.057 | 0.046 ± 0.036 |
| | Dehydroabietic acid | 0.015 ± 0.008 | 0.019 ± 0.006 | 0.026 ± 0.005 | 0.030 ± 0.018 | 0.024 ± 0.023 | 0.010 ± 0.004 | 0.014 ± 0.005 | 0.018 ± 0.008 |
| | **Total** | **1.564** | **1.310** | **1.511** | **1.307** | **1.577** | **1.274** | **1.717** | **1.452** |
| **Alcohols** | Ethylene glycol | 0.024 ± 0.035 | 0.013 ± 0.033 | 0.001 ± 0.001 | 0.013 ± 0.034 | 0.002 ± 0.001 | 0.022 ± 0.040 | 0.032 ± 0.036 | 0.035 ± 0.044 |
| | Propylene glycol | 0.015 ± 0.004 | 0.015 ± 0.005 | 0.011 ± 0.001 | 0.015 ± 0.003 | 0.018 ± 0.005 | 0.019 ± 0.006 | 0.018 ± 0.005 | 0.020 ± 0.007 |
| | 1,3 Propanediol | 0.023 ± 0.012 | 0.018 ± 0.013 | 0.026 ± 0.014 | 0.017 ± 0.009 | 0.034 ± 0.012 | 0.023 ± 0.013 | 0.034 ± 0.014 | 0.021 ± 0.016 |
| | 1,4-Butanediol | 0.251 ± 0.153 | 0.355 ± 0.120 | 0.314 ± 0.126 | 0.276 ± 0.114 | 0.053 ± 0.036 | 0.071 ± 0.097 | 0.029 ± 0.018 | 0.122 ± 0.153 |
| | 1-Hexadecanol | 0.040 ± 0.057 | 0.036 ± 0.034 | 0.024 ± 0.015 | 0.050 ± 0.049 | 0.041 ± 0.048 | 0.079 ± 0.122 | 0.029 ± 0.037 | 0.030 ± 0.030 |
| | Octadecan-1-ol | 0.026 ± 0.034 | 0.025 ± 0.021 | 0.017 ± 0.007 | 0.031 ± 0.026 | 0.027 ± 0.026 | 0.049 ± 0.066 | 0.026 ± 0.018 | 0.021 ± 0.016 |
| | **Total** | **0.379** | **0.461** | **0.393** | **0.401** | **0.175** | **0.263** | **0.169** | **0.248** |
| **Aliphatic hydro-carbons** | Nonane | 0.079 ± 0.019 | 0.093 ± 0.030 | 0.086 ± 0.017 | 0.068 ± 0.011 | 0.112 ± 0.042 | 0.101 ± 0.034 | 0.122 ± 0.014 | 0.092 ± 0.041 |
| | **Total** | **0.079** | **0.093** | **0.086** | **0.068** | **0.112** | **0.101** | **0.122** | **0.092** |
| **Aromatic hydrocarbons** | Ethylbenzene | 0.009 ± 0.009 | 0.022 ± 0.011 | 0.024 ± 0.008 | 0.018 ± 0.005 | 0.028 ± 0.016 | 0.004 ± 0.006 | 0.026 ± 0.019 | 0.012 ± 0.015 |
| | p-Xylene | 0.730 ± 1.124 | 1.314 ± 1.696 | 0.856 ± 1.082 | 0.978 ± 1.047 | 0.855 ± 1.307 | 2.263 ± 2.024 | 0.868 ± 1.203 | 1.638 ± 1.594 |
| | **Total** | **0.738** | **1.336** | **0.880** | **0.995** | **0.884** | **2.267** | **0.894 ±** | **1.650** |
| **Amino acids** | l-Alanine | 0.060 ± 0.083 | 0.005 ± 0.002 | 0.003 ± 0.001 | 0.005 ± 0.002 | 0.010 ± 0.012 | 0.028 ± 0.051 | 0.011 ± 0.006 | 0.038 ± 0.069 |

| Class | Metabolite | Wk0 | | | | Wk4 | | | |
|---|---|---|---|---|---|---|---|---|---|
| | | Wk0_U | Wk0_P | Wk0_B | Wk0_PB | Wk4_U | Wk4_P | Wk4_B | Wk4_PB |
| | L-Valine | 0.011 ± 0.007 | 0.013 ± 0.004 | 0.014 ± 0.014 | 0.012 ± 0.004 | 0.004 ± 0.003 | 0.015 ± 0.017 | 0.002 ± 0.002 | 0.019 ± 0.020 |
| | Valine | 0.274 ± 0.078 | 0.451 ± 0.105 | 0.315 ± 0.074 | 0.349 ± 0.124 | 0.056 ± 0.039 | 0.120 ± 0.236 | 0.033 ± 0.012 | 0.196 ± 0.209 |
| | l-Leucine | 0.125 ± 0.052 | 0.254 ± 0.116 | 0.157 ± 0.057 | 0.148 ± 0.088 | 0.021 ± 0.015 | 0.029 ± 0.035 | 0.016 ± 0.007 | 0.099 ± 0.112 |
| | Leucine | 0.021 ± 0.006 | 0.015 ± 0.006 | 0.018 ± 0.008 | 0.018 ± 0.004 | 0.011 ± 0.003 | 0.009 ± 0.003 | 0.012 ± 0.003 | 0.014 ± 0.004 |
| | Isoleucine | 0.092 ± 0.045 | 0.165 ± 0.060 | 0.109 ± 0.042 | 0.112 ± 0.071 | 0.010 ± 0.007 | 0.031 ± 0.071 | 0.009 ± 0.005 | 0.038 ± 0.039 |
| | beta-Alanine | 0.005 ± 0.002 | 0.005 ± 0.001 | 0.005 ± 0.001 | 0.003 ± 0.001 | 0.009 ± 0.003 | 0.006 ± 0.001 | 0.008 ± 0.001 | 0.006 ± 0.001 |
| | Serine | 0.019 ± 0.013 | 0.026 ± 0.013 | 0.016 ± 0.007 | 0.016 ± 0.010 | 0.004 ± 0.003 | 0.0050. ± 007 | 0.003 ± 0.002 | 0.005 ± 0.005 |
| | L-Threonine | 0.008 ± 0.002 | 0.008 ± 0.002 | 0.015 ± 0.007 | 0.009 ± 0.003 | 0.011 ± 0.006 | 0.008 ± 0.007 | 0.014 ± 0.009 | 0.010 ± 0.007 |
| | Glycine | 0.284 ± 0.081 | 0.424 ± 0.166 | 0.322 ± 0.072 | 0.319 ± 0.102 | 0.396 ± 0.162 | 0.462 ± 0.181 | 0.396 ± 0.118 | 0.435 ± 0.208 |
| | Homoserine | 0.009 ± 0.003 | 0.014 ± 0.011 | 0.007 ± 0.002 | 0.007 ± 0.003 | 0.001 ± 0.001 | 0.009 ± 0.017 | 0.001 ± 0.000 | 0.008 ± 0.011 |
| | Aspartate | 0.014 ± 0.005 | 0.017 ± 0.006 | 0.016 ± 0.005 | 0.018 ± 0.005 | 0.005 ± 0.002 | 0.008 ± 0.005 | 0.005 ± 0.002 | 0.016 ± 0.011 |
| | DL-Phenylalanine | 0.015 ± 0.009 | 0.014 ± 0.007 | 0.017 ± 0.010 | 0.016 ± 0.005 | 0.002 ± 0.001 | 0.008 ± 0.015 | 0.002 ± 0.001 | 0.006 ± 0.006 |
| | 2-Aminoadipic acid | 0.042 ± 0.045 | 0.020 ± 0.021 | 0.044 ± 0.031 | 0.020 ± 0.031 | 0.005 ± 0.004 | 0.013 ± 0.014 | 0.003 ± 0.002 | 0.011 ± 0.014 |
| | Phenylalanine | 0.040 ± 0.019 | 0.063 ± 0.021 | 0.035 ± 0.013 | 0.038 ± 0.027 | 0.006 ± 0.004 | 0.027 ± 0.066 | 0.004 ± 0.002 | 0.015 ± 0.016 |
| | Tyrosine | 0.119 ± 0.055 | 0.154 ± 0.065 | 0.087 ± 0.037 | 0.105 ± 0.096 | 0.011 ± 0.006 | 0.082 ± 0.185 | 0.011 ± 0.004 | 0.058 ± 0.061 |
| | **Total** | **1.138** | **1.648** | **1.181** | **1.196** | **0.561** | **0.862** | **0.528** | **0.974** |
| **Amino Sugars** | N-Acetyl-D-glucosamine (isomer 2) | 0.039 ± 0.011 | 0.045 ± 0.010 | 0.04 ± 0.007 | 0.050 ± 0.002 | 0.010 ± 0.004 | 0.009 ± 0.003 | 0.013 ± 0.003 | 0.012 ± 0.003 |
| | **Total** | **0.039** | **0.045** | **0.04** | **0.05** | **0.01** | **0.009** | **0.013** | **0.012** |
| **Esters** | Tributyl acetylcitrate | 0.003 ± 0.065 | 0.052 ± 0.078 | 0.003 ± 0.006 | 0.074 ± 0.108 | 0.038 ± 0.111 | 0.140 ± 0.237 | 0.020 ± 0.045 | 0.038 ± 0.063 |
| | **Total** | **0.033** | **0.052** | **0.003** | **0.074** | **0.038** | **0.140** | **0.020** | **0.038** |

| Class | Metabolite | Wk0 | | | | Wk4 | | | |
|---|---|---|---|---|---|---|---|---|---|
| | | Wk0_U | Wk0_P | Wk0_B | Wk0_PB | Wk4_U | Wk4_P | Wk4_B | Wk4_PB |
| Fatty acids/esters | Myristic acid | 0.117 ± 0.111 | 0.310 ± 0.105 | 0.248 ± 0.033 | 0.247 ± 0.057 | 0.369 ± 0.048 | 0.370 ± 0.072 | 0.346 ± 0.048 | 0.393 ± 0.063 |
| | Pentadecanoic acid ester | 0.029 ± 0.011 | 0.030 ± 0.008 | 0.031 ± 0.003 | 0.037 ± 0.012 | 0.026 ± 0.005 | 0.022 ± 0.007 | 0.028 ± 0.006 | 0.023 ± 0.005 |
| | Palmitic acid | 0.575 ± 0.069 | 0.544 ± 0.182 | 0.483 ± 0.035 | 0.714 ± 0.392 | 0.534 ± 0.082 | 0.474 ± 0.118 | 0.483 ± 0.065 | 0.448 ± 0.104 |
| | Linoleic acid | 0.394 ± 0.061 | 0.320 ± 0.067 | 0.325 ± 0.069 | 0.495 ± 0.145 | 0.314 ± 0.086 | 0.251 ± 0.086 | 0.318 ± 0.060 | 0.225 ± 0.080 |
| | 9-Octadecenoic acid ester | 0.287 ± 0.073 | 0.230 ± 0.095 | 0.241 ± 0.064 | 0.388 ± 0.340 | 0.250 ± 0.068 | 0.208 ± 0.071 | 0.231 ± 0.041 | 0.167 ± 0.054 |
| | trans-13-Octadecenoic acid | 0.073 ± 0.014 | 0.066 ± 0.022 | 0.060 ± 0.014 | 0.088 ± 0.074 | 0.072 ± 0.018 | 0.052 ± 0.016 | 0.064 ± 0.012 | 0.054 ± 0.017 |
| | Oleic acid | 0.008 ± 0.006 | 0.003 ± 0.001 | 0.006 ± 0.003 | 0.016 ± 0.013 | 0.018 ± 0.021 | 0.019 ± 0.015 | 0.030 ± 0.023 | 0.011 ± 0.009 |
| | Stearic acid | 0.175 ± 0.032 | 0.196 ± 0.115 | 0.157 ± 0.020 | 0.269 ± 0.240 | 0.192 ± 0.039 | 0.182 ± 0.050 | 0.176 ± 0.024 | 0.175 ± 0.043 |
| | Arachidonic acid | 0.011 ± 0.002 | 0.013 ± 0.005 | 0.010 ± 0.004 | 0.014 ± 0.005 | 0.006 ± 0.002 | 0.008 ± 0.005 | 0.005 ± 0.001 | 0.007 ± 0.002 |
| | Eicosanoic acid | 0.008 ± 0.002 | 0.007 ± 0.006 | 0.007 ± 0.003 | 0.014 ± 0.023 | 0.008 ± 0.001 | 0.006 ± 0.001 | 0.005 ± 0.001 | 0.005 ± 0.001 |
| | 1-Monopalmitin | 0.080 ± 0.028 | 0.125 ± 0.029 | 0.099 ± 0.010 | 0.120 ± 0.027 | 0.196 ± 0.022 | 0.206 ± 0.052 | 0.183 ± 0.013 | 0.182 ± 0.024 |
| | 9-Octadecenoic acid, -2-propyl ester | 0.014 ± 0.005 | 0.008 ± 0.004 | 0.013 ± 0.005 | 0.021 ± 0.012 | 0.079 ± 0.049 | 0.066 ± 0.023 | 0.085 ± 0.030 | 0.052 ± 0.023 |
| | 1-Monooleoylglycerol | 0.057 ± 0.025 | 0.039 ± 0.014 | 0.044 ± 0.014 | 0.090 ± 0.052 | 0.427 ± 0.170 | 0.372 ± 0.165 | 0.480 ± 0.078 | 0.298 ± 0.068 |
| | Stearic acid, 2,3-propyl ester | 0.054 ± 0.038 | 0.082 ± 0.038 | 0.054 ± 0.008 | 0.087 ± 0.054 | 0.100 ± 0.032 | 0.137 ± 0.070 | 0.086 ± 0.017 | 0.107 ± 0.037 |
| | **Total** | **1.883** | **1.974** | **1.779** | **2.600** | **2.590** | **2.372** | **2.521** | **2.147** |
| Nitrogenous compounds | Uracil | 0.116 ± 0.052 | 0.096 ± 0.035 | 0.117 ± 0.064 | 0.119 ± 0.034 | 0.007 ± 0.008 | 0.010 ± 0.009 | 0.013 ± 0.009 | 0.018 ± 0.005 |
| | Adenosine | 0.003 ± 0.001 | 0.001 ± 0.001 | 0.003 ± 0.001 | 0.004 ± 0.001 | 0.010 ± 0.004 | 0.014 ± 0.004 | 0.011 ± 0.005 | 0.013 ± 0.003 |
| | **Total** | **0.119** | **0.097** | **0.121** | **0.122** | **0.017** | **0.023** | **0.024** | **0.031** |
| Phenolic acids | m-Hydroxybenzoic acid | 0.080 ± 0.093 | 0.099 ± 0.182 | 0.091 ± 0.088 | 0.017 ± 0.020 | 0.140 ± 0.139 | 0.091 ± 0.132 | 0.124 ± 0.110 | 0.087 ± 0.165 |
| | protocatechuic acid | 0.028 ± 0.056 | 0.033 ± 0.044 | 0.004 ± 0.009 | 0.052 ± 0.076 | 0.024 ± 0.059 | 0.071 ± 0.108 | 0.012 ± 0.027 | 0.036 ± 0.043 |

| Class | Metabolite | Wk0 | | | | Wk4 | | | |
|---|---|---|---|---|---|---|---|---|---|
| | | Wk0_U | Wk0_P | Wk0_B | Wk0_PB | Wk4_U | Wk4_P | Wk4_B | Wk4_PB |
| | 4-Hydroxyhydrocinnamic acid | 0.007 ± 0.001 | 0.005 ± 0.002 | 0.008 ± 0.002 | 0.008 ± 0.003 | 0.014 ± 0.005 | 0.034 ± 0.008 | 0.038 ± 0.023 | 0.019 ± 0.014 |
| | Benzenepropanoic acid, 4-hydroxy-3-methoxy | 0.010 ± 0.003 | 0.004 ± 0.003 | 0.007 ± 0.005 | 0.008 ± 0.006 | 0.004 ± 0.002 | 0.010 ± 0.003 | 0.003 ± 0.001 | 0.016 ± 0.018 |
| | Hydrocaffeic acid | 0.021 ± 0.008 | 0.009 ± 0.003 | 0.014 ± 0.008 | 0.026 ± 0.018 | 0.016 ± 0.018 | 0.029 ± 0.017 | 0.046 ± 0.019 | 0.015 ± 0.013 |
| | **Total** | **0.145** | **0.151** | **0.125** | **0.111** | **0.198 ±** | **0.235** | **0.223** | **0.174** |
| **Sterols** | Coprostanol | 0.069 ± 0.033 | 0.053 ± 0.011 | 0.089 ± 0.064 | 0.053 ± 0.025 | 0.109 ± 0.056 | 0.064 ± 0.026 | 0.102 ± 0.016 | 0.060 ± 0.022 |
| | **Total** | **0.069** | **0.053** | **0.089** | **0.053** | **0.109** | **0.064** | **0.102** | **0.060** |
| **Sugar acids** | L-Threonic acid | 0.076 ± 0.046 | 0.083 ± 0.024 | 0.078 ± 0.028 | 0.043 ± 0.047 | 0.008 ± 0.002 | 0.007 ± 0.007 | 0.007 ± 0.003 | 0.012 ± 0.011 |
| | Ribonic acid | 0.005 ± 0.002 | 0.006 ± 0.001 | 0.005 ± 0.001 | 0.004 ± 0.002 | 0.004 ± 0.001 | 0.004 ± 0.002 | 0.003 ± 0.001 | 0.006 ± 0.004 |
| | **Total** | **0.081** | **0.089** | **0.083** | **0.047** | **0.012** | **0.011** | **0.010** | **0.019** |
| **Sugar alcohols** | L-Threitol | 0.006 ± 0.003 | 0.003 ± 0.001 | 0.005 ± 0.002 | 0.004 ± 0.001 | 0.004 ± 0.004 | 0.004 ± 0.003 | 0.002 ± 0.000 | 0.006 ± 0.004 |
| | Pinitol | 0.238 ± 0.213 | 0.082 ± 0.086 | 0.096 ± 0.135 | 0.016 ± 0.010 | 0.154 ± 0.211 | 0.101 ± 0.121 | 0.008 ± 0.001 | 0.465 ± 0.382 |
| | 1,5-Anhydro-D-sorbitol | 0.007 ± 0.003 | 0.010 ± 0.004 | 0.007 ± 0.003 | 0.006 ± 0.005 | 0.001 ± 0.000 | 0.006 ± 0.014 | 0.001 ± 0.000 | 0.002 ± 0.002 |
| | Ribitol | 0.209 ± 0.037 | 0.168 ± 0.050 | 0.169 ± 0.053 | 0.220 ± 0.100 | 0.040 ± 0.022 | 0.022 ± 0.006 | 0.024 ± 0.007 | 0.034 ± 0.015 |
| | Myoinositol-1 | 0.002 ± 0.000 | 0.002 ± 0.000 | 0.002 ± 0.000 | 0.002 ± 0.001 | 0.001 ± 0.000 | 0.001 ± 0.000 | 0.002 ± 0.004 | 0.002 ± 0.001 |
| | Myoinositol-2 | 0.034 ± 0.022 | 0.013 ± 0.012 | 0.014 ± 0.017 | 0.003 ± 0.001 | 0.014 ± 0.011 | 0.011 ± 0.006 | 0.015 ± 0.011 | 0.050 ± 0.039 |
| | Myoinositol-3 | 0.021 ± 0.041 | 0.023 ± 0.030 | 0.004 ± 0.007 | 0.037 ± 0.051 | 0.020 ± 0.046 | 0.054 ± 0.080 | 0.010 ± 0.022 | 0.029 ± 0.029 |
| | Maltitol | 0.023 ± 0.018 | 0.012 ± 0.003 | 0.011 ± 0.004 | 0.014 ± 0.006 | 0.108 ± 0.030 | 0.277 ± 0.079 | 0.145 ± 0.081 | 0.316 ± 0.301 |
| | **Total** | **0.540** | **0.314** | **0.308** | **0.301** | **0.343** | **0.477** | **0.207** | **0.904** |
| **Sugars** | Glycerol | 0.829 ± 0.106 | 0.875 ± 0.081 | 0.899 ± 0.191 | 1.097 ± 0.187 | 0.410 ± 0.076 | 0.378 ± 0.139 | 0.335 ± 0.074 | 0.393 ± 0.196 |
| | Xylulose | 0.003 ± 0.001 | 0.002 ± 0.001 | 0.002 ± 0.001 | 0.005 ± 0.003 | 0.006 ± 0.004 | 0.008 ± 0.004 | 0.007 ± 0.004 | 0.007 ± 0.006 |
| | Arabinose-1 | 0.183 ± 0.117 | 0.181 ± 0.120 | 0.203 ± 0.104 | 0.141 ± 0.058 | 0.060 ± 0.027 | 0.055 ± 0.066 | 0.055 ± 0.022 | 0.043 ± 0.027 |

| Class | Metabolite | Wk0 | | | | Wk4 | | | |
|---|---|---|---|---|---|---|---|---|---|
| | | Wk0_U | Wk0_P | Wk0_B | Wk0_PB | Wk4_U | Wk4_P | Wk4_B | Wk4_PB |
| | Arabinose-2 | 0.433 ± 0.050 | 0.342 ± 0.093 | 0.378 ± 0.081 | 0.380 ± 0.104 | 0.106 ± 0.030 | 0.061 ± 0.017 | 0.080 ± 0.026 | 0.094 ± 0.022 |
| | Ribose | 0.259 ± 0.082 | 0.193 ± 0.027 | 0.239 ± 0.091 | 0.254 ± 0.055 | 0.046 ± 0.014 | 0.034 ± 0.007 | 0.040 ± 0.010 | 0.063 ± 0.029 |
| | Ribofuranose (isomer 2) | 0.082 ± 0.020 | 0.068 ± 0.021 | 0.070 ± 0.022 | 0.068 ± 0.027 | 0.035 ± 0.012 | 0.022 ± 0.006 | 0.025 ± 0.013 | 0.031 ± 0.012 |
| | Arabinose-3 | 0.681 ± 0.192 | 0.594 ± 0.179 | 0.605 ± 0.099 | 0.643 ± 0.113 | 0.115 ± 0.018 | 0.075 ± 0.051 | 0.150 ± 0.036 | 0.092 ± 0.016 |
| | Fructofuranose (isomer 1) | 0.037 ± 0.010 | 0.034 ± 0.020 | 0.030 ± 0.022 | 0.020 ± 0.004 | 0.006 ± 0.004 | 0.006 ± 0.001 | 0.005 ± 0.002 | 0.010 ± 0.003 |
| | Glucopyranose-1 | 0.080 ± 0.012 | 0.061 ± 0.018 | 0.054 ± 0.026 | 0.070 ± 0.009 | 0.011 ± 0.003 | 0.011 ± 0.003 | 0.010 ± 0.002 | 0.019 ± 0.007 |
| | Glucose | 0.004 ± 0.004 | 0.002 ± 0.001 | 0.004 ± 0.002 | 0.003 ± 0.002 | 0.018 ± 0.011 | 0.020 ± 0.011 | 0.012 ± 0.003 | 0.018 ± 0.012 |
| | Talofuranose (isomer 1) | 0.203 ± 0.181 | 0.211 ± 0.104 | 0.132 ± 0.070 | 0.201 ± 0.141 | 0.036 ± 0.024 | 0.029 ± 0.011 | 0.038 ± 0.017 | 0.093 ± 0.040 |
| | Glucose-1 | 0.020 ± 0.006 | 0.012 ± 0.006 | 0.016 ± 0.004 | 0.020 ± 0.007 | 0.025 ± 0.009 | 0.030 ± 0.012 | 0.033 ± 0.018 | 0.062 ± 0.020 |
| | Glucose-2 | 2.463 ± 0.212 | 1.893 ± 0.235 | 2.252 ± 0.771 | 2.171 ± 0.538 | 0.303 ± 0.147 | 0.234 ± 0.058 | 0.339 ± 0.096 | 0.675 ± 0.259 |
| | Glucopyranose-2 | 0.478 ± 0.056 | 0.411 ± 0.118 | 0.387 ± 0.103 | 0.517 ± 0.230 | 0.093 ± 0.044 | 0.053 ± 0.014 | 0.060 ± 0.019 | 0.080 ± 0.028 |
| | Glucopyranose | 3.403 ± 0.182 | 2.548 ± 0.437 | 3.181 ± 1.076 | 2.909 ± 0.608 | 0.426 ± 0.190 | 0.337 ± 0.080 | 0.487 ± 0.122 | 0.956 ± 0.344 |
| | Trehalose | 0.008 ± 0.002 | 0.006 ± 0.002 | 0.008 ± 0.002 | 0.008 ± 0.002 | 0.005 ± 0.002 | 0.005 ± 0.002 | 0.007 ± 0.003 | 0.006 ± 0.002 |
| | Cellobiose (isomer 1) | 0.049 ± 0.015 | 0.034 ± 0.025 | 0.068 ± 0.036 | 0.047 ± 0.013 | 0.057 ± 0.030 | 0.053 ± 0.011 | 0.087 ± 0.028 | 0.061 ± 0.032 |
| | Cellobiose (isomer 2) | 0.066 ± 0.022 | 0.048 ± 0.039 | 0.106 ± 0.067 | 0.058 ± 0.024 | 0.096 ± 0.048 | 0.088 ± 0.018 | 0.144 ± 0.057 | 0.097 ± 0.049 |
| | **Total** | **9.282** | **7.517** | **8.636** | **8.612** | **1.856** | **1.499** | **1.915** | **2.803** |

**Table S2:** Updated names of some bacterial phyla according to the International Code of Nomenclature of Prokaryotes (ICNP)_taxonomy 2022*

| SILVA v. 123 phylum name | Updated name (ICNP 2022) |
|---|---|
| Firmicutes | Bacillota |
| Proteobacteria | Pseudomonadota |
| Actinobacteria | Actinomycetota |
| Bacteroidetes | Bacteroidota |
| Tenericutes | Mycoplasmatota |
| Verrucomicrobia | Verrucomicrobiota |
| Fusobacteria | Fusobacteriota |

* **Source:** Oren A, Garrity GM. Valid publication of the names of forty-two phyla of prokaryotes. *Int J Syst Evol Microbiol* (2021) 71(10). Epub 2021/10/26. doi: 10.1099/ijsem.0.005056.

**Table S3:** List of used R packages

| R package | Main use |
|---|---|
| ggplot2 and ggpubr | Used for generating box and beanplots (Figures 2E, 3, S1, S2), with statistical tests included in the plots |
| dunn.test | Alternative *post hoc* test for multiple comparisons of non-parametric data |
| pheatmap | Used for generating heatmaps (Figures 11 and S14-S16) |
| factoextra | Used for calculating principal components and plotting PCA and loading plots |
| coin | For permutational-based statistical tests (in case of ties) |

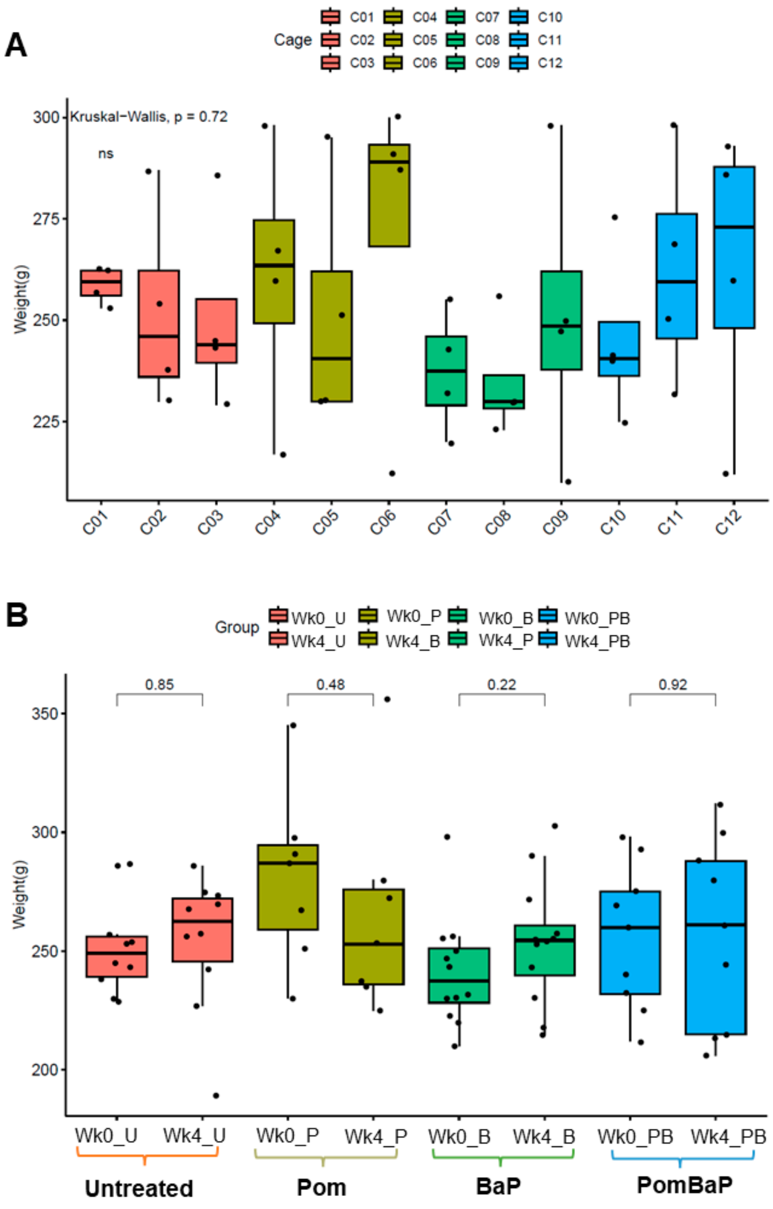


**Figure S1. Animal weight and weight variation. A.** Boxplots representing the weights of 48 Sprague-Dawley rats, measured at week 0, plotted against the 12 cages (n = 4 rats per cage). Kruskal-Wallis *p* value is indicated: no significant difference is observed between the animal weights per cage. **B.** Change in rat weights with between week 0 and week 4 in different treatment groups (only rats that survived the entire experiment are compared). Data were analyzed for significance by paired t-test, and *p* values are indicated for each pair of groups.

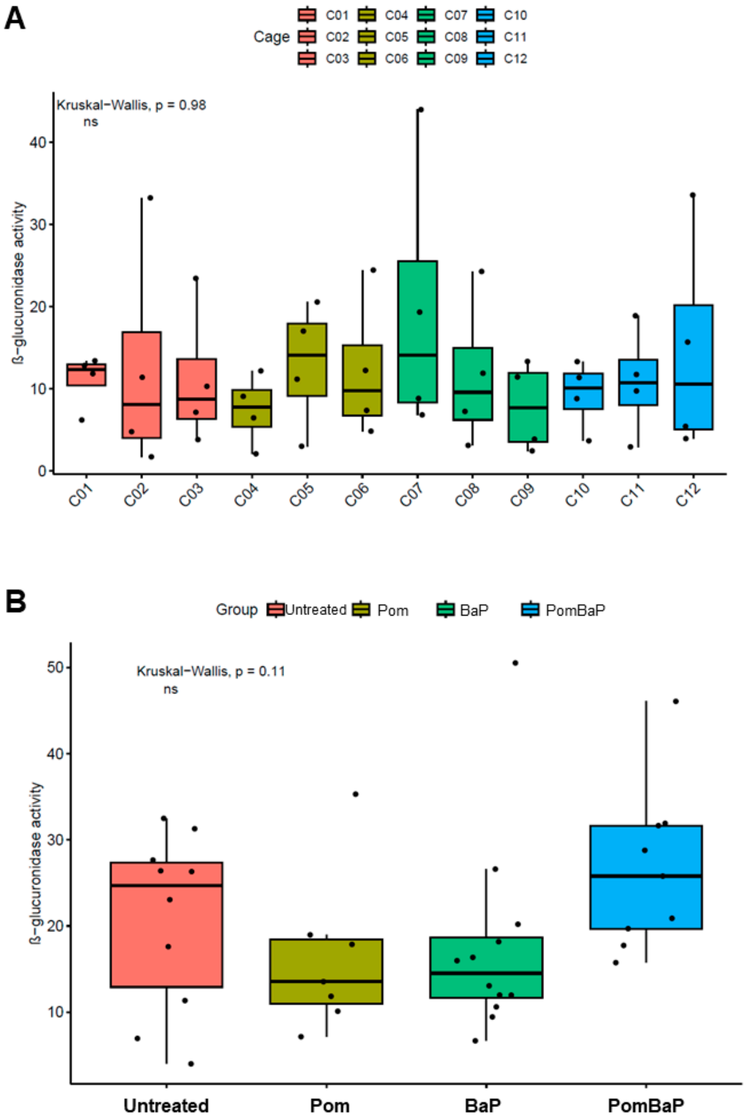


**Figure S2. Fecal glucuronidase activity at the start and end of the experiment. A.** Boxplots representing the fecal glucuronidase activity (expressed as mg of PNPG/mg feces per minute), determined in week 0, of 48 Sprague-Dawley rats, randomized over 12 cages (n = 4 rats per cage). Statistical significance was assessed by the Kruskal-Wallis test. No *post hoc* comparisons were conducted since the medians were not significantly different between cages. **B.** β-Glucuronidase enzyme activity in different treatment groups (only rats that survived till the end of the experiment), determined on week 4, no significant difference was observed between the groups, and thus no *post hoc* comparisons were conducted.

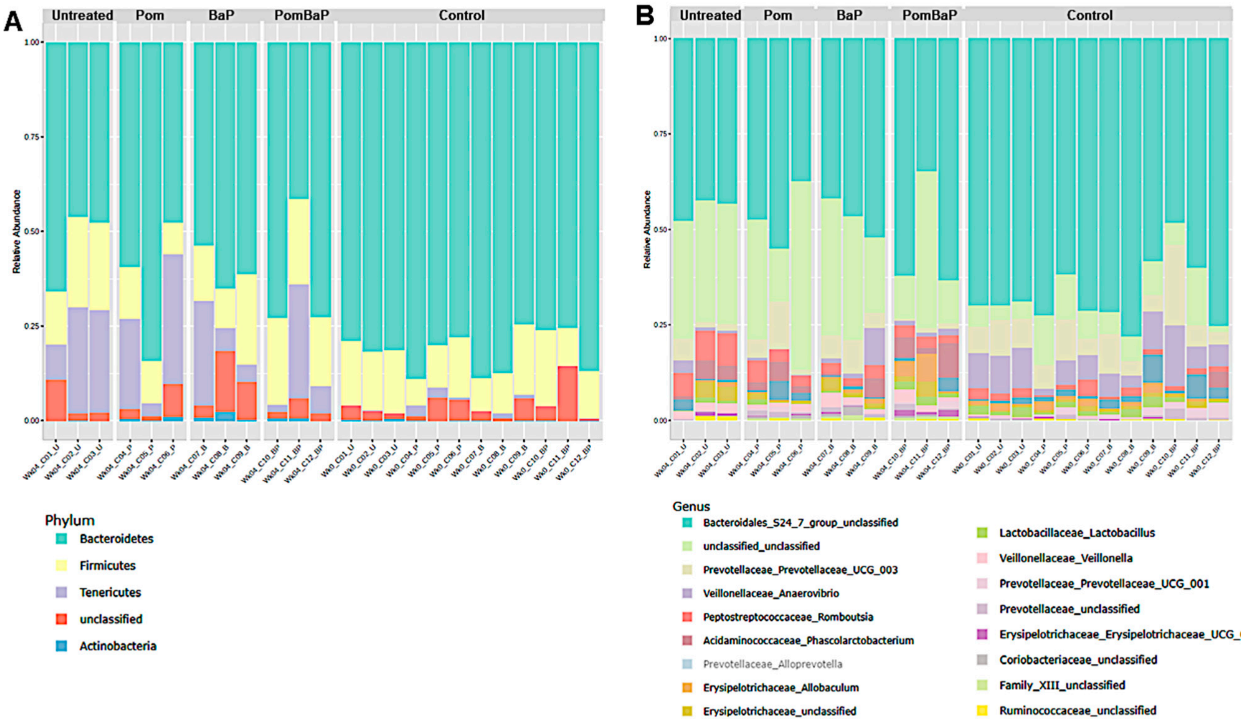


**Figure S3. Taxonomic profile of the fecal microbiome of experimental animals.** Summary of the relative abundance of different taxonomic units among microbial communities detected in the fecal samples at the phylum **(A)** and genus **(B)** levels.

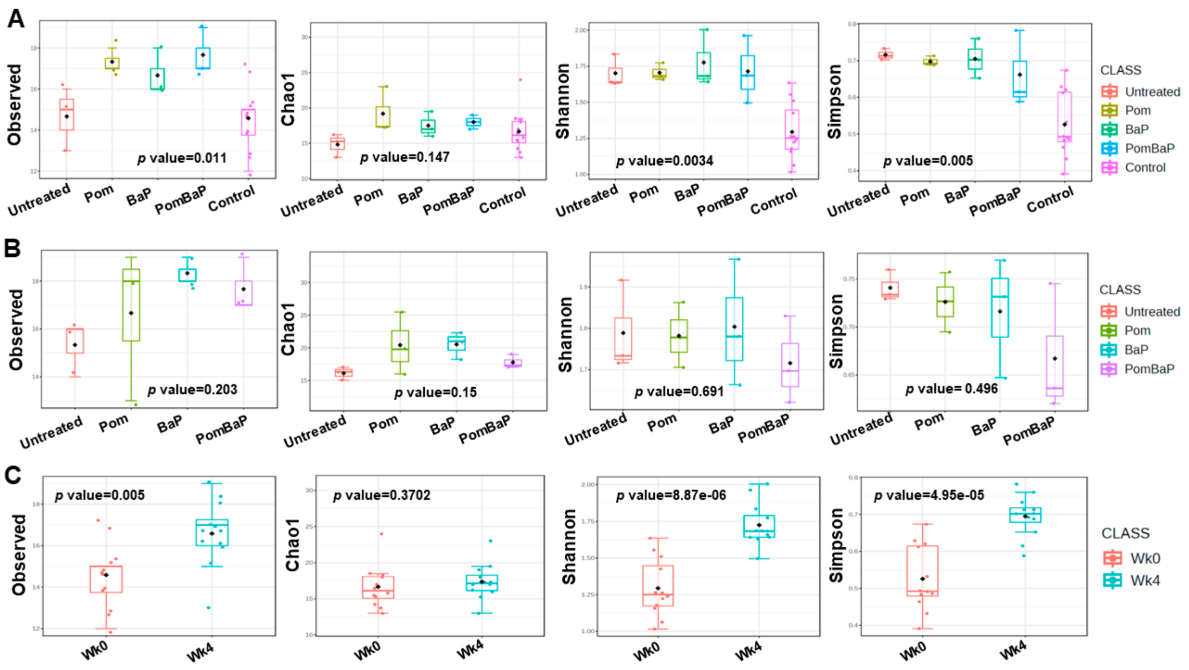


**Figure S4. Different alpha diversity metrics (observed OTUs, Chao1, Shannon, and Simpson diversity indices), for microbiome samples at genus level, in different treatment groups,** including control samples **(A)**, different treatment groups after excluding the control samples **(B),** and between the two age groups, Wk0 and Wk4 **(C)**.

Differences were analyzed for statistical significance by the Kruskal-Wallis test. *Post hoc* pairwise Wilcoxon tests for Shannon and Simpson indices **(A panels)**, using FDR *p* value adjustment, indicated that pairwise comparisons between the control and each of the following treatment groups: Untreated, Pom, and BaP, were statistically significant (*p* value = 0.029, 0.022, and 0.022, respectively for Shannon index; and 0.022, 0.022, and 0.029, respectively for Simpson index); while the difference between control and PomBaP groups was not statistically significant (Wilcoxon test *p* value = 0.077 for Shannon index and *p* value = 0.341, for Simpson index). Pairwise comparisons between the control and each of the following treatment groups: Pom, BaP, and PomBaP were statistically significant (*p* value = 0.029, 0.048, 0.024), while the difference between the control and untreated groups was not statistically significant (*p* value = 0.926) for observed OTUs.

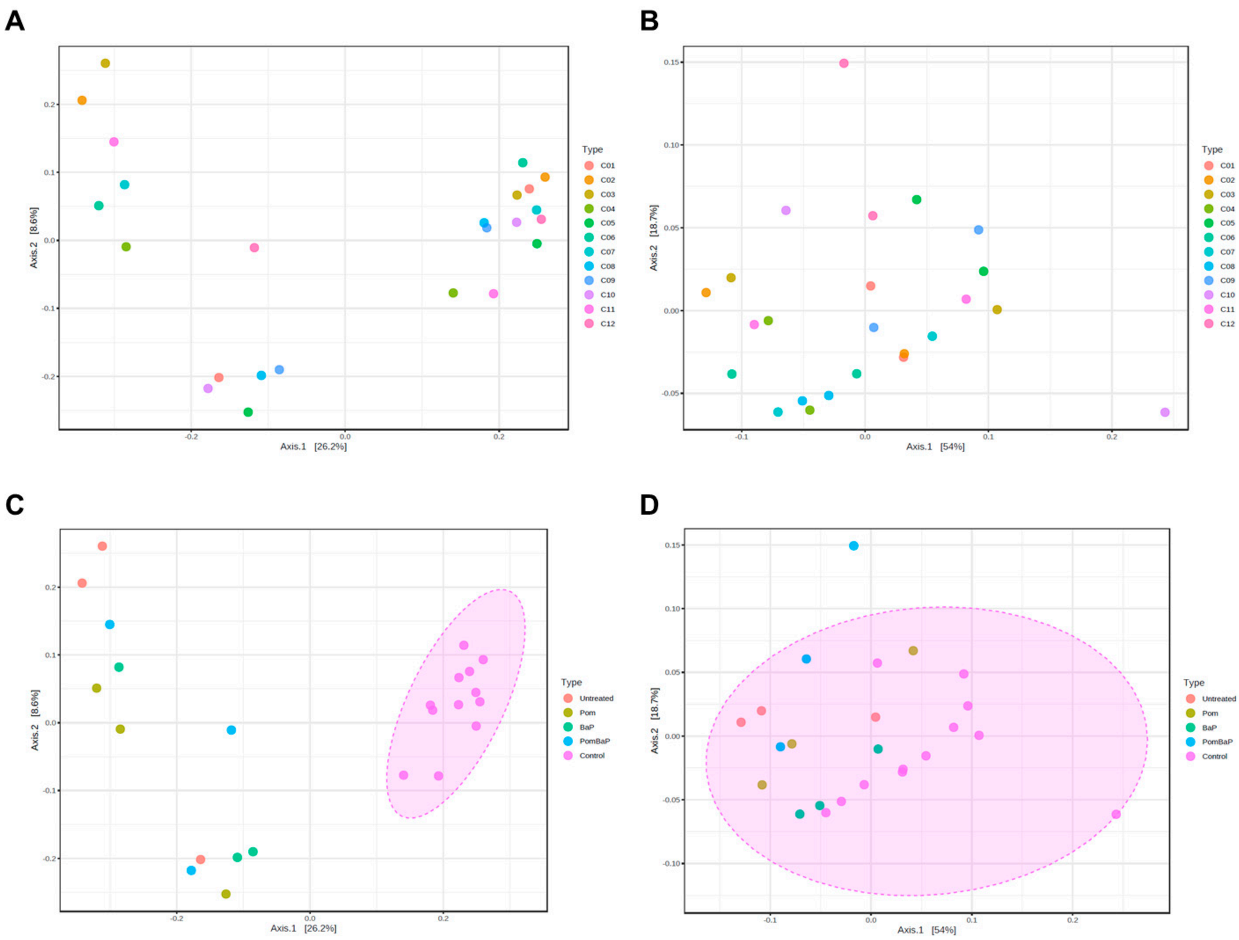


**Figure S5. Microbiome beta-diversity analysis,** visualized by PCA of Bray-Curtis distances, in which each sample is colored by the cage it was sampled from **(A,B)**. **A.** The distances are calculated at the feature level —PERMANOVA F-value = 0.665, R-squared = 0.379, *p* value: 0.999. **B.** The distances are calculated at the genus level—PERMANOVA F-value = 0.833, R-squared = 0.433, *p* value = 0.718. No significant cage-based clustering was observed in either case. Corresponding group-based clusters (Fig. 4) from feature- and genus-level analysis (**C and D,** respectively) are shown for comparison.

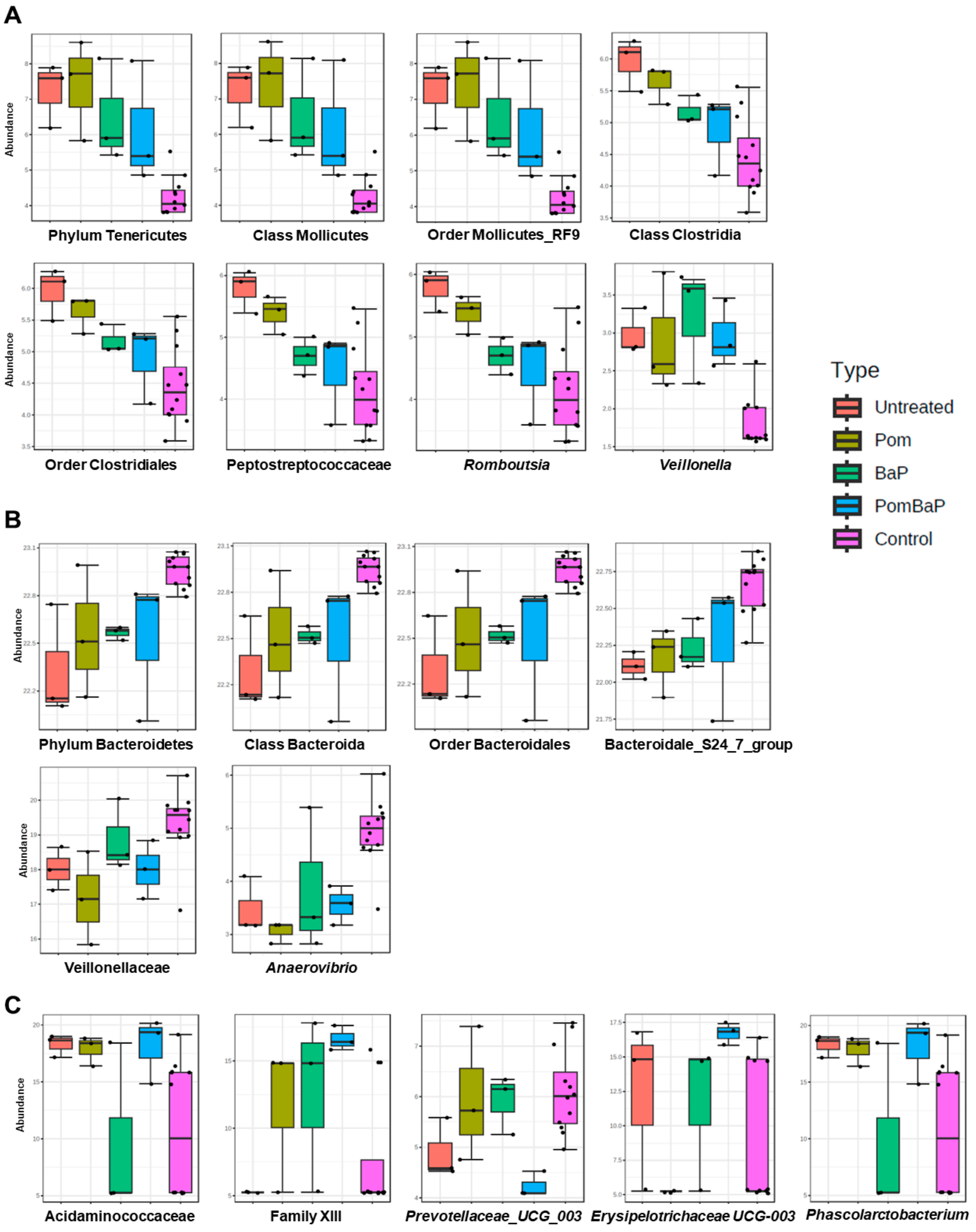


**Figure S6. Full list of taxa with significant differences in relative abundance among the five groups.** Boxplots describing differential relative abundance of nineteen significant taxa in different samples (influenced by both age and treatment factors). The significance was identified by linear discriminant analysis effect size (LEfSe) analysis, with *p* value cutoff of 0.05, and *p* value adjustment by the FDR method. **Y-axis:** Relative abundance expressed as log-transformed counts.

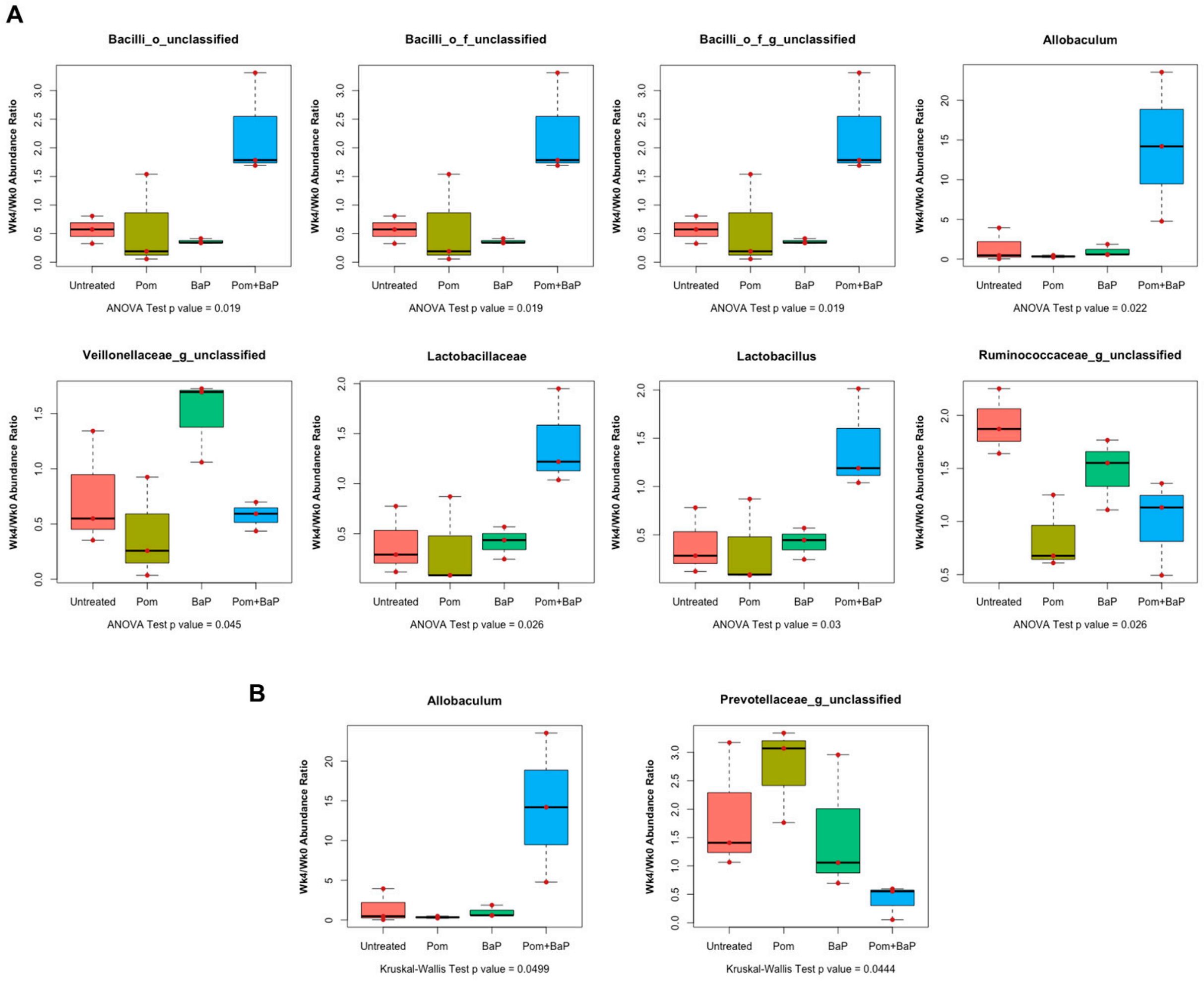


**Figure S7. Significantly different taxa between different treatment groups of week 4 (n = 3 cages per group), normalized to their corresponding week 0 samples.**
**A**. Significantly different taxa analyzed by ANOVA (*p* value < 0.05). **B.** Significantly different taxa analyzed by the Kruskal-Wallis test (*p* value < 0.05).

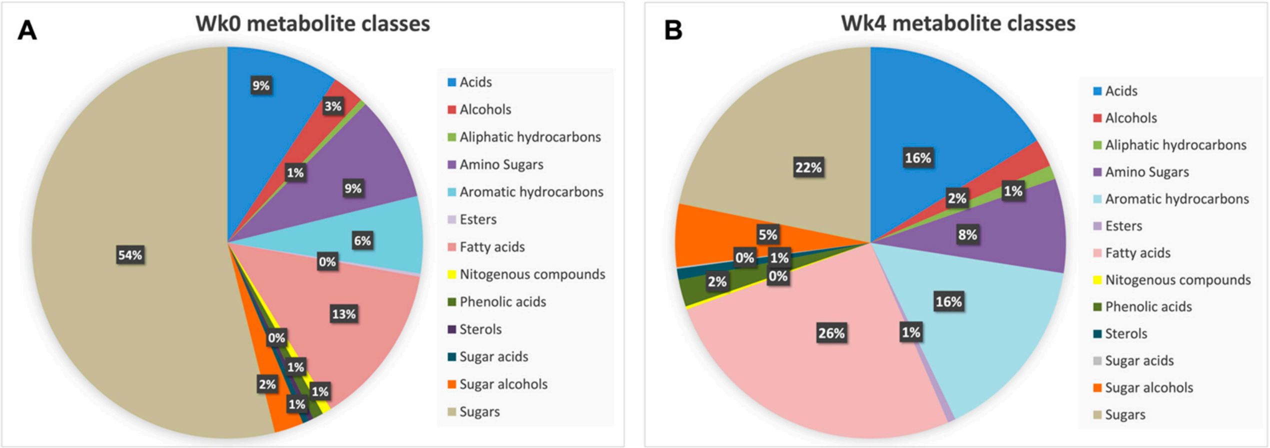


**Figure S8. Distribution of the different detected metabolite classes**, normalized to xylitol, from rat feces collected on week 0 **(A)** and week 4 **(B)**.

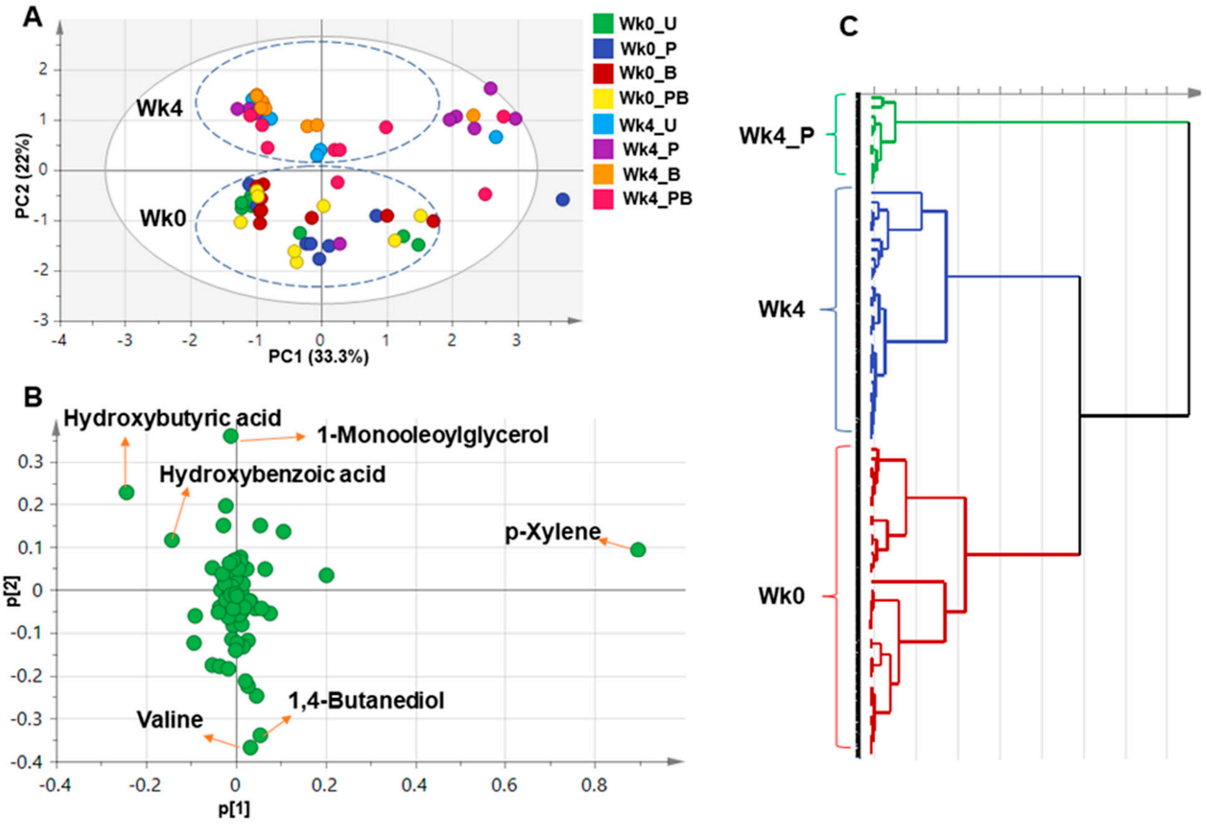


**Figure S9. PCA for four sample groups from the start and end of the experiment: four groups representing week 0, and four representing week 4), with sugars removed.** This model was explained by 7 components with total variance = 0.855 and prediction power = 0.183). **A.** Score plot of PC1 vs. PC2, which both explain 55% of the variance. A complete separation of week 0 from week 4 samples is observed. **B.** Loading plot for PC1 and PC2 showing the major metabolites (trimethylsilylated) contributing to sample clustering. **C.** Hierarchical clustering analysis (HCA) of GC dataset shows three main clusters. **U** = untreated, **P** = pomegranate, **B** = benzo[a]pyrene, **PB** = pomegranate+benzo[a]pyrene

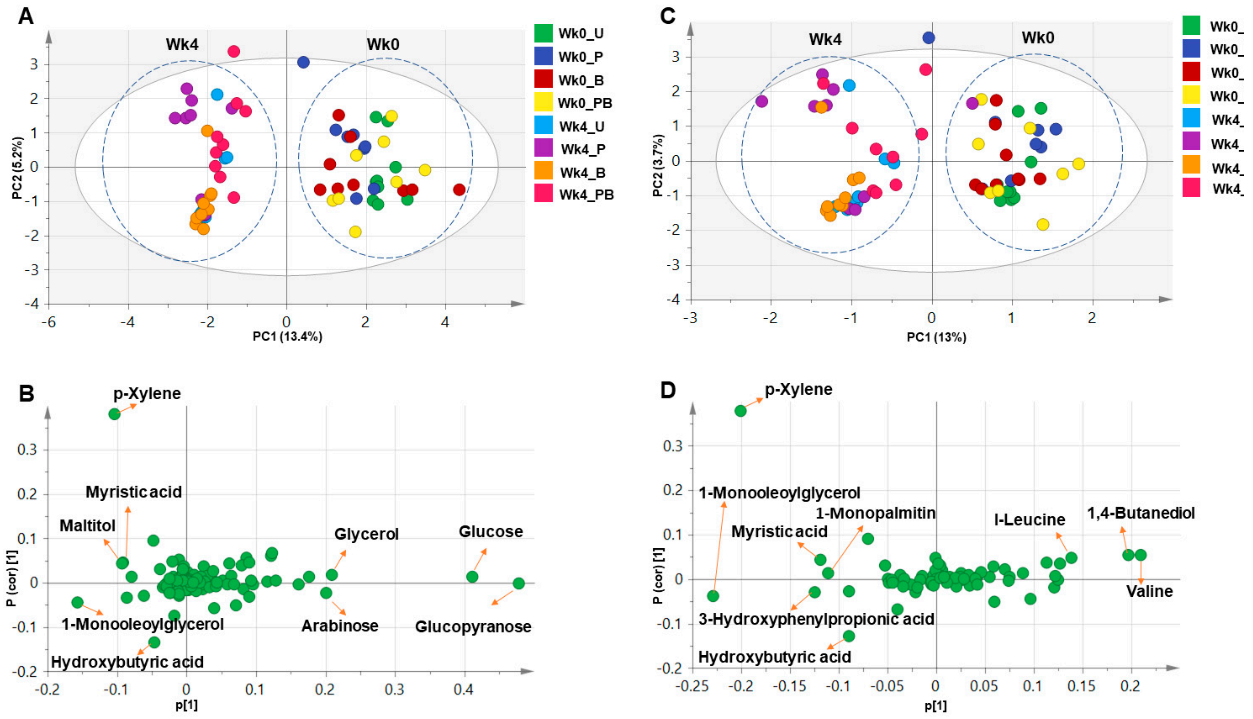


**Figure S10. OPLS analyses of the sample groups, including and excluding sugars.**

**A-B.** OPLS for eight sample groups (4 representing Wk0, and 4 representing Wk4).
**A.** Score plot of PC1 vs. PC2, complete separation of Wk0 from Wk4 samples,this model was explained by 7 components with total variance = 0.479 and prediction power = 0.181). **B.** S-Plot for PC1 and PC2 showing the major metabolites contributing to sample clustering.
**C-D:** OPLS for eight sample groups, with class 'Sugars' excluded- (4 representing Wk0, and 4 representing Wk4). **C.** Score plot of PC1 vs. PC2, complete separation of Wk0 from Wk4 samples, this model was explained by 7 components with total variance=0.414 and prediction power = 0.104. **D.** S-Plot for PC1 and PC2 showing the major metabolites (trimethylsilylated) contributing to sample clustering. **U** = untreated, **P** = pomegranate, **B** = benzo[a]pyrene, **PB** = pomegranate+benzo[a]pyrene.

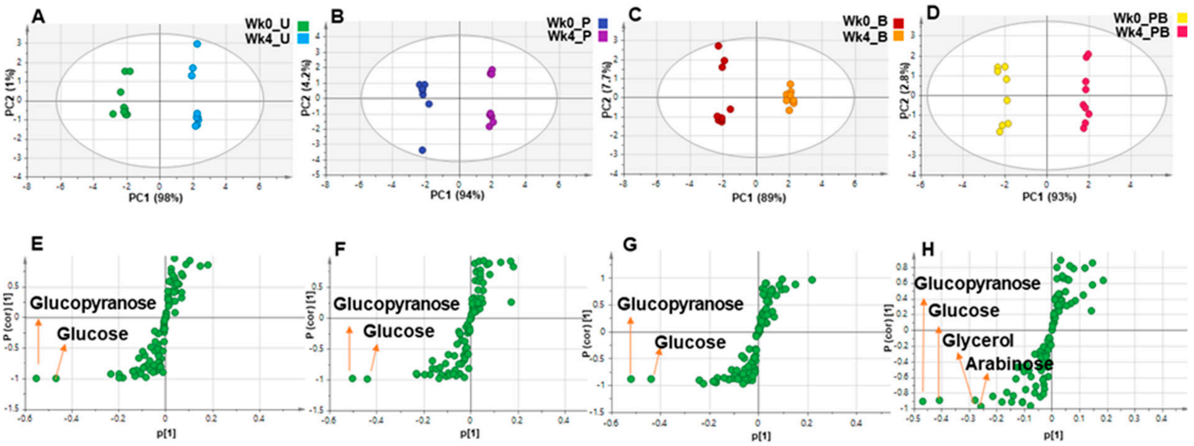


**Figure S11. OPLS-DA for normalized groups before (Wk0) and at the end (Wk4) of the experiment:** untreated, Pom, BaP, and POM+BaP groups (**A, B, C, and D,** respectively). S-Plots with the major metabolites (trimethylsilylated) contributing for the variability between Wk0 and Wk4 of the untreated, Pom, BaP, and POM+BaP groups (**E, F, G, and H** respectively). **U** = untreated, **P** = pomegranate, **B** = benzo[a]pyrene, **PB** = pomegranate+benzo[a]pyrene

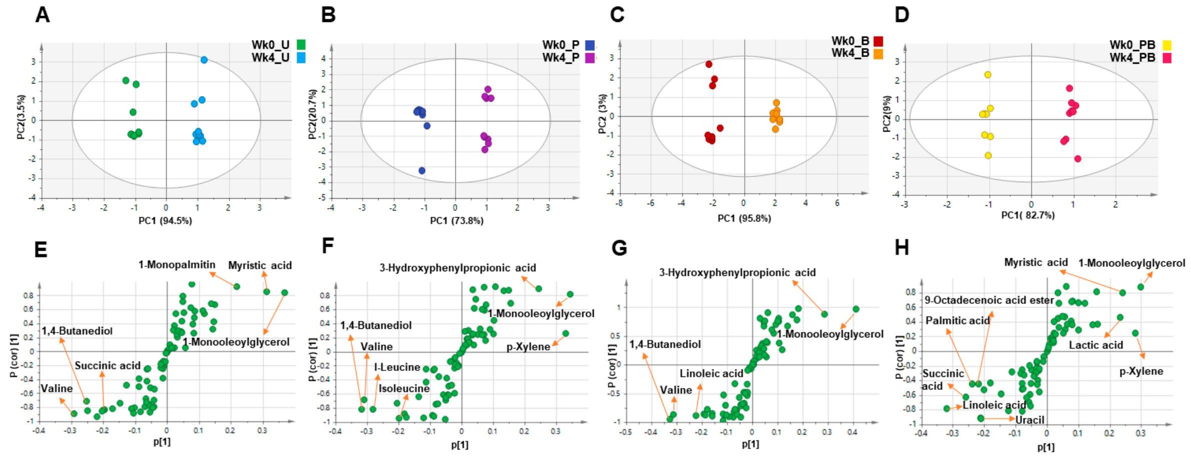


**Figure S12. OPLS-DA for normalized groups before (Wk0) and at the end (Wk4) of the experiment—with sugars excluded:** untreated, Pom, BaP, and POM+BaP groups (**A, B, C, and D**, respectively). S-Plots with the major metabolites (trimethylsilylated) contributing for the variability between Wk0 and Wk4 of the untreated, Pom, BaP, and POM+BaP groups (**E, F, G, and H** respectively). **U** = untreated, **P** = pomegranate, **B** = benzo[a]pyrene, **PB** = pomegranate+benzo[a]pyrene

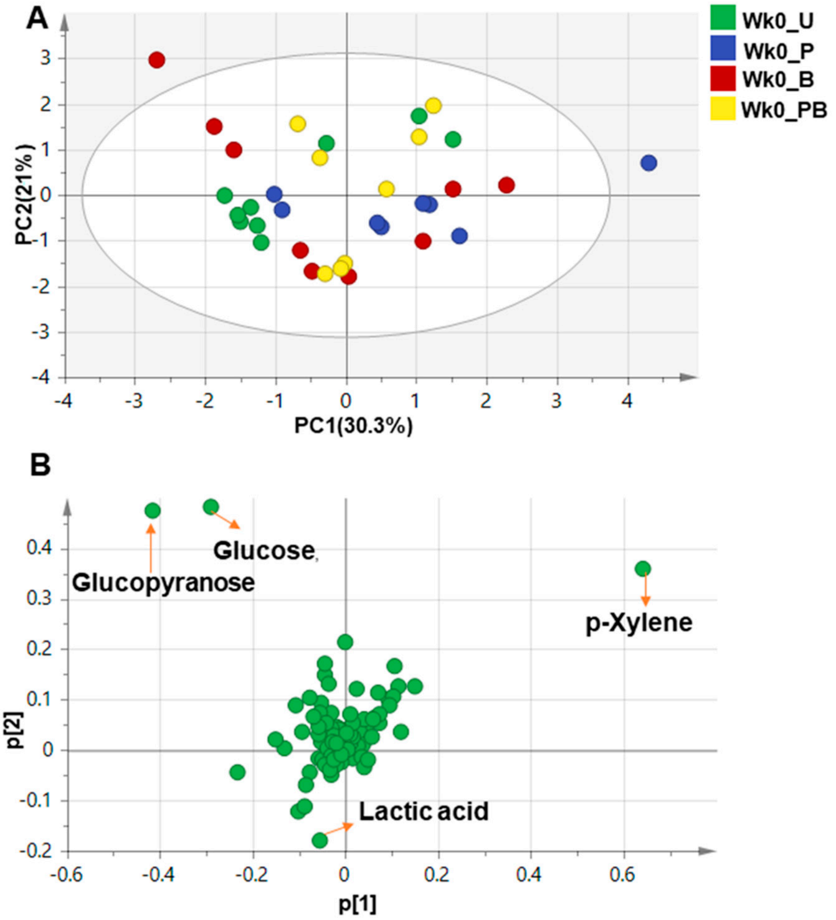


**Figure S13. PCA for four sample groups, representing Wk0.** This model was explained by seven components with total variance = 0.87 and prediction power = 0.35. **A**: Score plot of PC1 vs. PC2, no clear segregation between the examined samples. **B**: Loading plot for PC1 and PC2 showing the major metabolite (trimethylsilylated) contributing to the divergence of the outlier samples. **U** = untreated, **P** = pomegranate, **B** = benzo[a]pyrene, **PB** = pomegranate+benzo[a]pyrene.

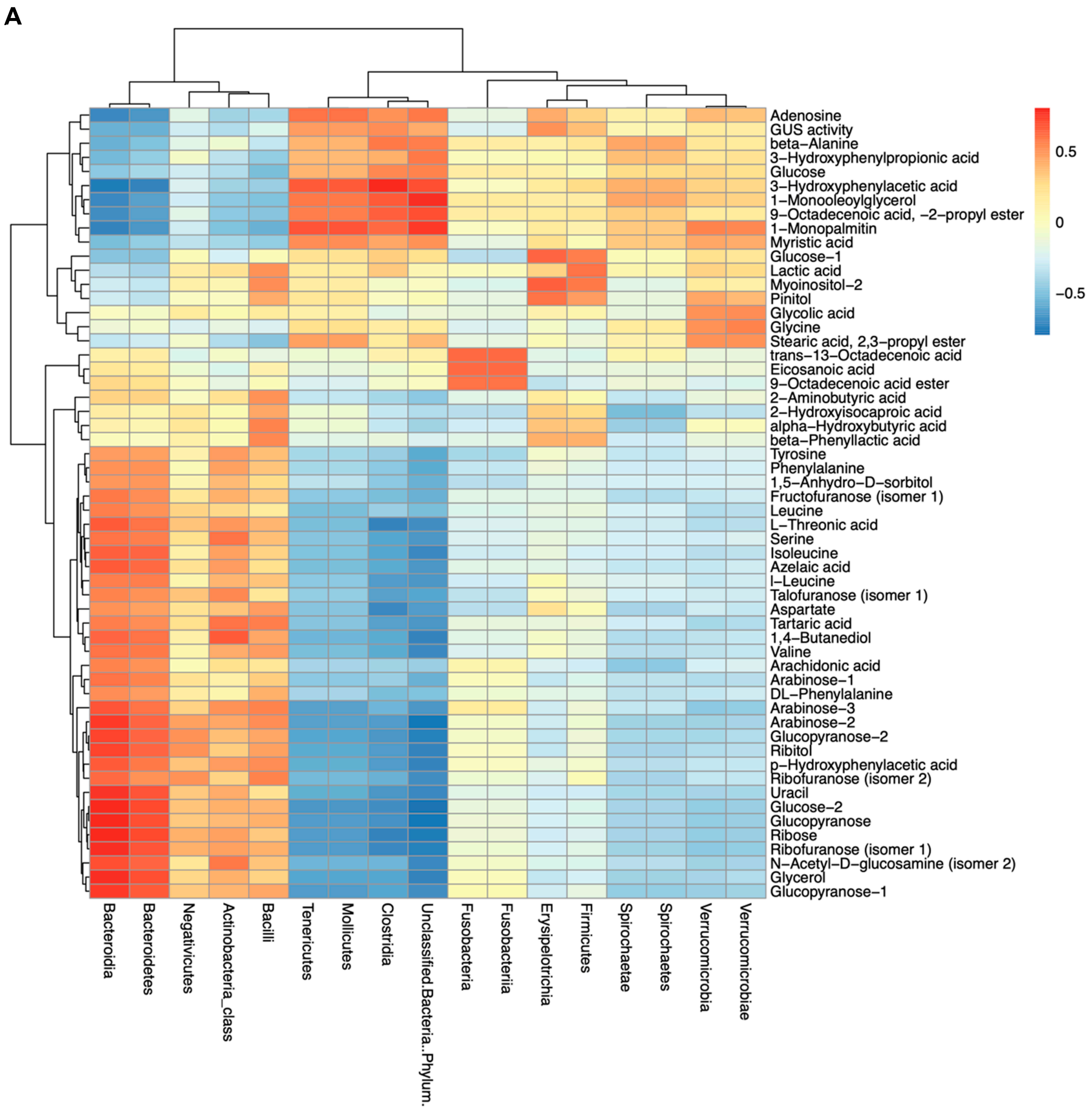


**Figure S14. Microbiome-metabolome correlations.** Correlation between microbiome profiles (% abundance of different taxa) and metabolome normalized levels was calculated by the Spearman correlation method. **A.** Correlation between microbial phyla/classes and metabolites. Correlation between microbial phyla/classes and metabolite classes is displayed in **Fig. 11B.** The heatmap is colored based on Spearman correlation coefficients $r_s$.

**B**

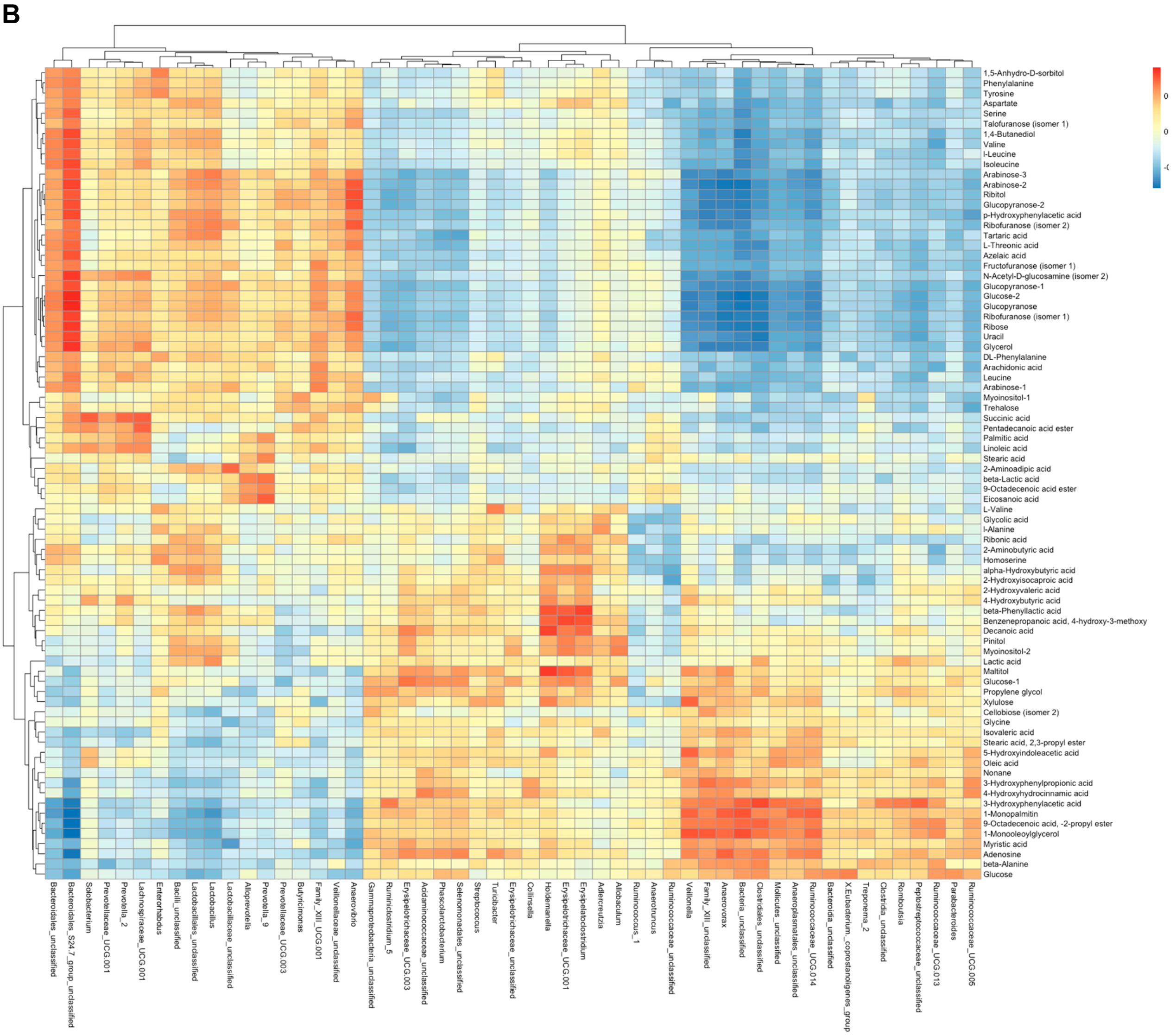


**Figure S14. Microbiome-metabolome correlations.** Correlation between microbiome profiles (% abundance of different taxa) and metabolome normalized levels was calculated by the Spearman correlation method. **B.** Correlation between microbial genera and metabolites. The heatmap is colored based on Spearman correlation coefficients $r_s$.

C

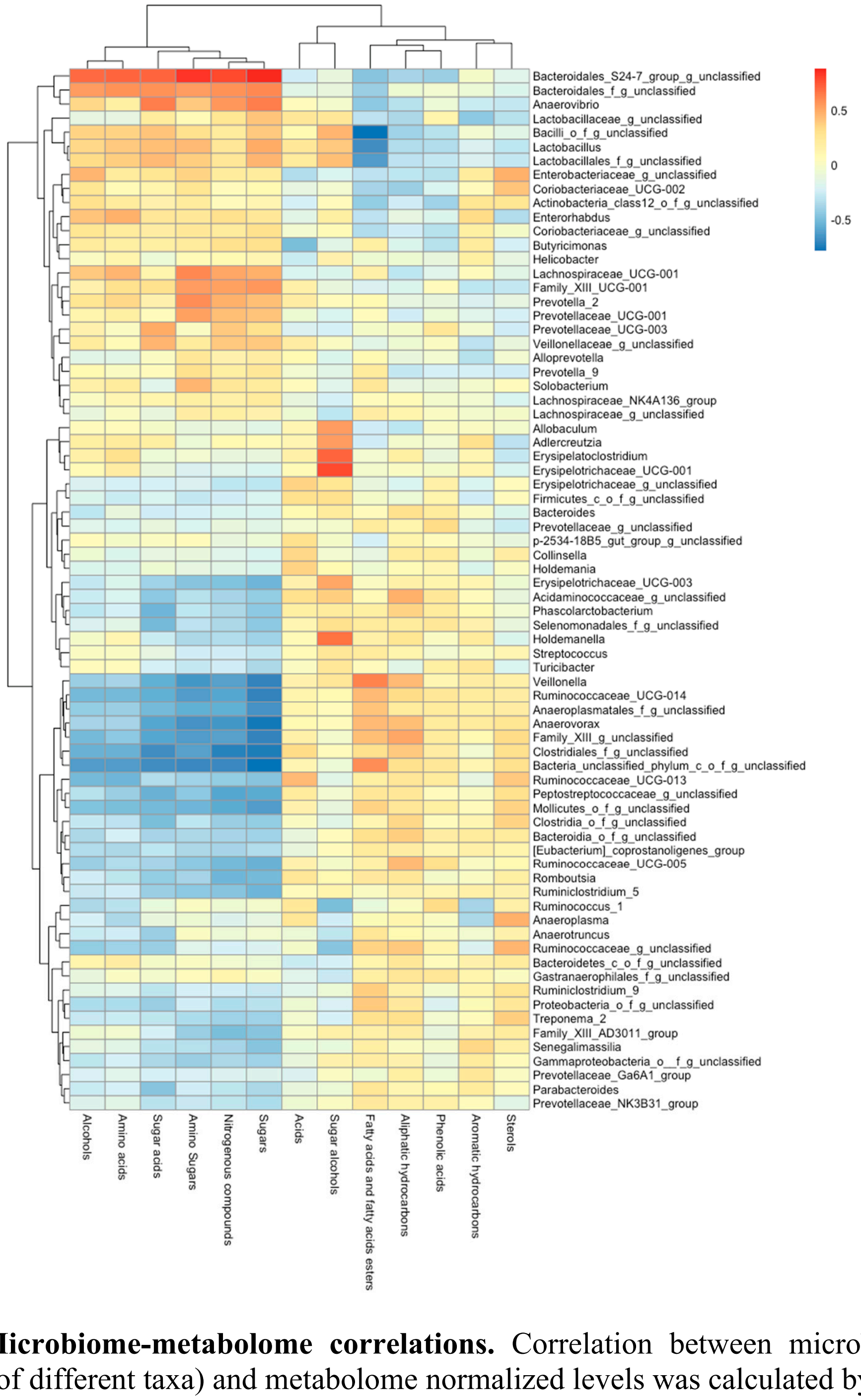


**Figure S14. Microbiome-metabolome correlations.** Correlation between microbiome profiles (% abundance of different taxa) and metabolome normalized levels was calculated by the Spearman correlation method. **C.** Correlations between microbial genera and metabolite classes. Correlation between microbial phyla/classes and metabolite classes is displayed in **Fig. 11B.** The heatmap is colored based on Spearman correlation coefficients $r_s$.

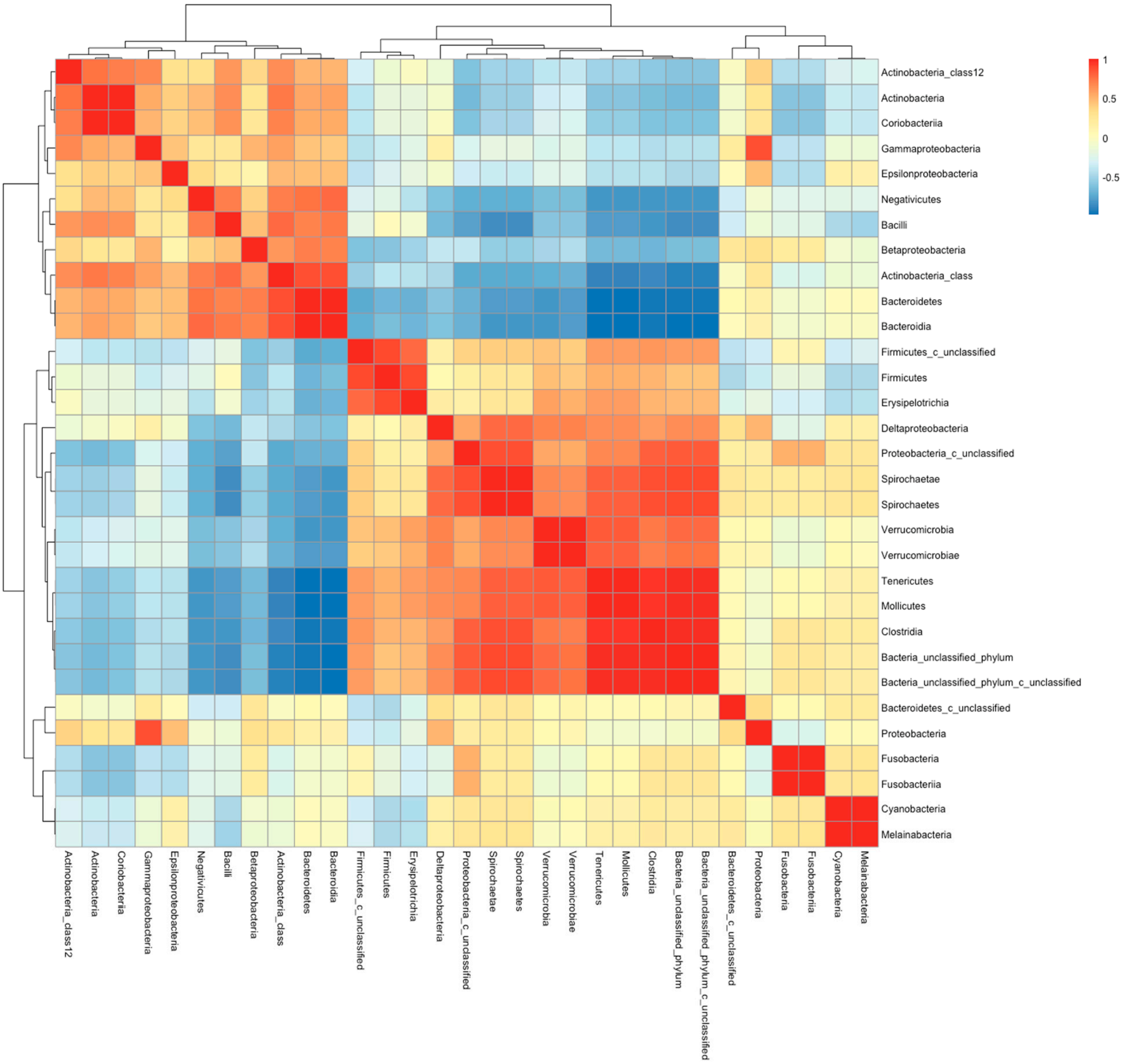


**Figure S15. Correlation matrix of microbial phyla and classes based on their correlation with metabolites.** The heatmap is colored based on Spearman correlation coefficients $r_S$.

**A**

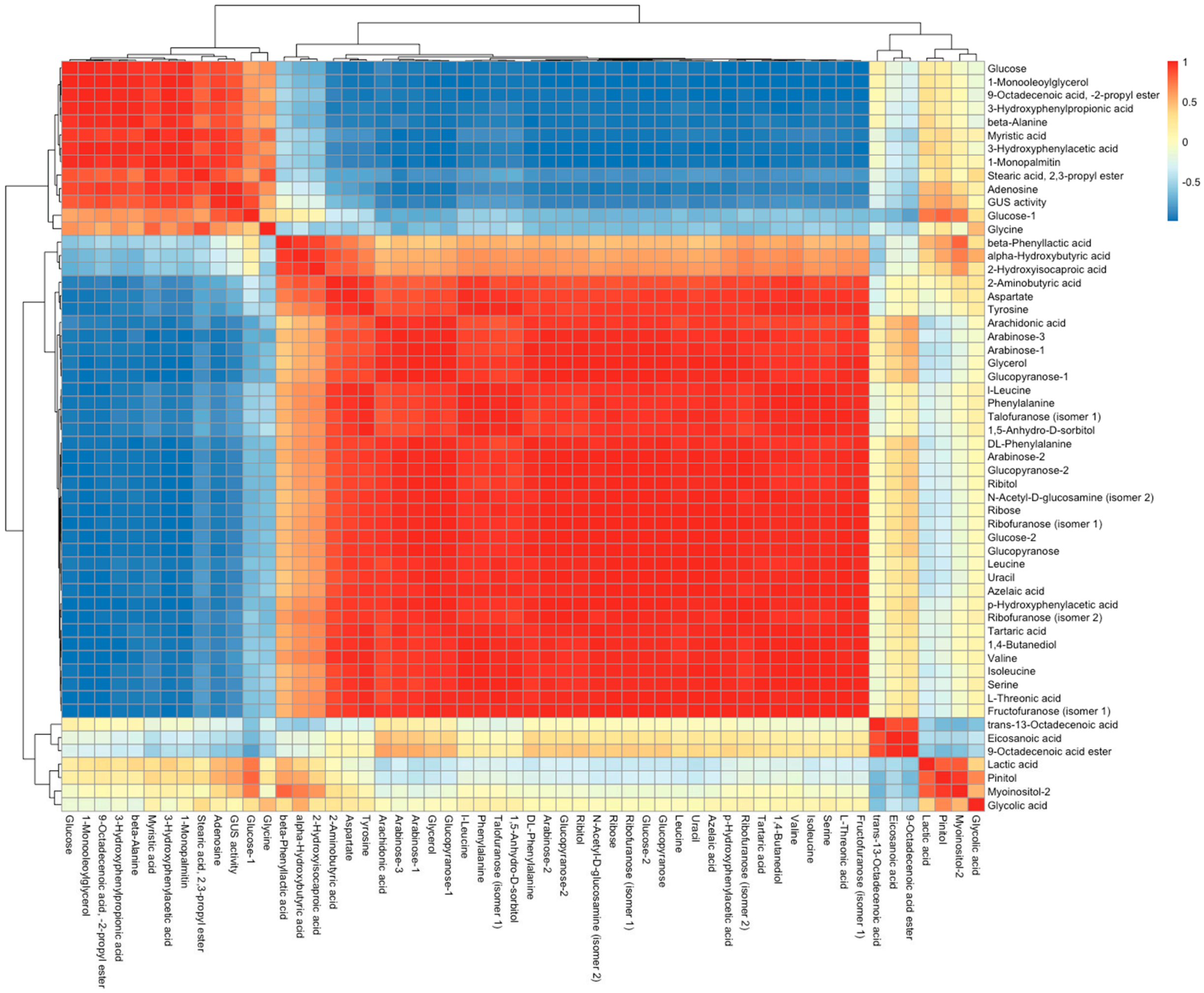


**Figure S16. Correlation matrix of metabolites based on their correlation with microbial taxa.**
**A.** Correlation between metabolites based on their correlation with phyla/classes.
The heatmap is colored based on Spearman correlation coefficients $r_s$.

**B**

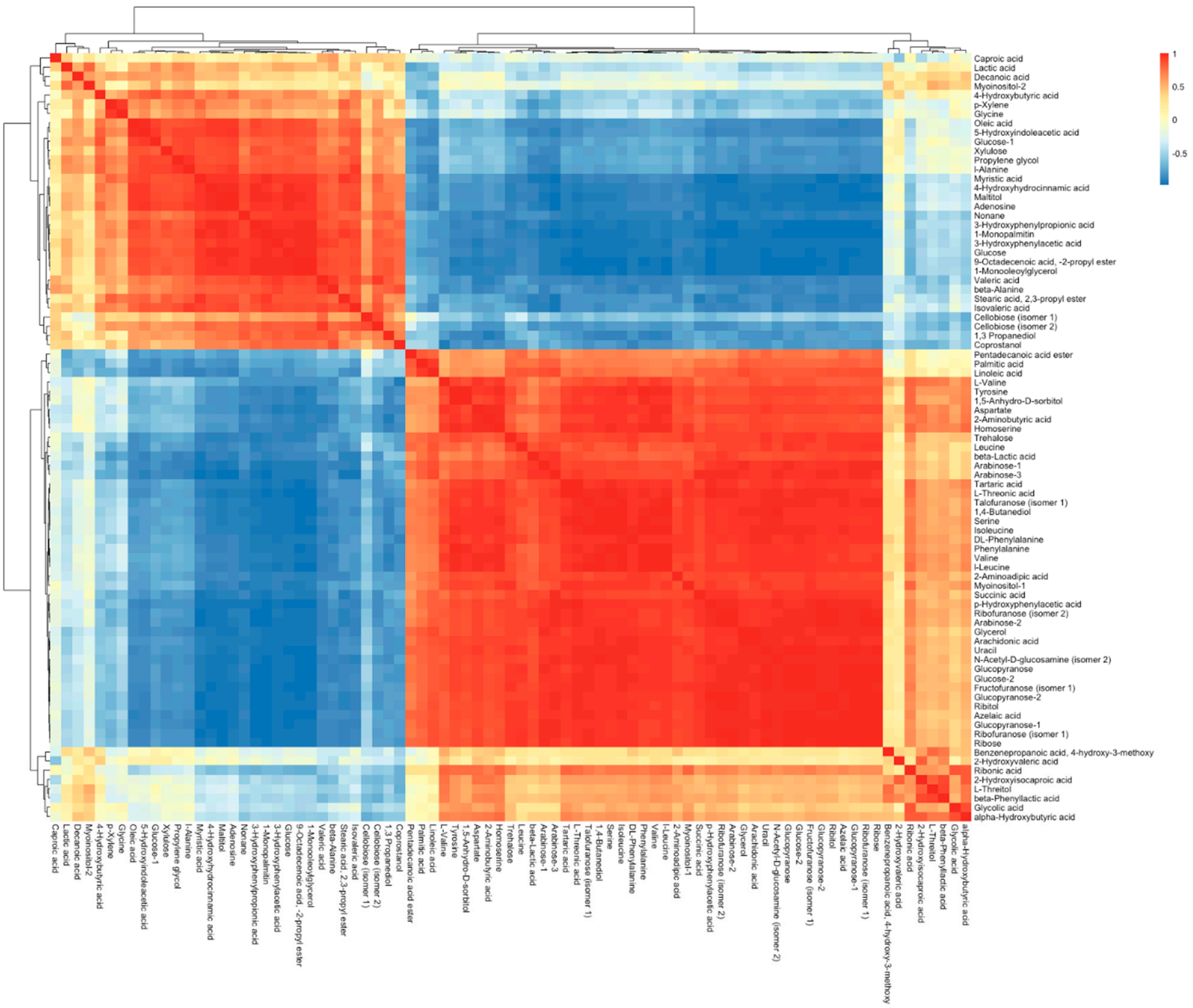


**Figure S16. Correlation matrix of metabolites based on their correlation with microbial taxa. B.** Correlation between metabolites based on their correlation with bacterial genera. The heatmap is colored based on Spearman correlation coefficients $r_S$.
